# Integrative taxonomy reveals previously undescribed diversity within the *Gloydius himalayanus* complex (Squamata, Viperidae, Crotalinae) from the Himalaya and Hindu Kush

**DOI:** 10.3897/zookeys.1280.182768

**Published:** 2026-05-21

**Authors:** Daniel Jablonski, Frank Tillack, Kristin Mahlow-Tillack, Alice Petzold, Madita Wilzo, Abhijit Das, Muhammad Idrees, Chitra B. Baniya, Rafaqat Masroor, Sylvia Hofmann

**Affiliations:** 1 Department of Zoology, Comenius University in Bratislava, Ilkovičova 6, Mlynská dolina, 842 15, Bratislava, Slovakia Zoological Sciences Division, Pakistan Museum of Natural History Islamabad Pakistan; 2 Museum für Naturkunde, Leibniz-Institut für Evolutions- und Biodiversitätsforschung, Invalidenstraße 43, 10115 Berlin, Germany Central Department of Botany, Tribhuvan University Kathmandu Nepal; 3 Institute for Biochemistry and Biology, University of Potsdam, Karl-Liebknecht-Str. 24–25, 14476 Potsdam, Germany Department of Zoology, University of Peshawar Peshawar Pakistan; 4 Wildlife Institute of India, Chandrabani, Dehradun 248001, Uttarakhand, India Institute for Biochemistry and Biology, University of Potsdam Potsdam Germany; 5 Department of Zoology, University of Peshawar, Peshawar 25120, Khyber Pakhtunkhwa, Pakistan Museum Koenig, Leibniz Institute for the Analysis of Biodiversity Change Bonn Germany; 6 Central Department of Botany, Tribhuvan University, Kirtipur, Kathmandu, Nepal Museum für Naturkunde, Leibniz-Institut für Evolutions- und Biodiversitätsforschung Berlin Germany; 7 Zoological Sciences Division, Pakistan Museum of Natural History, Shakarparian, Islamabad, Pakistan Department of Zoology, Comenius University in Bratislava Bratislava Slovakia; 8 Museum Koenig, Leibniz Institute for the Analysis of Biodiversity Change, Adenauerallee 127, 53113 Bonn, Germany Wildlife Institute of India Chandrabani India

**Keywords:** Integrative taxonomy, morphology, museomics, molecular systematics, Serpentes, South Asia, species delimitation

## Abstract

The Himalaya and Hindu Kush remain among the least scientifically explored mountain regions of Asia. Within their vertebrate fauna, pitvipers of the *Gloydius himalayanus* (Günther, 1864) complex have long presented taxonomic challenges due to limited sampling in remote areas and the absence of integrative analyses. Here, we present a comprehensive taxonomic revision of the *G. himalayanus* complex using an integrative framework that combines genetic and distributional data, external morphology, osteology, and ecological evidence. Our dataset also uniquely incorporates both newly collected material and DNA sequences obtained from 19^th^- and early 20^th^-century museum specimens, including one of the syntypes of *G. himalayanus* (BMNH 1946.1.19.64), underscoring the enduring value of historical collections in modern taxonomy. Analyses of four mitochondrial and three nuclear genes from populations across the western and central Himalaya and the Hindu Kush, many of which were genetically studied for the first time, recover five well-supported monophyletic lineages: *G. himalayanus* sensu stricto, *G. chambensis*, and three previously unrecognised lineages from the Hindu Kush of north-western Pakistan, the Hazara region of north-eastern Pakistan at the western margin of the Himalaya, and the Himalaya of western and central Nepal. Genetic distances in protein-coding mtDNA markers indicate species-level divergence among these lineages, ranging from 9.2–12.6% in cyt *b* and 8.1–14.1% in ND4, with additional support from partial allelic differentiation in phased nuclear markers, morphology, and osteology. Based on these results, we formally describe three new species, substantially expanding the known diversity of *Gloydius* along the southern Himalaya. To stabilise the nomenclature, we designate a lectotype for *Halys himalayanus* Günther. By integrating modern and historical data, this study demonstrates the lasting scientific relevance of natural history collections and emphasises the urgent need for conservation of these narrowly distributed and potentially regionally threatened mountain snakes.

## Introduction

The Himalaya-Tibet-Orogen is a vast mountain-building region extending more than 2400 kilometres across High Asia, encompassing the Himalaya, the Tibetan Plateau, and adjacent mountain systems such as the Hindu Kush, the Karakoram, and the Hengduan Shan. While this region is well known for its exceptional biodiversity and complex topography (Spicer et al. 2021, 2025; Xu et al. 2021), large areas remain insufficiently studied, particularly in terms of their genetic and taxonomic diversity. The extreme environmental gradients, from subtropical lowlands to some of the highest alpine zones on Earth, have led to significant species richness and endemism (Grenyer et al. 2006; Mittermeier et al. 2011). Large parts of the Himalaya-Tibet-Orogen, particularly in the west (including the western Himalaya and adjacent Hindu Kush), remain poorly explored due to logistical and political constraints. The region’s rugged terrain, limited road access, and complex socio-political situation, particularly in territories of Afghanistan and Pakistan (Jablonski et al. 2021a), continue to impede systematic fieldwork and specimen collection. At the same time, these mountainous regions foster geographic isolation, which can promote lineage divergence and substantial genetic differentiation as has been documented in connection with mountain uplift processes (Hoorn et al. 2013, 2018; Rahbek et al. 2019a, 2019b). This highlights their long-term importance for taxonomic research (e.g., Baig and Böhme 1996). Recent phylogenetic studies of reptiles and amphibians suggest that the western Himalaya and the Hindu Kush harbour deeply divergent lineages (Agarwal et al. 2014; Dufresnes et al. 2019; Gowande et al. 2021; Grismer et al. 2021, 2022; Hofmann et al. 2023, 2024a, 2024b). However, the evolutionary history of these lineages, and the taxonomic conclusions needed to interpret the underlying natural processes, remain poorly understood for many organisms in this part of Asia.

We address this gap in the genus *Gloydius* Hoge & Romano-Hoge, 1981, a group of mostly mountain-dwelling venomous pitvipers widely distributed across Eurasia, from the Caspian Sea to Japan (e.g., Gloyd and Conant 1990; Sindaco et al. 2013). Even though the genus *Gloydius* has recently undergone significant taxonomic revisions (see references below), populations from the Himalaya and Hindu Kush (see Nilson 1983) remain almost entirely unsampled in genetic studies. Over the past decade, eight new species have been described, mostly based on mitochondrial DNA, bringing the number of recognised species to 26 (e.g., Wagner et al. 2016b; Shi et al. 2021; Zhang et al. 2022; Kuttalam et al. 2022; Ren et al. 2025). These taxonomic advances, however, have largely focused on Central and Eastern Asia, leaving important gaps in our understanding of the *Gloydius* diversity in the Himalaya and the adjacent Hindu Kush.

The first molecular data on the genus *Gloydius* from the western Himalaya were presented by Shi et al. (2021) who included samples of *G. himalayanus* (Günther, 1864) from Himachal Pradesh, India (code HM 19.30; as shown in the phylogeny below). Their analysis placed this species as an ancestral lineage within the genus, with up to 16% mitochondrial genetic divergence (ND4 gene) from other congeners. This result suggests that the central and western Himalaya, along with the adjacent Hindu Kush, may be a key region for understanding the early diversification and evolutionary history of the genus. Kuttalam et al. (2022) further supported this view by describing a new species, *Gloydius chambensis* Kuttalam et al., 2022, from the Chamba Valley in India, based on a combination of mitochondrial and nuclear DNA as well as morphological data. Their study revealed two distinct lineages in the western Himalaya: one corresponding to *G. himalayanus* (although the type locality in "Garhwal, India" [Uttarakhand State] was represented only by morphological data) and another newly identified lineage restricted to the Chamba Valley in Himachal Pradesh State.

Considering these findings, we aimed to provide a comprehensive and integrative taxonomic revision of the *G. himalayanus* species complex. For that purpose, we combined mitochondrial and nuclear genetic data analyses with detailed morphological, osteological and distribution data to evaluate the taxonomic boundaries and phylogenetic relationships of *Gloydius* populations from the western and central Himalaya as well as the adjacent Hindu Kush. Our objective was to clarify species-level diversity within this group, identify previously unknown lineages, and contribute to a more complete and phylogenetically informed taxonomy of this widespread genus.

## Materials and methods

### Taxonomic sampling

To analyse genetic diversity within the *G. himalayanus* complex, we used 18 tissue samples representing populations from the western and central Himalaya as well as the Hindu Kush (Table 1). Seven samples were obtained during field surveys conducted between 2015 and 2023, whereas eleven were derived from voucher specimens preserved in museum collections, including material collected in the 19^th^ century. Although the overall sample size is modest due to limited field access (see Introduction), this dataset represents the most extensive geographic coverage of DNA material for this species complex to date. The inclusion of museum material was crucial for expanding both the geographic and taxonomic breadth of the dataset, particularly by integrating name-bearing type specimens. Tissue samples used for DNA analyses in this study originated from nine institutional collections. For details about institutional abbreviations, voucher numbers, sampling localities, and GenBank accession numbers see below and Table 1 and Suppl. material 1: table SS1.

**Table 1.** The material of the *Gloydius himalayanus* complex used for species delimitation based on the available genetic data obtained from the mid-19^th^ century to the present. The complete dataset of sequences is presented in Suppl. material 1: table SS1. *For "this study", it refers to the age and preservation condition of the material used for sequencing.

Species	Voucher number	Locality	N	E	Country	GenBank 12S/16S/cyt *b*/ND4/PRLR/NT3/C-mos	Source*
*Gloydius himalayanus*	BB87 (WII ADR 199)	Benog, Uttarakhand	30.47	78.01	India	-/-/PX290127/-/-/-/-	**This study** (collected in July 2019, ethanol-preserved)
	BB88	Kedarnath, Uttarakhand	~30.73	~79.07	India	-/-/PX290128/-/-/-/-	**This study** (collected in July 2018, ethanol-preserved)
	ZMB 2940	Kotgur [Kothgar]	31.52	77.78	India	PX280596/PX280560/ PX290129/PX290139/-/-/-	**This study** (collected between 1853–58, ethanol-preserved)
	BMNH 1946.1.19.64 – lectotype	Garhval [Garhwal Region]	~31.12	~78.34	India	PX280595/PX280559/-/-/-/-/-	**This study** (collected in 1854, ethanol-preserved)
	HM 19.30	Himachal Pradesh	-	-	India	MZ958982/ MZ958980/MZ959173/MZ959172/ -/-/-	Shi et al. (2021)
	17.v03	Himachal Pradesh	-	-	India	OP508270/OP508274/OP480166/OP407972/OP450866/OP450906/-	Kuttalam et al. (2022)
	18.30	Himachal Pradesh	-	-	India	-/-/-/-/OP450869/-/OP422596	Kuttalam et al. (2022)
	18.31	Himachal Pradesh	-	-	India	-/-/-/OP407973/-/-/OP422597	Kuttalam et al. (2022)
	19.30	Himachal Pradesh	-	-	India	-/-/-/-/OP450870/OP450907/OP422598	Kuttalam et al. (2022)
	-	-	-	-	India	MK559438 (mitogenome)	Simonov, unpublished
*Gloydius chambensis*	BMNH 1898.5.17.4	Chamba District, Himachal Pradesh	32.55	76.13	India	PX280591/PX280555/PX290130/PX290141/-/-/-	**This study** (collected ca. 1880, ethanol-preserved)
	BMNH 1898.5.17.5	Chamba District, Himachal Pradesh	32.55	76.13	India	PX280592/PX280556/PX290131/PX290140/-/-/-	**This study** (collected ca. 1880, ethanol-preserved)
	HARC-R 259 – holotype (18.13)	Bhanjraru, Chamba District, Himachal Pradesh	32.83	76.15	India	-/OP518269/-/OP407966/OP450868/OP422588/-	Kuttalam et al. (2022)
	NHMW 17079:1	Sirinapur [Srinagar]	34.09	74.79	India	PX280590/PX280554/-/-/-/-/-	**This study** (collected in 1866, ethanol-preserved)
	17.v20	Himachal Pradesh	-	-	India	OP508266/OP518268/ OP480162/OP407965/ OP450867/OP450901/ OP422592	Kuttalam et al. (2022)
	19.40	Himachal Pradesh	-	-	India	-/OP518272/OP480164/-/ OP450872/OP450903/ OP422594	Kuttalam et al. (2022)
	17.v13	Himachal Pradesh	-	-	India	**/**/**/**/**/ OP450900/OP422591	Kuttalam et al. (2022)
	19.50	Himachal Pradesh	-	-	India	OP508269/-/OP480165/OP407971/OP450874/OP450905/OP422595	Kuttalam et al. (2022)
	19.44	Himachal Pradesh	-	-	India	OP508268/OP518273/-/ OP407970/OP450873/ OP450904/OP422589	Kuttalam et al. (2022)
	19.38	Himachal Pradesh	-	-	India	**/**/**/**/OP450871/OP450902/ OP422593	Kuttalam et al. (2022)
*Gloydius hazarensis* sp. nov.	PMNH 4110	Sharan, Balakot District	34.70	73.44	Pakistan	PX280593/PX280557/PX290126/PX290138/-/PX290112/-	**This study** (collected in September 2017, ethanol-preserved)
	PMNH 4240	Mughalabad, Rawalpindi district	33.89	73.43	Pakistan	PX280594/PX280558/PX290118/-/-/-/PX290114	**This study** (collected in April 2020, ethanol-preserved)
*Gloydius hindukushensis* sp. nov.	NHMW 41993 – holotype	Kumrat Valley, Upper Dir District	35.56	72.19	Pakistan	PX280598/PX280561/PX290119/PX290133/PX290110/-/-	**This study** (collected in September 2020, ethanol-preserved)
	CUHC 10088 – paratype	Kumrat Valley, Upper Dir District	35.56	72.20	Pakistan	PX280597/PX280562/PX290120/PX290134/-/PX290113/-	**This study** (collected in September 2020, ethanol-preserved)
*Gloydius hindukushensis* sp. nov.	PMNH 5150	Lal Quilla, Lower Dir District	34.99	71.90	Pakistan	-/PX280563/PX290121/ PX290135/PX290111/-/-	**This study** (collected in April 2023, ethanol-preserved)
*Gloydius nepalensis* sp. nov.	ZMB 65611	Mustang District	28.76	83.69	Nepal	PX280601/PX280566/PX290122/-/-/-/-	**This study** (preserved in 1997, ethanol-preserved)
	ZMB 65612 – paratype	Syang, Mustang District	28.76	83.69	Nepal	PX280602/PX280567/PX290123/PX290136/PX290109/-/-	**This study** (collected in August 1997, ethanol-preserved)
	ZMB 65613 – holotype	Kalopani, Mustang District	28.61	83.59	Nepal	PX280603/PX280568/PX290124/PX290137/-/-/-	**This study** (collected in August 1997, ethanol-preserved)
	NME 070555	Narchyang, Mustang District	28.56	83.66	Nepal	PX280600/PX280564/PX290116/PX290132/-/-/-	**This study** (collected 1997, ethanol-preserved)
	NME R 0544/07 – paratype	Kermi, Humla District	29.21	82.34	Nepal	PX280604/-/PX290117/-/-/-/-	**This study** (collected in 2001, ethanol-preserved)
	RMNH.RENA. 20512 – paratype	Tal, Manang District	28.46	84.38	Nepal	PX280599/PX280565/-/-/-/-/-	**This study** (collected in 1981, ethanol-preserved)

**We treated specimens 17.v13, 19.38 and their sequences as likely misidentified or contaminated and excluded them from all tree analyses, because BLAST searches of the cytochrome *b* (OP480163) and PRLR (OP776641) sequences attributed to *Gloydius chambensis* by Kuttalam et al. (2022) recovered top matches to *Daboia russelii*.

### DNA extraction and amplification from modern samples

Total genomic DNA was extracted using the E.Z.N.A.® Tissue DNA Kit (Omega Bio-tek, Norcross, USA). The obtained DNA was amplified using polymerase chain reaction (PCR) in a total volume of 15 µl using the Red Taq 2X Master Mix 2 mM MgCl_2_. PCR products were purified using the ExoSAP-IT PCR Product Cleanup Reagent (Applied Biosystems, Foster City, CA; following manufacturer’s protocol) and DNA yield was controlled via gel electrophoresis. For the laboratory treatment of samples and primers, we followed the procedure of Kuttalam et al. (2022). Sequences of four mitochondrial (mtDNA) and three nuclear (nuDNA) genes were targeted: 12S rRNA, 16S rRNA, cytochrome *b* (cyt *b*), a fragment of NADH dehydrogenase subunit 4 (ND4) (including the flanking tRNAs Serine, Histidine, and part of Leucine), a gene coding for the neurotrophic factor neurotrophin-3 (NT3), the prolactin receptor (PRLR), and the oocyte maturation factor Mos (C-mos). Sequencing was performed by Macrogen Europe (Amsterdam; http://www.macrogen-europe.com) in the forward direction for mitochondrial DNA and in forward and reverse directions for nuclear DNA to compensate for sequencing errors and to detect heterozygote positions.

### Acquisition of molecular data from historical specimens

Seven historical specimens assigned to the *G. himalayanus* complex were minimally destructively sampled for muscle tissue by making a small incision in the thoracic region using sterilised scissors and tweezers. These included the voucher specimens BMNH 1946.1.19.64, BMNH 1953.1.1.69, and BMNH 1898.5.17.4–5 housed in the wet collection of the Natural History Museum in London; RMNH.RENA.20512 from the Naturalis Biodiversity Center in Leiden; NHMW 17079:1 from the Naturhistorisches Museum Wien; and ZMB 2940 from the Museum für Naturkunde Berlin.

Prior to genomic DNA extraction, all samples were weighed and incubated overnight (~18 hours) at 37 °C in a guanidine thiocyanate (GuSCN)-based extraction buffer. DNA was then extracted following the protocol of Rohland et al. (2004) and subsequent steps outlined by Straube et al. (2021), yielding 25 µl of extract per sample. DNA concentrations were quantified based on 1 µl of DNA extract with the Qubit dsDNA HS Assay Kit (Life Technologies, Carlsbad, CA) following the manufacturer’s instructions. Single-stranded libraries were prepared following the protocol of Gansauge et al. (2017), using up to 13 ng/µl input DNA per sample. All pre-qPCR procedures were conducted in a dedicated historical DNA laboratory at the University of Potsdam adhering to standards for working with museum specimens (see Fulton and Shapiro 2019). Extraction and library blanks were included to monitor potential contamination. Final library concentrations and fragment length distributions were assessed using a 2200 TapeStation (Agilent Technologies). Libraries were shotgun-sequenced on an Illumina NextSeq 500/550 platform at the University of Potsdam, targeting approximately five million 75 bp single-end reads per specimen (see Paijmans et al. 2018 for details).

Raw sequence data quality was assessed using FastQC (https://www.bioinformatics.babraham.ac.uk) before and after adapter trimming, duplicate removal, and filtering of reads shorter than 30 bp using cutadapt v. 1.12 (Martin 2011). The pre-processed reads were mapped to the complete mitochondrial genome of *G. himalayanus* (GenBank accession numbers MK559438.1/NC_068353.1) to extract the mitochondrial markers 12S, 16S, cyt *b*, and ND4. Mapping was performed using the Burrows-Wheeler Aligner (BWA; Li and Durbin 2009) and SAMtools (Li et al. 2009) and consensus sequences generated from the aligned reads. The mapping approach was unsuccessful for specimen BMNH 1953.1.1.69, preventing its incorporation in downstream analyses.

### Molecular phylogenetics

Chromatograms of newly generated sequences were checked visually in Geneious Prime v. 11.1.5 (Biomatters Ltd, Auckland) and heterozygotes at nuclear loci were identified based on secondary peak calling. All protein-coding gene fragments were translated into amino acids using DnaSP 6.00 (Rozas et al. 2017) to exclude frameshift mutations or premature stop codons. The dataset was subsequently corroborated by additional *Gloydius* sequence data available on GenBank, including all currently recognised species of the genus, and data of appropriate outgroups corresponding to the target loci. All available sequences attributed to *Gloydius chambensis* (19.38 and 17.v13) by Kuttalam et al. (2022) were excluded from tree analyses because BLAST searches recovered top matches to *Daboia russelii* for cytochrome *b* (OP480163) and PRLR (OP776641), suggesting likely misidentification or contamination. Individual alignments for each marker were generated using ClustalW in MEGA11 (Tamura et al. 2021) and manually checked for any inconsistencies and adjusted if necessary. All newly generated sequences were deposited in GenBank under the accession numbers listed in Table 1 and Suppl. material 1: table SS1.

We prepared two datasets for tree analyses: the first included four mitochondrial DNA genes (n = 60; 2693 bp: 12S 408 bp; 16S 482 bp; cyt *b* 1125 bp; ND4 678 bp), and a second dataset including four mtDNA and three nuclear genes (*n* = 60; 3882 bp: PRLR 208 bp, NT3 473 bp, C-mos 508 bp). Based on this locus-partitioned dataset, we inferred a maximum likelihood (ML) phylogeny using IQ-TREE2 v. 2.1.2 (Minh et al. 2020), and a Bayesian inference (BI) tree using MrBayes v. 3.2.7 (Ronquist et al. 2012). For each marker, the best-suited substitution model was inferred using PartitionFinder (Lanfear et al. 2012).

For BI analysis we performed four runs, with four chains and 10 million generations each, sampling trees every 1000^th^ generation, and discarding the first 25% of each run as burn-in. Chain convergence was monitored with Tracer v. 1.7.1 (Rambaut et al. 2018). A majority-rule consensus tree was drawn from the post-burn-in samples and posterior probabilities (PP) were calculated as the frequency of samples recovering any clade. Branch support was assessed with 1000 replicates using the Shimodaira-Hasegawa-like approximate likelihood ratio test (SH-aLRT; Guindon et al. 2010) and the Ultrafast bootstrap approximation algorithm (UFBoot; Minh et al. 2013). In the ML analysis with 1000 pseudoreplicates we considered nodes with bootstrap values of ≥ 95 as strongly supported, and those with values > 90 as well supported. Nodes with PP values > 0.95 were considered strongly supported.

### Haplotype (allele) networks

Alignments of the protein-coding nuclear genes C-mos (508 bp), NT3 (473 bp), and PRLR (208 bp) were analysed separately from the mitochondrial markers mentioned above to obtain evidence from unlinked loci (mitochondrial versus nuclear) for the differentiation of lineages. For this purpose, alleles were inferred from heterozygous single-nucleotide polymorphisms using the PHASE algorithm (Stephens et al. 2001) as implemented in Hapsolutely v. 0.2.3 (Vences et al. 2024), using a phase (-p) and allele (-q) threshold of 0.7 with 1000 MCMC iterations. Subsequently, allele genealogies were reconstructed based on the TCS algorithm (Templeton et al. 1992).

### Genetic distances

Uncorrected pairwise distances were calculated in MEGA X (Kumar et al. 2018), based on the mtDNA alignment used for the inference of the multi-gene tree. For this purpose, the alignment was separated by loci, sequences with ≥ 50% missing data were excluded, and each of the separated marker datasets was complemented with additional sequence data from NCBI to ensure that all *Gloydius* species are represented in the p-distance matrix. The number of base differences per site from averaging over all sequence pairs between species was estimated. The analysis involved 40 sequences for cyt *b* and 43 for ND4. Included codon positions were 1^st^+2^nd^+3^rd^+non-coding for both markers. All ambiguous positions were removed for each sequence pair (pairwise deletion option). This led to a final dataset counting a total of 1125 positions for the cyt *b* dataset and 678 positions for ND4.

### External morphology, meristics, and colouration

We examined 94 preserved museum specimens of the genus *Gloydius* covering the geographic range of the Hindu Kush and Himalaya from its westernmost voucher in Afghanistan to west-central Nepal including its easternmost voucher specimen. The material also includes the four relevant type specimens of *Gloydius* previously described from the Himalaya and the Hindu Kush (see Suppl. material 1).

Voucher specimens were studied or referenced from the following museums and institutional collections: **AMNH** (American Museum of Natural History, New York, USA), **BEIC** (British East India Company Museum, later India Museum, London, UK), **BMNH** (Natural History Museum, London, UK, formerly British Museum (Nat. Hist.), the material cited in this paper is registered under this acronym), **BNHS** (Bombay Natural History Society, Mumbai, India), **CUHC** (Comenius University Herpetological Collection, Bratislava, Slovakia), **HARC** (High Altitude Research Centre, Solan, Himachal Pradesh, India), **KU** (University of Kansas Biodiversity Institute, Lawrence, USA), **MCZ** (Museum of Comparative Zoology, Harvard University, Cambridge, USA), **MHNG** (Muséum d’Histoire naturelle, Geneva, Switzerland), **NHMD** (Natural History Museum of Denmark, University of Copenhagen, formerly **ZMUC** Zoologisk Museum Universitet Copenhagen), **NHMK** (Natural History Museum Kathmandu, Nepal), **MNHN** (Muséum national d’Histoire naturelle, Paris, France), **NME** (Naturkundemuseum Erfurt, Germany), **NHMW** (formerly NMW, Naturhistorisches Museum Wien, Vienna, Austria), **PMNH** (Pakistan Museum of Natural History, Islamabad), **RMNH.RENA** (Naturalis Biodiversity Center, Leiden, The Netherlands), **SMNS** (Stuttgart State Museum of Natural History, Germany), **ZMA.RENA** (former collection of Zoological Museum Amsterdam, now housed in RMNH Naturalis Biodiversity Center, Leiden, The Netherlands), **SMF** (Senckenberg Natural History Frankfurt, Frankfurt am Main, Germany), **UF** (University of Florida, Florida Museum of Natural History, Gainesville, USA), **UMMZ** (University of Michigan Museum of Zoology, Ann Arbor, USA), **USNM** (Smithsonian Institution, National Museum of Natural History (NMNH), Washington, USA), **WII** (Wildlife Institute of India, Dehradun), **ZFMK** (Museum Koenig, Leibniz Institute for the Analysis of Biodiversity Change (LIB), Bonn, Germany), **ZMB** (Museum für Naturkunde, Leibniz-Institut für Evolutions- und Biodiversitätsforschung, Berlin, Germany, formerly Zoologisches Museum Berlin), **ZMH** (Museum of Nature Hamburg formerly Zoologisches Museum Hamburg, Germany), **ZSM** (Zoologische Staatssammlung München, Munich, Germany).

The measurement and count scheme included the following morphological characters: **SVL** – snout-vent length; **TaL** – tail length; **TL** – total length (SVL+TaL); **TaL/TL** – ratio tail length/total length; **HW** – head width, measured at its widest lateral extension; **HL1** – head length, measured from tip of snout to posterior edge of the parietal scales; **HL2** – head length, measured from tip of snout to posterior edge of the mandible; **RW** – rostral width; **FL** – frontal length; **FW** – frontal width; **PrefL** – prefrontal length; **PrefW** – prefrontal width; **INL** – internasal length; **INW** – internasal width; **FW.L**. – ratio frontal width/frontal length; **PrefW.L**. – ratio prefrontal width/prefrontal length; **INW.L**. – ratio internasal width/internasal length; **DSR** – dorsal scale rows: numbers are given at one head length behind head (**dorsalia 1**), at midbody (taken halfway along the total number of ventral scales; **dorsalia 2**), and at one head length before vent, respectively (**dorsalia 3**); **PreVen** – number of preventrals; **Ven** – number of ventrals; **SubC** – number of subcaudals; **TS** – total number of ventral and subcaudal scales including preventrals and terminal scale; **CpP** – dorsal head scales circum posterior pileus, counted from (including) upper **PoO left** head side to upper **PoO right** side; **PrO** – number of preoculars; **PoO** – number of postoculars; **T** – number of temporal scales in first and second horizontal row; **SupL** – number of supralabials; **SubL** – number of sublabials; **SubL contacting first pair submaxillars** – number of sublabials contacting the first pair of submaxillars.

Total length, snout-vent length, and tail length were measured with thread and a ruler to the nearest 1 mm. Other dimensions were recorded with dial callipers, to the nearest 0.1 mm. Length and width of head scales were measured at the longest and widest points of the respective scale(s). The number of ventral scales was counted according to Dowling (1951a). Incomplete (half) ventral scales were counted as one. Dorsal scale row reduction formulae were based on Dowling (1951b). The terminal scale is not included in the subcaudal count. Values for symmetric characters are provided in left/right order. Circum-pileus scales are in contact with the posterior scales of the pileus and were counted from left to right (both included) upper postocular. Sex was determined by the presence or absence of hemipenes through a subcaudal incision on the tail base. For the description of the colours, we use the names and colour codes (given in brackets) according to Köhler (2012).

### Micro-computed tomography and measured osteological characters

Micro-Computed Tomography (micro-CT) scans were performed using a Phoenix nanotom X-ray|s (Waygate Technologies, Wunstorf, Germany; Equipment identification ID SCR_022582) and a Comet YXLON FF85 (Comet Yxlon GmbH, Hamburg, Germany; Equipment identification ID SCR_020917) at the Museum für Naturkunde Berlin (Laboratory identification number SCR_022585). These non-invasive scans were conducted to study the cranial morphology of *Gloydius* specimens. Scanning parameters ranged from 90 to 100 kV and 100 or 200 μA, producing between 1440 and 2000 projections at 750 ms per scan. Voltage and current settings were adjusted based on the size of each specimen. The effective voxel size (i.e., the resolution in three-dimensional space) ranged from 14.16 to 71.16 μm. Cone beam reconstruction was carried out using either datos|x 2.2 reconstruction software (Waygate Technologies, Wunstorf, Germany) or Nexus software (Comet Yxlon GmbH, Hamburg, Germany), and the data were visualised in VG Studio Max 3.5 (Volume Graphics GmbH, Heidelberg, Germany).

The osteological characters and skull elements recorded and measured for this study are presented in Suppl. material 1: figs S1, S2. The description of dentition partly follows Ernst (1982a, 1982b). Used abbreviations: **DL.CpbL.perc** – Percentage that the dental spans the compound bone; **FL.HL** – Ratio of fang length to osteological head length; **OL.FL** – Ratio of discharge orifice length to fang length; **PteL.PteW** – Ratio of pterygoid length to pterygoid width; **PteS.PteL.perc** – Percentage of toothless part length of total pterygoid length; **SAL.CpbL** – Ratio of splenial–angular length to compound bone length; **Teeth Dent** – Number of dentary teeth; **Teeth Pal** – Number of palatine teeth; **Teeth Pt** – Number of pterygoid teeth.

### Body and osteological data analyses

Morphological data included both meristic and mensural characters. Individuals with incomplete or missing values were excluded prior to analyses using listwise deletion (R function *na.omit*). For mensural characters, only adult specimens were considered, while for meristic data, juveniles were included as scale patterns form early in embryogenesis (Alibardi 1997) and remain unchanged during ontogeny (Chang et al. 2009). Statistical analyses were performed in R v. 4.5.0 (R Core Team 2025) using the packages *dplyr*, *car*, *ggplot2*, *ggrepel*, *ggsignif*, *emmeans*, *FSA*, *multcomp*, and *openxlsx*.

Males and females were analysed jointly, as preliminary t-tests within species did not reveal substantial sexual dimorphism in morphometric or meristic characters. Absolute traits and ratio traits were extracted from raw measurements, with all absolute traits and snout–vent length (SVL) log-transformed. For absolute traits, analyses of covariance (ANCOVA) were conducted with log-SVL as a covariate and species as a fixed factor. For ratio traits, assumptions of normality (Shapiro–Wilk test) and homogeneity of variance (Levene’s test) were assessed; traits meeting these assumptions were analysed using one-way ANOVA, otherwise the non-parametric Kruskal–Wallis test was applied.

Principal component analysis (PCA) was conducted on log-transformed, size-corrected morphometric variables and ratio traits using the *prcomp* function. Traits were centred and scaled prior to analysis to standardise measurement units. Species-specific distributions were visualised with boxplots.

Raw meristic data were not subjected to any a priori transformations. Meristic traits, being discrete counts, were analysed individually. Depending on the distribution of each trait, overall differences among species were assessed using Kruskal–Wallis rank-sum tests. For traits showing significant overall differences, pairwise post hoc comparisons were performed using Dunn’s test with Bonferroni correction for multiple testing. Multivariate variation among species was explored using PCA. Trait distributions were visualised using boxplots.

Osteological ratios and tooth counts were analysed without size correction, as these traits are considered size-independent. Ratios included FL.HL, OL.FL, SAL.CpbL, DL.CpbL.perc, PteL.PteW, and PteS.PteL.perc, while count traits comprised the number of palatine, pterygoid, and dentary teeth. Given the small sample size, for ratio traits, overall differences were tested using one-way ANOVA followed by Tukey’s HSD when assumptions of normality and homogeneity were met; otherwise, Kruskal–Wallis tests followed by Dunn’s tests with Holm adjustment were used. For count data, overall differences were tested using Kruskal–Wallis tests followed by Dunn’s tests with Holm adjustment. Because preserved material was limited, only 21 micro-CT scans were available: two specimens of *G. chambensis* (1 male, 1 female), five specimens of *G. himalayanus* (4 males, 1 female), one specimen of the Hindu Kush lineage (female), five from the Hazara lineage (1 male, 4 females) and eight from the central Himalayan lineage (5 males, 3 females). Given these small sample sizes, both sexes were analysed together.

## Results

### Phylogenetic relationships within the *G. himalayanus* complex

**Figure 1.**
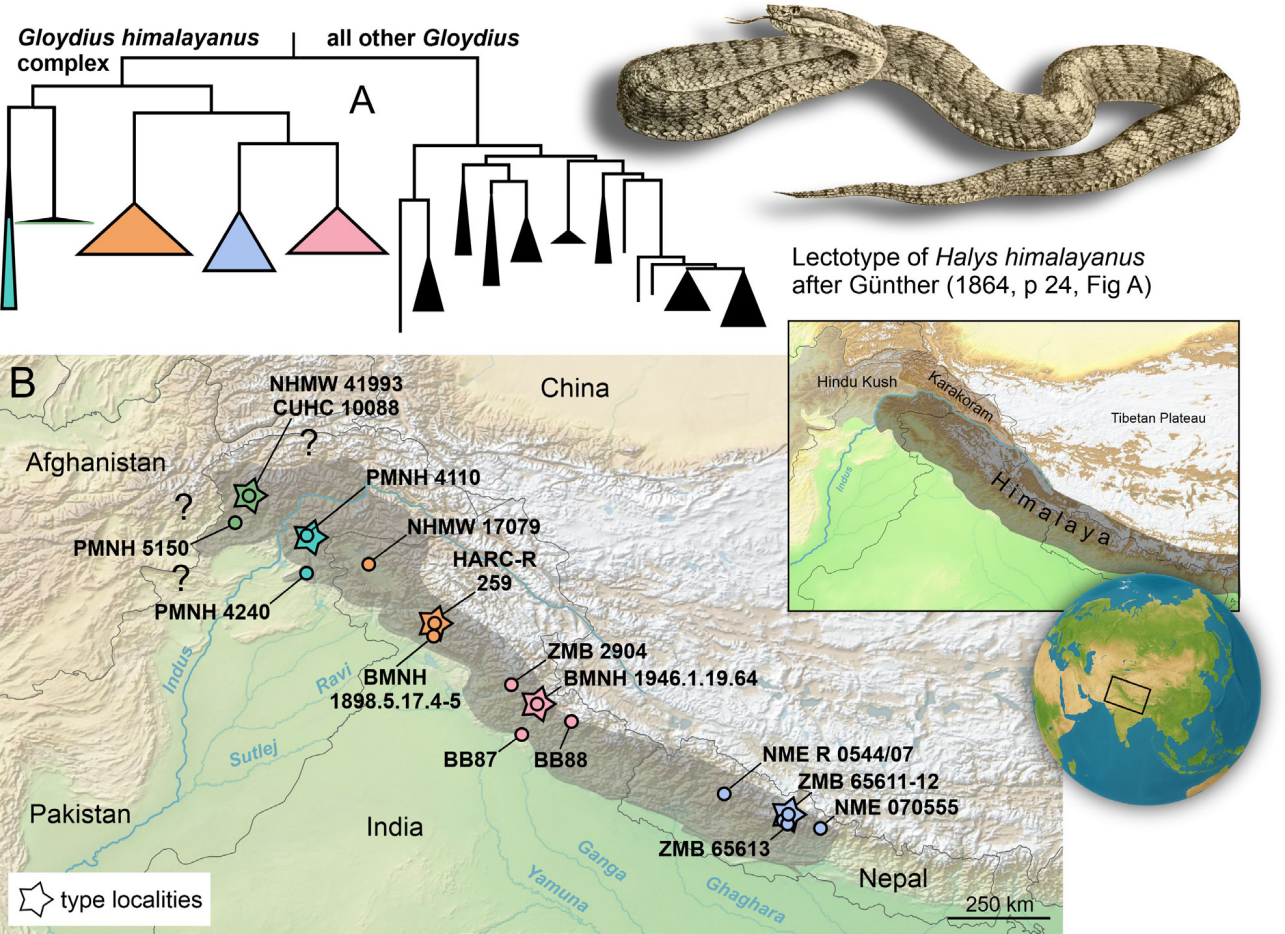
**A**. Phylogenetic placement of the *G. himalayanus* complex within the genus *Gloydius* (for details see Fig. 2A); **B**. Geographic distribution (dark shading) of recognised lineages within the *G. himalayanus* complex, along with the sampling localities of genetically investigated specimens (see Table 1, Suppl. material 1: table SS1). The position of the locality for the lectotype BMNH 1946.1.19.64 is only for map purposes (see remarks on the lectotype in the species account). Due to incomplete locality data in Kuttalam et al. (2022) (see Fig. 2A), some sample locations are not depicted in panel (**B**); an inset shows the approximate extent of the major mountain ranges in the region, following Spicer et al. (2021, 2025). Question marks indicate uncertain or potential occurrences in eastern Afghanistan. The map was generated using QGIS 3.28 (https://qgis.org/) with the WGS 84 coordinate reference system. Sketch shows a syntype of *Halys himalayanus*, taken from Günther (1864). This individual corresponds to the specimen BMNH 1946.1.19.64, which is designated here as the lectotype for *Gloydius himalayanus*.

**Figure 2.**
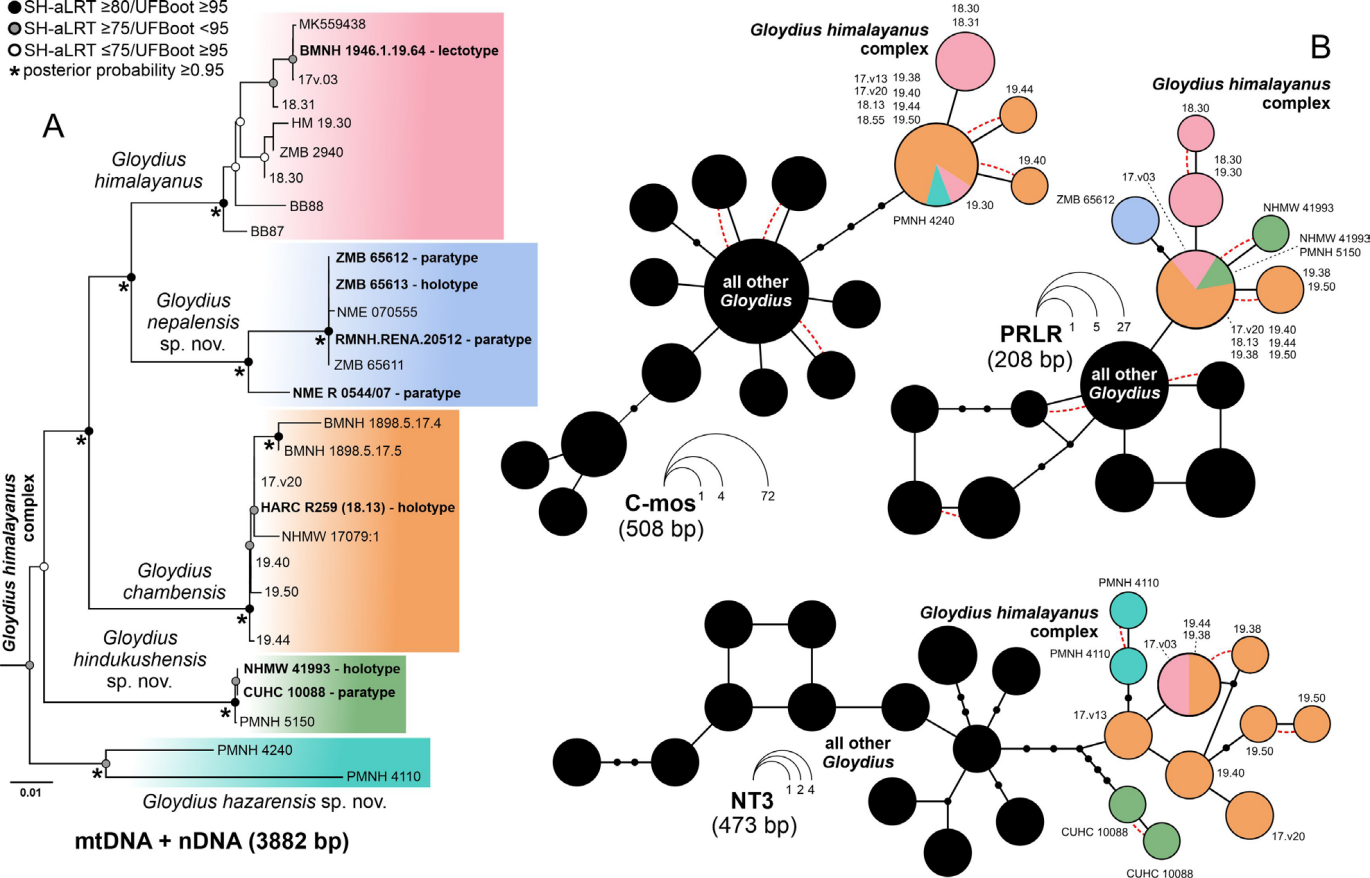
DNA-based relationships in the *Gloydius himalayanus* complex. **A**. Maximum-likelihood phylogeny inferred from concatenated mitochondrial and nuclear markers (3882 bp) showing relationships within species of the *G. himalayanus* complex. SH-aLRT, ultrafast bootstrap (UFBoot), and posterior probability (PP) values indicate nodal support. Coloured boxes correspond to the clades recognised in this study (Fig. 1); **B**. Ninety-five percent statistical parsimony allele networks for three nuclear loci (C-mos, PRLR, NT3) showing relationships among phased alleles of the *G. himalayanus* complex and other *Gloydius* species. Each circle represents one unique allele; circle size is proportional to its frequency, and colours indicate species assignment. Small black circles correspond to inferred unsampled alleles, and red dashed lines indicate mutational connections between alleles of the *G. himalayanus* complex and those of other *Gloydius*.

Phylogenetic analyses of the concatenated mitochondrial and nuclear DNA dataset (3882 bp) recovered the *Gloydius himalayanus* complex as a strongly supported monophyletic group (SH-aLRT ≥ 80; UFBoot ≥ 95; PP ≥ 0.95), distributed from the south-eastern foothills of the Hindu Kush and the northwestern Himalayan borderlands of Pakistan through northwestern India to central Nepal (Figs 1A, 1B, 2A). Within this complex, five well-supported monophyletic lineages were consistently identified across analyses (Figs 1, 2, Suppl. material 1: fig. S3), corresponding to *Gloydius chambensis*, *G. himalayanus* sensu stricto, and three new species described below from (i) the Hindu Kush region of Pakistan, (ii) the Hazara region of Pakistan, and (iii) the western and central Himalaya of Nepal. Notably, the Himalayan clades (*G. himalayanus* s.s. and the Nepalese lineage) and the Hazara lineage show deeper within-lineage substructure than *G. chambensis* and the Hindu Kush lineage, although this pattern may partly reflect uneven sampling (Fig. 1B).

In the concatenated ML analysis, the Hazara lineage was recovered as the earliest-diverged lineage within the *G. himalayanus* complex, potentially separated from the Hindu Kush lineage by the Upper Indus River Valley. The Hindu Kush populations formed a distinct lineage, currently known only from the south-eastern to eastern foothills of the Hindu Kush in northwestern Pakistan. *Gloydius chambensis* was represented by individuals from the Chamba region in the northwestern Himalaya of India and, unexpectedly, by a specimen from the Kashmir region (Srinagar; NHMW 17079:1). It was recovered as sister (SH-aLRT ≥ 75; UFBoot < 95) to a clade comprising the two lineages occurring east of its range. The Nepalese lineage (western and central Nepal) occurs east of the Karnali River Valley and was recovered as sister to *G. himalayanus* sensu stricto. *Gloydius himalayanus* s.s. includes populations from northwestern India and may occur in close geographic proximity to *G. chambensis* (e.g., along the Ravi River Valley and elsewhere in Himachal Pradesh), although sympatry remains unconfirmed.

Phylogenetic inference based solely on mitochondrial DNA (2693 bp; Suppl. material 1: fig. S4) yielded a largely congruent topology and supported the same five lineages. Minor differences were observed in nodal support, particularly for the relationship between the Hindu Kush and Hazara lineages, but the overall topology of the complex remained stable and well supported across datasets.

Bayesian inference (BI) analyses of both the concatenated mitochondrial+nuclear dataset (3882 bp) and the mitochondrial-only dataset (2693 bp) (Suppl. material 1: figs S5, S6) also recovered the *G. himalayanus* complex as monophyletic and broadly consistent with the ML topologies. The concatenated BI tree (Fig. 2A, Suppl. material 1: fig. S5) recovered all five major lineages with high posterior support (PP ≥ 0.95), although support for the sister relationship between the Hindu Kush and Hazara lineages was low. The mitochondrial-only BI tree (Suppl. material 1: fig. S6) supported the same overall topology and recovered strong support for the Hindu Kush–Hazara sister relationship.

The geographic distribution of the major lineages closely corresponds to their phylogenetic structure (Fig. 1). However, large areas (e.g., eastern Afghanistan) remain poorly sampled or entirely unsampled, and additional field and museum-based sampling will be required to refine the inferred range limits and relationships within the complex.

### Nuclear diversity within the *G. himalayanus* complex

Allele networks inferred for the three protein-coding nuclear markers C-mos, NT3, and PRLR are depicted in Fig. 2B, with colours of each taxon corresponding to lineages revealed in Fig. 2A.

The highest diversity was observed for NT3, with 11 distinct alleles unique to members of the *G. himalayanus* complex. Within this network, the Hazara lineage (PMNH 4110) and the Hindu Kush lineage (CUHC 10088) are each represented by two unique alleles, whereas *G. himalayanus* shares one allele with *G. chambensis*, which in turn possesses six additional unique alleles (Fig. 2B).

The PRLR gene revealed six distinct alleles within the *G. himalayanus* complex. The lineage from the central Himalaya in Nepal (ZMB 65612) is represented by a unique allele separated by two mutational steps from the shared allele. The Hindu Kush lineage (NHMW 41993) is represented by two alleles, one of which is private and the other shared with *G. himalayanus* and *G. chambensis*. *Gloydius chambensis* exhibits one species-specific allele in addition to the shared allele, whereas *G. himalayanus* comprises three alleles, two of which are species-specific (Fig. 2B).

The nuclear marker C-mos showed the lowest allelic diversity within the *G. himalayanus* complex (sequences representing lineages from the Hindu Kush and central Himalaya were not available), with four distinct alleles detected among representatives of *G. himalayanus*, *G. chambensis*, and the Hazara lineage (PMNH 4240). One allele is shared by all three taxa, whereas *G. himalayanus* possesses one private allele and *G. chambensis* two private alleles. Allelic differentiation within the complex is low, with the alleles separated by a single mutational step (Fig. 2B).

The available nuclear data reveal partial allelic differentiation among mtDNA-defined lineages within the *G. himalayanus* complex and do not contradict the mitochondrial pattern. However, several sequences reported by Kuttalam et al. (2022) appear problematic and were excluded from our interpretation because they do not cluster with *Gloydius* (see Materials and methods). Consequently, previously published nuclear data attributed to *G. himalayanus* and *G. chambensis* should be treated with caution.

### Genetic distances

Interspecific genetic distances in protein-coding mtDNA within *Gloydius* reached up to 15.4% for the cyt *b* gene and 14.6% for ND4, values consistent with levels of mitochondrial divergence typically observed among described species (Suppl. material 1: table SS2). However, interspecific distances at the lower end of the range (~0.4–3%) overlap with levels commonly associated with intraspecific variation in many vertebrates, including reptiles (see, e.g., Wüster et al. 2024). Such shallow divergence may indicate conspecificity rather than species-level separation and warrants careful re-evaluation using complementary data sources (see discussion below).

Within the *G. himalayanus* complex, the five main lineages showed pairwise distances ranging from 9.2–12.6% for cyt *b* and 8.1–14.1% for ND4, which fall well within the interspecific range (Table 2, Suppl. material 1: table SS2). In contrast, within-lineage variation was generally low, ranging from 0.11–1.10% for cyt *b* and up to 1.2% for ND4, except in *G. himalayanus*, which showed within-lineage divergence of up to 4.7% (cyt *b*) and 5.1% (ND4), suggesting the presence of cryptic diversity or historical population fragmentation.

**Table 2.** Uncorrected *p* distances (%) for cytochrome *b* (lower diagonal) and ND4 (upper diagonal) genes in the *Gloydius himalayanus* complex. The highest values are highlighted in bold. For data from other species of the genus *Gloydius* see Suppl. material 1: table SS2.

*p* distances (%)	*G. himalayanus*	*G. chambensis*	*G. hazarensis* sp. nov.	*G. hindukushensis* sp. nov.	*G. nepalensis* sp. nov.
*G. himalayanus*		8.88	**14.06**	10.63	9.47
*G. chambensis*	10.88		11.99	11.02	9.94
*G. hazarensis* sp. nov.	11.17	11.66		12.50	11.93
*G. hindukushensis* sp. nov.	10.56	11.12	10.36		8.08
*G. nepalensis* sp. nov.	9.23	10.82	**12.61**	12.05	

### Morphological differences in the *Gloydius himalayanus* complex

**Figure 3.**
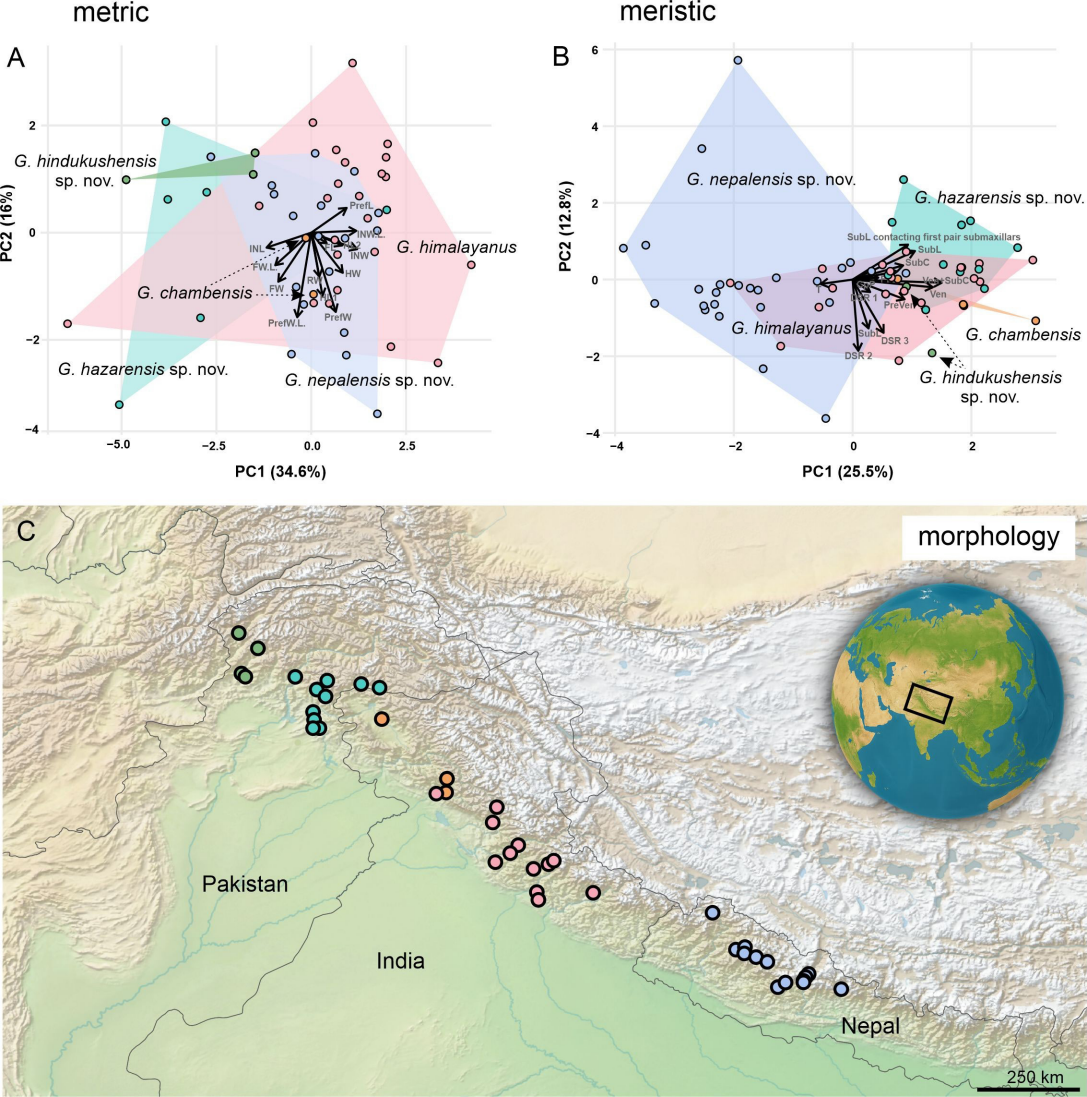
Principal Component Analyses (PCA) illustrating metric **A**. and meristic **B**. differentiation among species of the *Gloydius himalayanus* complex. Scatterplots show the first two principal components (PC1 and PC2) derived from scaled, log-transformed, size-corrected metric measurements (A) and scaled meristic counts (B). Points represent individual specimens, colour-coded by species, and convex hulls (shaded polygons) outline the spread of each taxon in morphospace. Full arrows indicate the loading vectors of the most influential variables, representing the contribution of each trait to the PCA axes. Dashed lines/arrows are included only as visual guides to displaced labels and have no additional analytical meaning. Axis labels include the percentage of variance explained by each principal component; **C**. Geographic distribution of sampled populations across Pakistan, India, and Nepal, colour-coded according to Figs 1, 2. The inset shows the regional context within Central and South Asia. The map was produced in QGIS 3.28.

Morphological variation among the genetically defined lineages of the *Gloydius himalayanus* complex (*G. chambensis*, the Hazara lineage, *G. himalayanus*, the Hindu Kush lineage, and the lineage from the central Himalaya of western and central Nepal) was assessed using PCA of metric and meristic characters. The resulting groupings corresponded well to the phylogenetically inferred lineages (Figs 1, 2), and the PCA plots (Fig. 3) reveal patterns of morphological differentiation of the complex, including among the three newly described species.

In the metric dataset (Fig. 3A), the first two principal components accounted for 34.6% and 16.0% of the total variance, respectively. *Gloydius himalayanus*, *G. chambensis* and three newly detected lineages showed partial separation, although with certain overlap among lineages. The Hazara and Hindu Kush lineages occupied relatively peripheral positions along PC1, whereas the central Himalaya lineage and *G. himalayanus* partly overlapped in the central/right part of the morphospace. The most influential loadings were associated mainly with head measurements and proportions, particularly prefrontal and internasal dimensions and ratios, together with head length and head width variables.

In the meristic dataset (Fig. 3B), the first two principal components explained 25.5% and 12.8% of the variance, respectively. Despite partial overlap, the species likewise formed discernible clusters, with the lineages from the central Himalaya in Nepal and the Hazara region showing the most distinct meristic profiles. The variables contributing the most to the differentiation included labial counts and dorsal scale-row characters.

**Figure 4.**
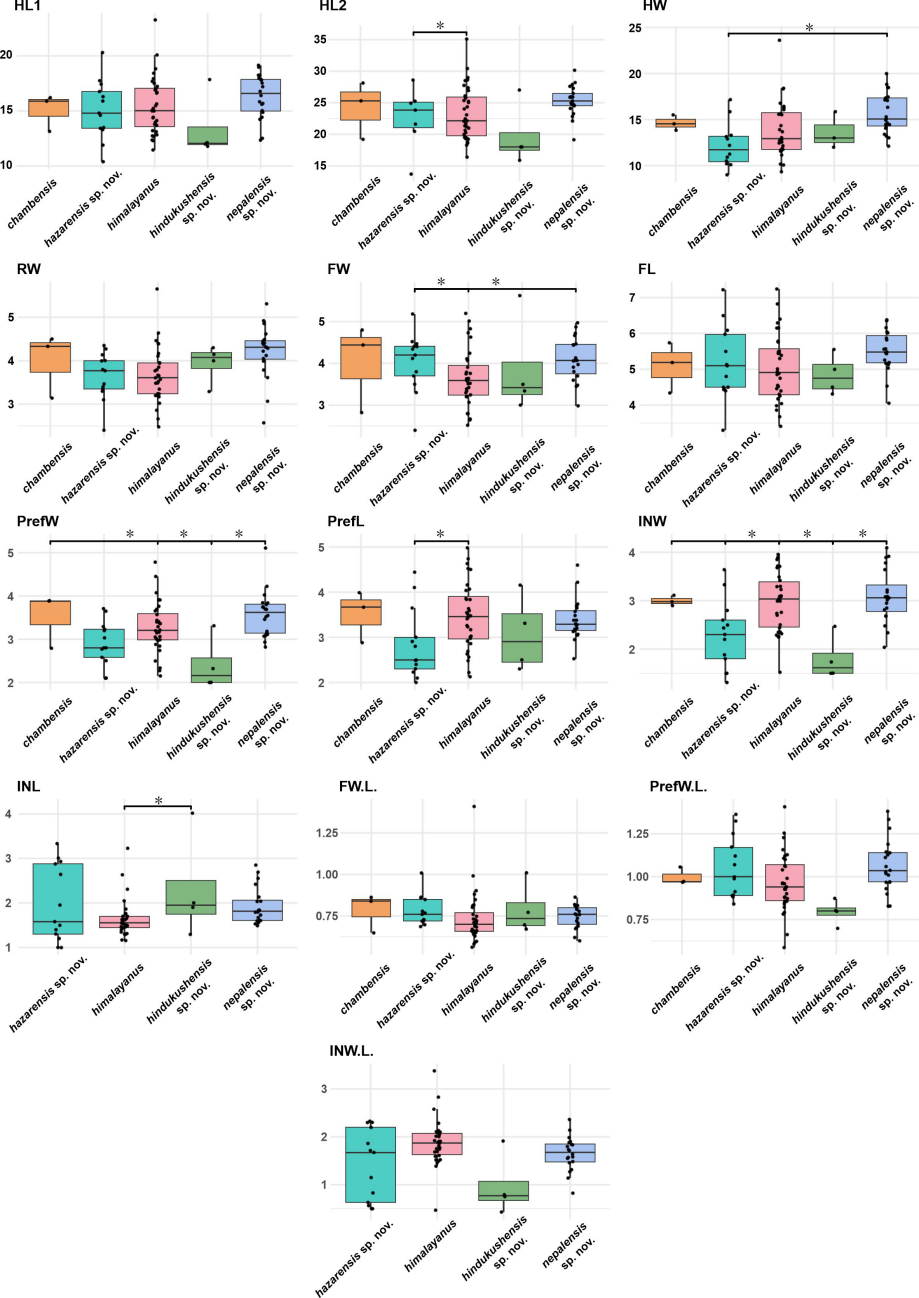
Boxplots showing variation in metric traits (see Materials and methods) among the *Gloydius himalayanus* complex. Boxes indicate the interquartile range (IQR) with median values shown as horizontal lines; whiskers extend to 1.5 × IQR. Individual data points are overlaid and jittered (width = 0.2) along the x-axis to avoid overplotting. Statistically significant differences between species (*p* ≤ 0.05) are marked by asterisks. Y-axis values are log-transformed, size-corrected units. Boxplots are colour-coded according to genetically defined lineages (Figs 1, 2).

**Figure 5.**
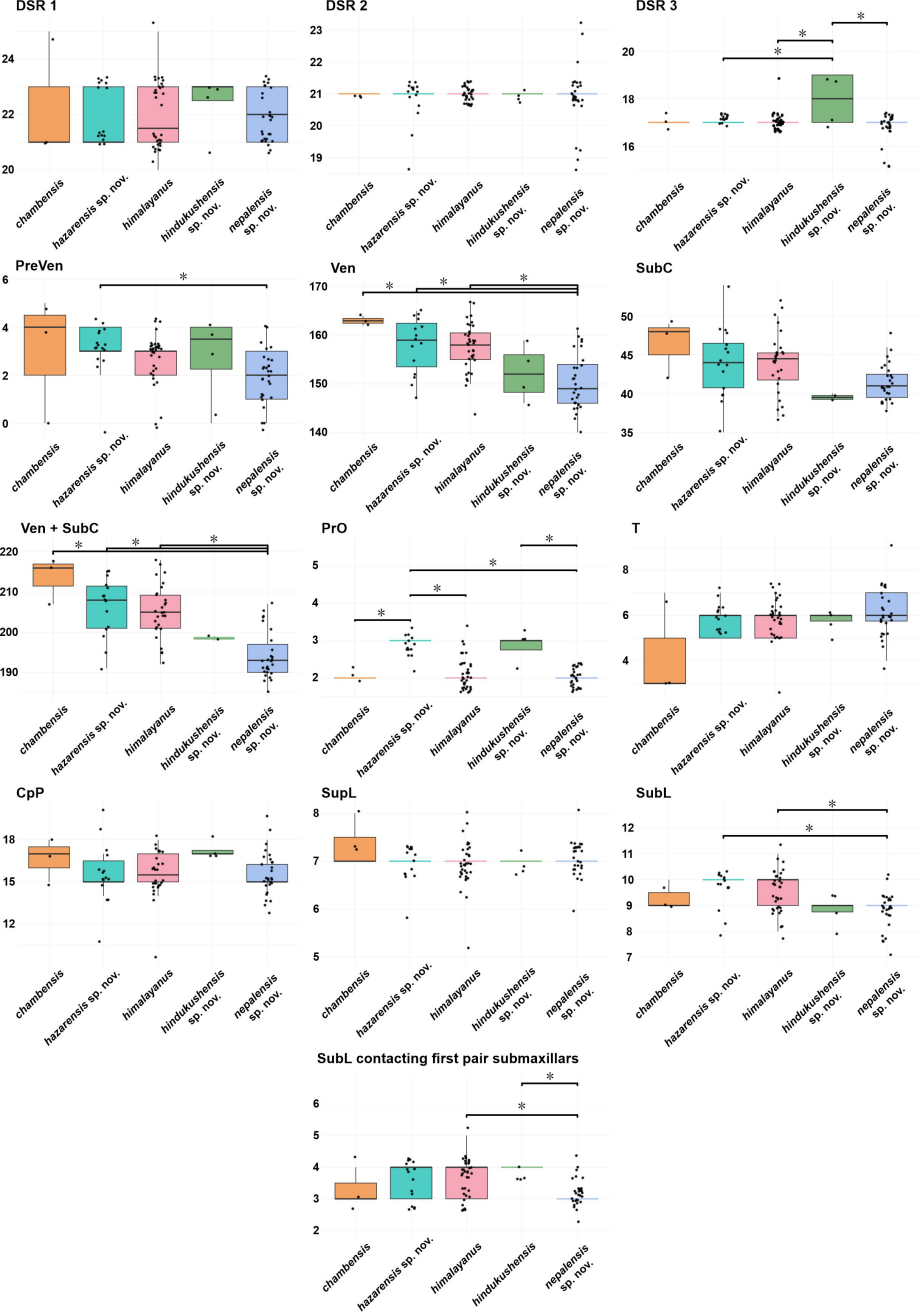
Boxplots showing variation in selected meristic characters (see Materials and methods) among the *Gloydius himalayanus* complex. Each box represents the distribution of values for a given meristic trait across species. Boxes show the interquartile range (IQR) with the median values indicated by a horizontal line; whiskers extend to 1.5 × IQR. Individual data points are overlaid and jittered along the x-axis to visualise all observations. Statistically significant differences between species are marked by asterisks (*p* ≤ 0.05). Y-axis values represent integer counts. Boxplots are colour-coded according to genetically defined lineages (Figs 1, 2).

Boxplot analyses of head measurements and of meristic characters (Figs 4, 5) indicated morphological differentiation among the five lineages of the *G. himalayanus* complex. These results are consistent with the PCA and support partial morphological distinctiveness among lineages, although most metric head characters showed some overlap (Fig. 4). HL1 showed no significant pairwise differences among lineages, whereas HL2 differed significantly between the Hazara lineage and *G. himalayanus* (p < 0.05). Head width was greatest in the central Himalayan lineage from Nepal and differed significantly from the Hazara lineage (p < 0.05). *Gloydius chambensis* and *G. himalayanus* occupied intermediate positions with partial overlap. Frontal and prefrontal widths (FW, PrefW) also varied among lineages, with broader cranial plates in *G. chambensis* and the central Himalayan lineage and narrower values in the Hazara and Hindu Kush lineages (*p* < 0.05; but see FW). Differences in the internasal region were moderate: INW tended to be greater in the central Himalayan lineages, including *G. himalayanus*, whereas INL was greater in the Hindu Kush lineage than in *G. himalayanus* (*p* < 0.05). The Hazara lineage displayed intermediate and variable values, overlapping with both *G. himalayanus* and the Hindu Kush lineage. Shape indices (FW.L., PrefW.L., INW.L.) supported these trends, indicating broader anterior cranial elements in the central Himalayan lineage and the Hazara lineage, contrasted with the more elongate proportions of the Hindu Kush lineage (Fig. 4).

Number of ventrals (Ven) and combined ventrals plus subcaudals (Ven + SubC) showed the clearest inter-lineage differences (Fig. 5), with several pairwise comparisons being statistically significant (*p* < 0.05). SubC tended to be higher in *G. chambensis* and the Hazara lineage, and lower in the Hindu Kush lineage and the lineage from the central Himalaya in Nepal, although with partial overlap with *G. himalayanus*. Ven were highest in *G. chambensis* and tended to be higher in the Hazara lineage and *G. himalayanus*, whereas lower values characterised the Hindu Kush lineage and especially the lineage from central Himalaya in Nepal (*p* < 0.05). Preventrals (PreVen) differed only weakly, with the plotted pairwise comparisons supporting a difference between the Hazara and central Himalayan lineages. Dorsal scale rows DSR 1 and DSR 2 were largely similar across taxa, whereas DSR 3 was elevated in the Hindu Kush lineage relative to several other lineages. Differences were also evident in head scalation: the number of preoculars (PrO) was lowest in *G. chambensis* and tended to be high in the Hazara and Hindu Kush lineages, while the number of sublabials (SubL) was higher in the Hazara and *G. himalayanus* than in the central Himalaya lineage from Nepal (*p* < 0.05). The number of SubL contacting first pair of submaxillars was also higher in *G. himalayanus* and the Hindu Kush lineage than in the lineage from the central Himalaya in Nepal. By contrast, variation in the number of temporal scales in first and second horizontal row (T), the number of dorsal head scales circum posterior pileus (CpP), and the number of supralabials (SupL) was moderate and broadly overlapping (Fig. 5).

**Figure 6.**
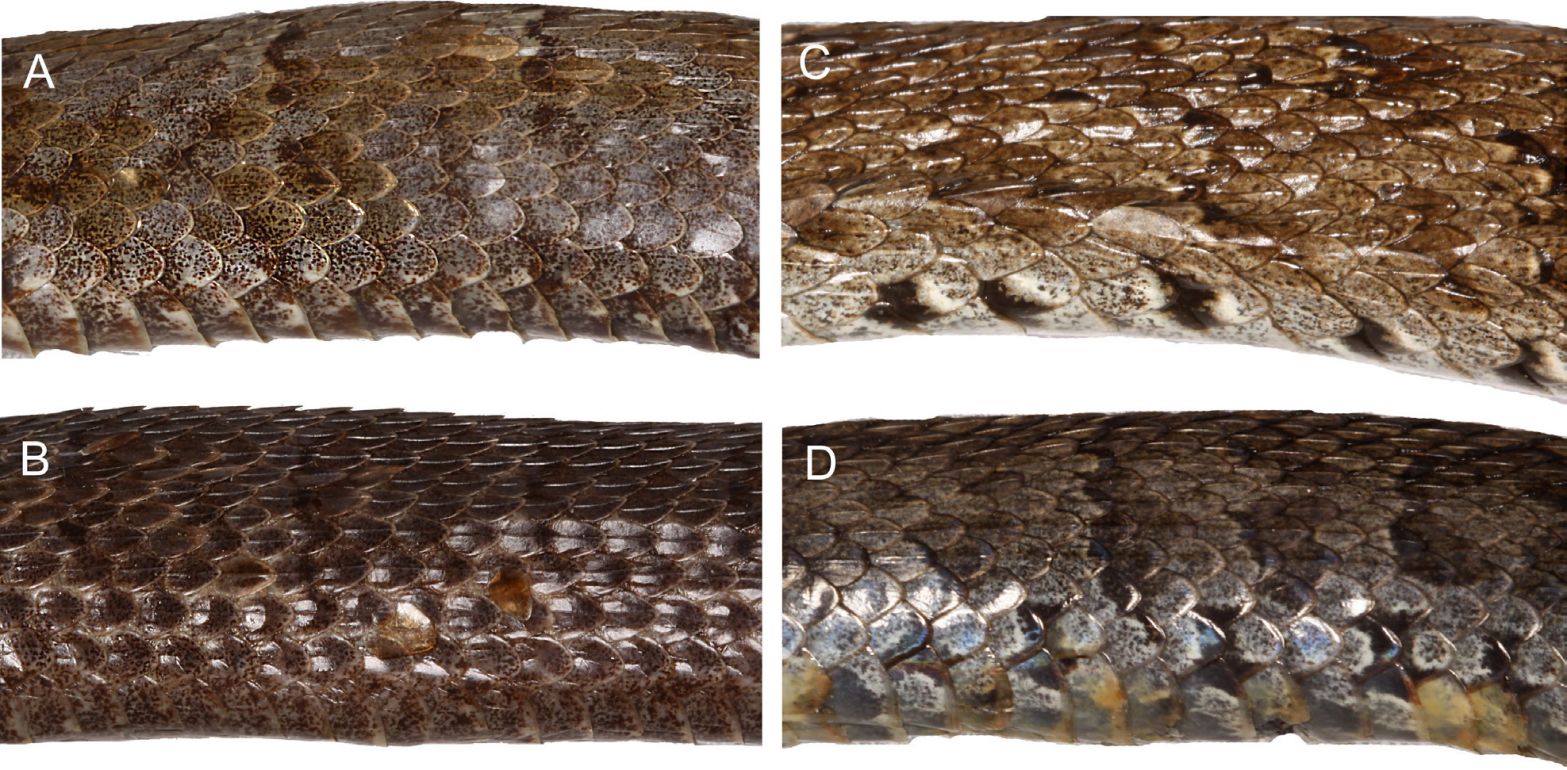
Dorsolateral scalation and pattern in four species of the *Gloydius himalayanus* complex: **A**. *Gloydius himalayanus* (BMNH 1946.1.19.64, lectotype); **B**. *G. hazarensis* sp. nov. (UF 70652, holotype); **C**. *G. hindukushensis* sp. nov. (NHMW 41993, holotype); **D**. *G. nepalensis* sp. nov. (ZMB 65613, holotype).

Distinct interspecific differences in dorsal scale morphology were also directly observed among the examined *Gloydius* taxa (Fig. 6A–D). *Gloydius himalayanus* (Fig. 6A) exhibits broad, oval, distinctly keeled dorsal scales with dense dark speckling and a contrasting pale background, producing a reticulated dorsal pattern. In contrast, the Hazara region lineage (Fig. 6B) has slightly elongated, heavily pigmented scales with stronger keeling and darker overall colouration, resulting in a nearly uniform brown appearance in the examined specimen. The Hindu Kush lineage (Fig. 6C) presents smaller, rounded dorsal scales with clear keeling and fine speckling concentrated posteriorly along a few scale margins. The lineage from the central Himalaya in Nepal (Fig. 6D) is characterised by rather oval scales with reduced keeling and ornamentation laterally.

### Osteological differences in the *Gloydius himalayanus* complex

**Figure 7.**
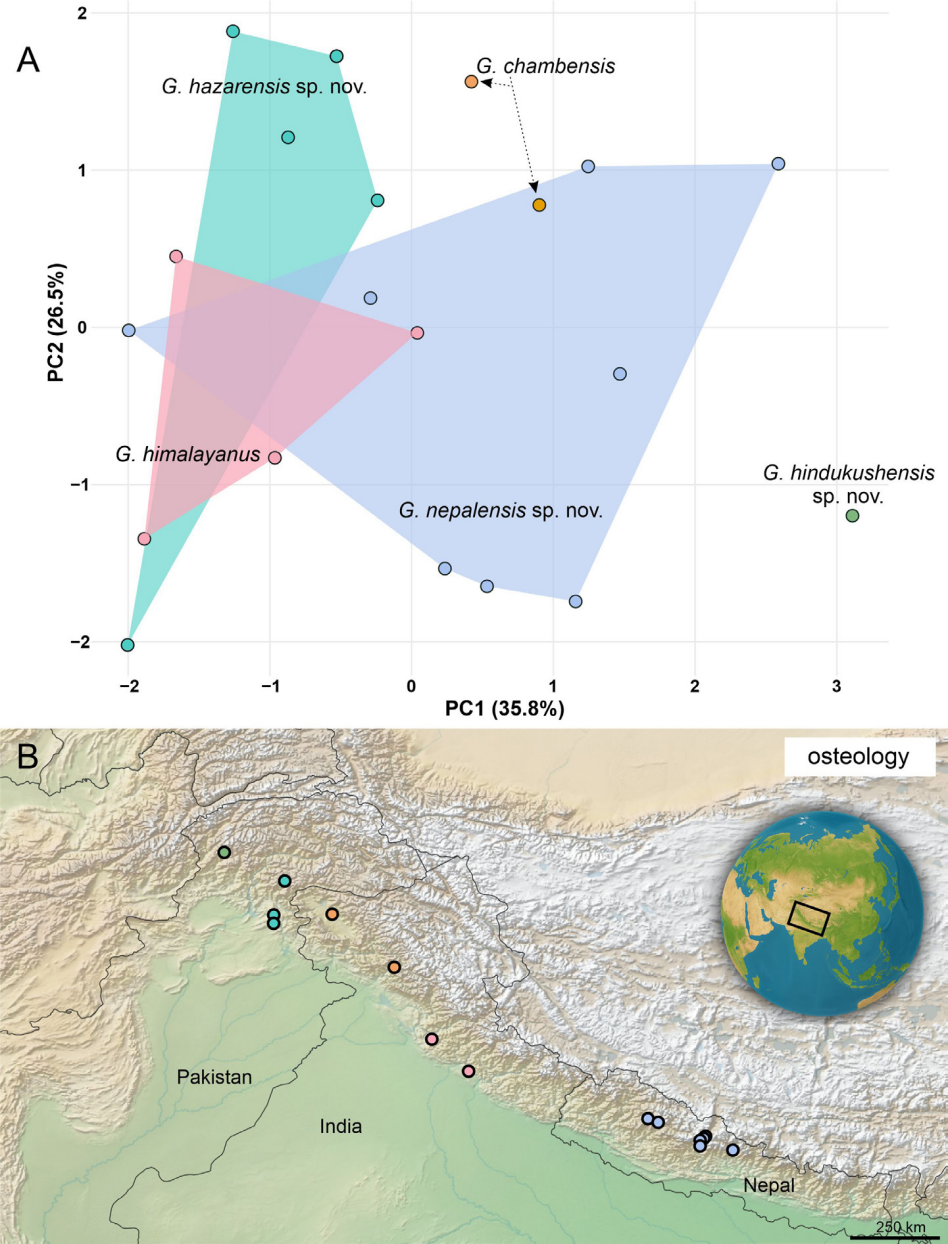
Principal Component Analysis (PCA) of osteological differentiation among species of the *Gloydius himalayanus* complex. **A**. Scatterplot of the first two principal components (PC1 and PC2) based on osteological ratio traits. Points represent individual specimens, colour-coded by species (Figs 1, 2), and convex hulls (shaded polygons) indicate the overall spread of each taxon in morphospace. Dashed arrows are included only as visual guides to displaced labels and have no analytical meaning. Axis labels indicate the percentage of variance explained by each principal component; **B**. Geographic distribution of sampled populations included in the osteological analysis across Pakistan, India, and Nepal. The inset shows the regional context within Central and South Asia. The map was produced in QGIS 3.28.

Principal component analysis of nine osteological variables for 21 specimens (Fig. 7A) showed that PC1 and PC2 explained 35.8% and 26.5% of the variance, respectively. PC1 represents a dentition-richness gradient driven by a higher number of dentary teeth (Teeth Dent), number of pterygoid teeth (Teeth Pt), number of palatine teeth (Teeth Pal), and a larger ratio of discharge orifice length to fang length (OL.FL). PC2 contrasts mandibular proportions, i.e., higher ratio of splenial–angular length to compound bone length (SAL.CpbL), higher percentage that the dental spans the compound bone (DL.CpbL.perc), and higher percentage of toothless part length of total pterygoid length (PteS.PteL.perc) against increased number of palatine teeth (Teeth Pal) and a larger ratio of fang length to osteological head length (FL.HL). Ratio of pterygoid length to pterygoid width (PteL.PteW) contributes minimally. Overall, PC1-PC2 scatter shows lineage-level structuring with partial overlap among the five lineages of the *G. himalayanus* complex.

**Figure 8.**
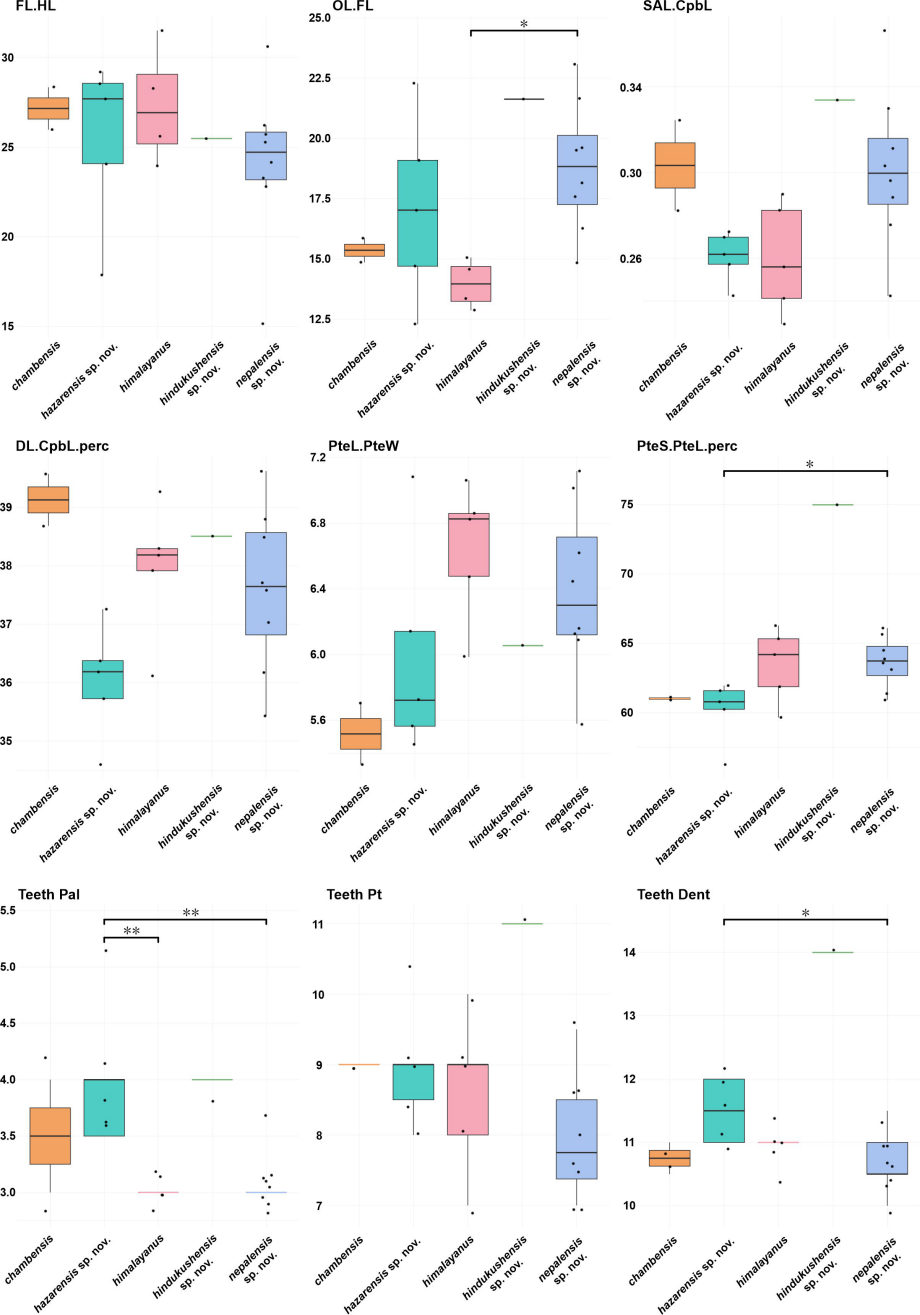
Boxplots showing variation in selected osteological characters (see Materials and methods) among the *Gloydius himalayanus* complex. Measurements include osteological ratios and tooth counts. Boxes represent the interquartile range (IQR) with median values indicated by horizontal lines; whiskers extend to 1.5 × IQR, and dots show individual data points. Asterisks (*) and double asterisks (**) denote statistically significant differences between species (*p* < 0.05 and *p* < 0.01, respectively). Boxplots are colour-coded according to genetically defined lineages (Figs 1, 2).

Boxplot analyses of osteological characters (Fig. 8) revealed several statistically significant differences among the five lineages of the complex (ANOVA or Kruskal–Wallis tests, *p* < 0.05). The ratio FL.HL showed moderate inter-lineage variation, with the Hazara lineage and *G. himalayanus* tending toward relatively longer fangs, whereas the lineage from the central Himalaya in Nepal exhibited the lowest values. The ratio OL.FL differed significantly between the central Himalayan lineage and *G. himalayanus* (*p* < 0.05), the latter showing a shorter relative orifice length (but see the Hazara lineage). Proportions associated with the compound bone (SAL.CpbL and DL.CpbL.perc) varied only slightly among taxa and lacked consistent trends. The PteL.PteW was highest in *G. himalayanus*, indicating a more elongate pterygoid, while the PteS.PteL percentage differed significantly between the Hazara lineage and the lineage from the central Himalaya in Nepal (*p* < 0.05). Dentition characters showed the most distinct contrasts. The number of palatine teeth (Teeth Pal) was significantly higher in the Hazara lineage than in both *G. himalayanus* and the lineage from the central Himalaya in Nepal (p < 0.01). Dentary tooth counts also differed among taxa (*p* < 0.05), with the highest value observed in the Hindu Kush lineage, although this lineage was represented by a single specimen. In contrast, Teeth Pt remained largely overlapping across taxa except for the Hindu Kush lineage.

### Revised general diagnosis based on our own data modified from Gloyd and Conant (1990)

Pitvipers of the *Gloydius himalayanus* complex can be characterised as follows: medium-sized (max. recorded length of examined specimen 720 mm, in literature, 864 mm (34 inches, Stoliczka 1870) with stout body and a short tail (ratio tail/total length 0.108–0.202), males are usually slightly larger than females; body and tail subcylindrical; head triangular in shape, clearly set off from the neck, tip of the snout usually protruding, canthus rostralis well developed; ground colour usually brownish or greyish with variable patterns, e.g., (1) dark markings consisting of 23–45 crossbands, or (2) a double row of alternating dorsolateral dark brown roundish markings with openings to the venter which can be edged with darker margins, or (3) with two rows of lateral (sometimes interrupted) dark brown bands, or (4) almost monochrome dark greyish or brownish with barely identifiable signs of markings; tail as described for the body, but usually with barely identifiable irregular markings, tip of tail usually much lighter in colour; dorsal head with or without irregular dark markings; eye with a vertical slit-shaped pupil, the latter being distinctly darker than the iris in living specimens, but lighter in preserved specimens; a dark postocular stripe (very rarely absent) of varying width and intensity covering, depending on its width, the temporal and/or upper supralabial region and extending to the end of the mouth or behind it in the neck area; supralabials usually much lighter than the rest of the lateral head colouration; venter of head cream coloured and heavily powdered with dark speckles; venter of body and tail shows a metallic lustre with a colour gradient from pale cream in the anterior part to almost black-brown at the posterior body; venter heavily powdered with dark speckles.

Scutellation is characterised by a rostral wider than high and hardly visible from above; by narrow internasals and large prefrontals which are pointed anteriorly but with straight or concave posterior margins; large supraoculars, longer than wide; a bell-shaped frontal longer than wide, sometimes an azygous tiny scale on its anterior or posterior border; parietals longest of all head shields, longer than wide; 10–20 scales bordering the posterior edge of the pileus; nasal completely or partially divided, loreal subquadrangular, mostly a little higher than wide; usually two, in western populations sometimes three preoculars, the upper reaching top of head and forming part of the canthus rostralis; pit opening encircled by three scales and as long as or shorter than horizontal eye diameter; two or three, rarely four postoculars, the lower (postsubocular) sometimes extends up to the level of anterior border of eye; temporals variable, usually three, sometimes two or rarely one anterior temporal followed by three or sometimes four in the posterior row; predominantly seven, rarely six or eight supralabials, second smallest, second last highest (sometimes interpreted as fused with the lower temporal scale row), third or rarely second in contact with the eye; nine or ten, rarely eight or 11, exceptionally seven sublabials, first three or four in contact with the anterior submaxillars; anterior pair of submaxillars broader and longer than posterior pair; mean number of gulars in contact with submaxillars is 5.01; dorsals strongly keeled, except the outer row, arranged in 21 (sometimes 23, rarely 20, 22 or 25)/21(sometimes 19, rarely 20, 22 or 23)/17 (rarely 15 or 16) rows; apical pits paired, very weakly developed, and not detectable in preserved specimens if the Oberhäutchen is missing; dorsal scale reduction from 23 to 21 scales appears on the level of 4–39% of ventrals, from 21 to 19 scales on the level of 41–76% of ventrals, and from 19 to 17 scales on the level of 64–89% of ventrals, for a summary of main dorsal scale reduction of examined material we refer to Tables 5–8; usually two or three, sometimes four or rarely one or no preventrals, followed by 143–167 (maximum 170 according to Gloyd and Conant 1990) ventrals without keels; cloacal scute invariably entire; 35–54 (minimum 33 according to Manhas 2020) paired subcaudal scales, exceptionally individual undivided subcaudals possible; total number of all ventral body and tail scales varies from 185–218; hemipenial characters are known from a specimen originating from Pir Panjal Range, Kashmir, India (AMNH 39395, examined in situ) and were described by Gloyd and Conant (1990) as follows: eight subcaudals in length, long-forked for half of their length, proximal to the fork five very large spines; a cluster of large spines occupies two thirds of the length of the asulcate side of the organ; small spines adjacent to the sulcus spermaticus merge with calyces on the distal third of rami; lips of calyces smooth; sulcus forks at level of one and a half subcaudals and extends to the tip of the organ; sulcus lips smooth at the base, becoming spinous or calyculate to match the ornamentation of surrounding tissue. Dentition is generally characterised by two movable, hollow, long, curved, conical, and sharply pointed fangs at the rear end of the maxillary bone (solenoglyph); ratio fang length/head length, 3.17–6.61 (mean 4.07; 6.86 according to Ernst 1982b); number of replacement fangs varies from 5–8 (4–10, according to Ernst 1982b); three to four slightly decreasing teeth on the highly compressed palatine bone; 6–12 subequal teeth on the anterior pterygoid bone; and 10–14 teeth, gradually decreasing in size, on the mandibular bone.

### Distribution of the *Gloydius himalayanus* complex

**Figure 9.**
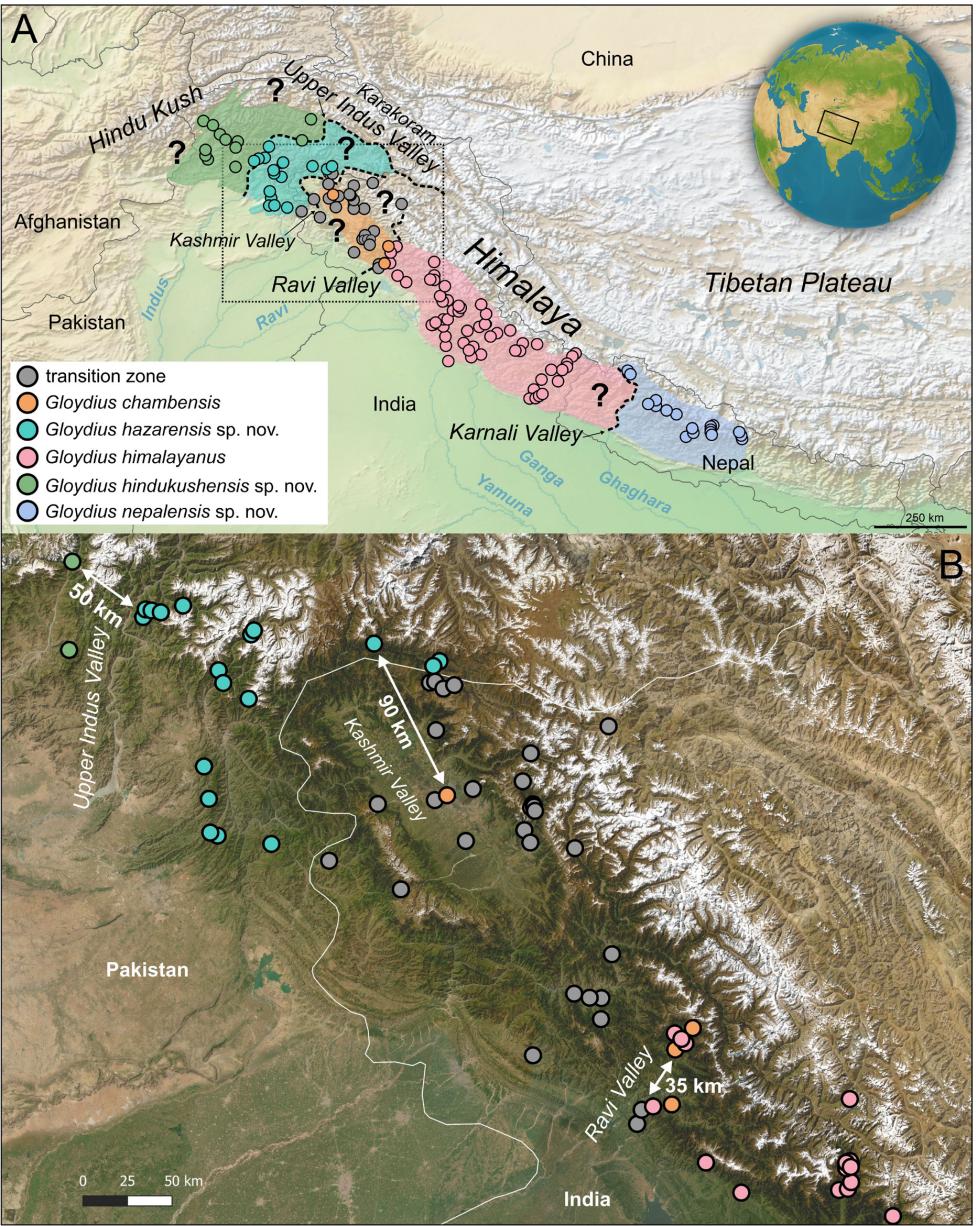
**A**. Geographic localities of species within the *Gloydius himalayanus* complex, compiled from literature sources, museum records, citizen science platforms (iNaturalist 2025), and data obtained in this study (see Gazetteer Suppl. material 1: table SS3 for details). Grey circles indicate ambiguous records or populations of uncertain taxonomic status, where evidence was insufficient for reliable identification ("transition zone"). Dashed lines delineate hypothesised biogeographic boundaries between major lineages, i.e., the Upper Indus Valley, Kashmir Valley, Ravi River Valley, and Karnali River Valley, landscape features likely acting as geographic barriers influencing species distribution; **B**. Inset map of the Kashmir region (Western Himalaya) showing the distributions and contact zones of the taxa defined here, highlighting a key area for future research. Question marks highlight regions that remain poorly explored or unsampled. The map was generated using QGIS 3.28 (https://qgis.org/) with the WGS 84 coordinate reference system.

Based on the literature, preserved museum voucher specimens, the citizen science portal iNaturalist.org (2025), personal communications from colleagues, and our own data, we compiled a dataset of 194 locality records assigned to specimens of the *G. himalayanus* species complex (Fig. 9 and Gazetteer in Suppl. material 1: table SS3). This represents the largest dataset ever assembled for the distribution of this group of snakes. The species complex has a broad extent of occurrence (EOO), covering ca 162,000 km^2^ from the eastern Hindu Kush in Pakistan across the Himalaya of Pakistan and northwestern India to the eastern flank of the Annapurna Massif in west-central Nepal. However, this EOO, like those for the individual species within the complex, is based on a minimum convex polygon; therefore, the actual range is certainly smaller, as suitable habitats are not uniformly present across the entire region. Of the records, 135 originate from northwestern India, 33 from Pakistan, and 26 from Nepal (Fig. 9).

The westernmost evidence comes from Chitral and the upper Shishikoh Valley, from Upper and Lower Dir, Swat, the Kumrat Valley in western Kohistan and the Palas Valley in Indus Kohistan. It is recorded from the Kaghan and Neelum River Valley. To the north of this, the distribution extends from eastern Dardistan as far as Gilgit just south of the western foothills of the Karakoram Range. There are numerous reports from Kashmir (Kashmir denotes Azad Jammu and Kashmir in Pakistan and a section of Jammu and Kashmir State of India), especially from the Liddar River Valley as far north as the eastern edge of the Dardistan Pangi Range in southwestern Ladakh and evidence from the mountains bordering the region to the south, i.e., Punch and Pir Panjal Range. Further east known records often come from the traditional transit routes following the higher valleys of the Chamba, Kangra, Kullu (Beas), and Sutlej River. There are numerous records from the Garhwal Himal, particularly from the region north of Dehradun, up to the Sankari Range and the Tons River Valley, as well as from the Kumaon mountains with the Bhowali Range and in the north up to the Dungair Dhar. The southern boundary in the western Himalaya from Kashmir to Kumaon is the Sivalik (Shiwalik) Range, the southernmost orogenic element of the Himalayan system.

In the central Himalaya (Nepal), the distribution area extends in the west from ca 81°42'E on the upper Karnali River in the Greater Himalaya via the Dhaulagiri Himal to the eastern edge of the Annapurna Himal where the upper Marshyangdi River at ca 84°22'E represents the currently documented distribution limit. The southern boundary in the central Himalaya is the Mahabharat Range (Lesser Himalaya).

The minimum elevational limit recorded was 670 m for Dehradun, Uttarakhand, India (SMNS 4374) which is very likely incorrect, as no other specimen has been found so far in the valley during the past century. The specimen most probably originates directly north of Dehradun, where the mountain ranges bordering the valley rise very quickly to more than 1500 meters and where several other specimens have been recorded (see Gazetteer, Suppl. material 1: table SS3). The probable lowest reliable record i.e., Ramnagar, Jammu and Kashmir State, at ca 800 m elevation, came from Sahi and Duda (1985). The highest reported locality "Dharmsala at the foot of glacier, 16.000 ft" [Himachal Pradesh State, India, ~4877 m, ZSIK 12875] was introduced by Sclater (1891) and since then has been listed as one of the highest-elevation reptile records (Schleich and Kästle 2002). Interestingly, the altitudinal information is questionable, in that the mountain ranges and glaciers within a 10-km radius of Dharmsala hardly exceed the altitude of 4500 m. Specimens of the *G. himalayanus* complex are found mostly in higher elevations at an average of 2250 m, ranging from the hilly to the subalpine zone, and records above 3500 m and below 1000 m are very rare. As a result, migration, if it occurs at all, between localities is unlikely, especially in a west-east or vice versa direction, as these areas are typically separated by deep valleys that primarily drain from north to south. The tentative distribution of each species is provided in the "Distribution" section below.

For the western part of the distributional area of *G. himalayanus* (auct.) Leviton and Anderson (1970) mentioned "Wama, Nuristan" in eastern Afghanistan with reference to a specimen in the collection of the University of Copenhagen (NHMD R-6911). However, the specimen was subsequently identified as *Vipera lebetina* (currently *Macrovipera lebetinus*) by Rasmussen in Nilson (1983) (see also comments in Gloyd and Conant 1990 and Wagner et al. 2016a). This erroneous record from Afghanistan still appears in the literature (Shah and Tiwari 2004; Bahuguna 2010; Sindaco et al. 2013). The first mention of *G. himalayanus* (as *Trigonocephalus blomhoffii* Boie (*Tr. affinis* Gray)) for the eastern Himalaya, i.e., "Sikkim" is by Jan and Sordelli (1874: liv. 46, pl. 5, fig. 4) who depicted a specimen from the collection of the Museo civico di storia naturale di Milano, Italy, which was probably destroyed in 1943 (Scali 2010). The occurrence in Sikkim has not yet been confirmed by new evidence. Mentions for eastern Nepal, e.g., by Rai (2002), Schleich and Kästle (2002), Shah and Tiwari (2004) or Kästle et al. (2013) are based on misidentification (see comments below for the new lineage from Nepal). Wall (1910) comments on a communication from Mr G.A. Miller, who claims to have received a single specimen from Darjeeling (West Bengal State, NE India) and another specimen (ZMB 8452) with the same questionable locality information which came from the T.C. Jerdon’s collection to the Zoological Museum Berlin in 1881. The occurrence in Sikkim and West Bengal could not be confirmed by Shaw et al. (1933, 1947, 1999). Recently, Chaudhuri et al. (2018) mentioned a photographic record from Kolakham (Kalimpong District, West Bengal State, NE India), interestingly as the first record from West Bengal State (but see above). The photo vouchers of this snake, which is said to originate from northern West Bengal, are archived in the collection of the Lee Kong Chian Natural History Museum in Singapore under ZRC IMG 2.385a–b and were made available to us by the collection’s curator. A re-examination of the snake depicted in the two photos (dorsolateral close up of head and forebody and dorsolateral full body) revealed that it is indeed a species of the genus *Gloydius*. Because of the shape and size of the posterior supralabials and the arrangement and size of the temporals, this specimen does not match the conditions found in the *G. himalayanus* complex. In contrast, it shows major similarities to *Gloydius brevicaudus* in terms of head pholidosis and head and body colour pattern. It is unclear how a specimen of this species, which is found exclusively in East Asia, ended up in northern West Bengal and was photographed there. Three specimens, also from the Jerdon collection, which are said to come from the "Khassyas" (Khasi Hills, Meghalaya State, NE India) are deposited in the Natural History Museum London (BMNH 1872.4.17.352–354) but doubts about the origin of this material have been expressed already in the past (Boulenger 1896; Wall 1910; Smith 1943; Kuttalam et al. 2022). The occurrence of *G. himalayanus* (auct.) further east in Bhutan was surmised by Lenz (2012) or stated by Wangyal (2014), Koirala et al. (2016), Chaudhuri et al. (2018), and included in the recent book on Bhutanese reptile fauna by Wangyal and Das (2021) without providing direct evidence in the form of voucher material or photos from local specimens. Frequently even Bangladesh (Bahuguna 2010) or southern China (Wangyal and Das 2021) are considered within the distribution area of this taxon.

As the above-mentioned dubious or incorrect distribution records lie outside the western and eastern distributional limits and lack confirmation by physical specimens or photographs, they are excluded from the following discussion.

### Taxonomic conclusions

Our integrative approach, combining molecular phylogenetics, genetic distance analyses, and evidence from morphology, osteology, ecology, and distribution, supports the existence of at least five distinct lineages within the *G. himalayanus* complex. These identified lineages exhibit deep genetic divergence, clear morphological differentiation, and likely geographic isolation imposed by mountain topography and river valleys, which together support their recognition as distinct species within the *G. himalayanus* complex, consistent with a lineage-based and integrative taxonomic framework (Baker and Bradley 2006; Dufresnes et al. 2023; Wüster et al. 2024). Of these five lineages, two correspond to species recognised previously, whereas three represent newly identified lineages that we formally describe here as new species.

#### Gloydius chambensis

Taxon classification

Animalia

SquamataViperidae

Kuttalam et al., 2022

A92D0402-9B6C-5BEA-B7D5-8D671D1BE6A7

##### Common name.

Chamba Pitviper

##### Type material examined.

***Holotype***. • HARC R259 (DNA reference number 18.13), an adult male from Bhanjraru village, Chamba District, Himachal Pradesh, India, 32.8391°N, 76.1493°E, 1738 m altitude, found dead on road on 10 July 2018 by Kuttalam, Vishal Santra, John Benjamin Owens, Vipin Dhiman, Anita Malhotra, Nilanjan Mukherjee, Stuart Graham, and Anatoli Togridou. ***Paratype*(s)** not designated (Kuttalam et al. 2022).

##### Revised diagnosis.

A single loreal, wider than high; nasal completely divided, or partly divided below the naris; always two elongated preoculars, the lower narrower; a single supraocular; two postoculars, upper reaching onto top of head and touching the parietal, the lower can be described as postsubocular and touches, with its anterior side, the upper posterior edge of the third supralabial; rostral scale wider than high; two internasals wider than long; two prefrontals, ratio of length to width variable sometimes almost the same length; frontal bell-shaped, longer than wide; predominantly three, exceptionally one anterior and predominantly three or sometimes four posterior temporals; usually seven, rarely six or eight supralabials, always the third in contact with the eye; 15–18 circum-pileus scales; usually ten, sometimes nine, rarely eight sublabials, predominantly first three, sometimes first four, rarely only first two in contact with anterior submaxillars; two pairs of submaxillars, anterior usually twice as wide and 30–43% longer than posterior; followed by three rows of paired gular scales, increasing in size posteriorly; anterior dorsal scales in 21, 23 or 25 rows, midbody dorsal scales in 21 rows, posterior dorsal scales in 17 rows; 0–5 preventrals; 157–164 ventrals (males 162–164, females 157–163); cloacal plate entire; 41–49 usually paired subcaudal scales (males 42–49, females 41–48), rarely up to two subcaudals undivided; sum of ventral and subcaudal scales 204–218 (males 211–218, females 204–217). Body compact, subcylindrical; tail short, ratio TaL/TL of 0.129–0.202 (males 0.153–0.202, females 0.129–0.137); maximum recorded SVL in examined material: males 476 mm, females 527 mm; maximum recorded TaL in examined material: males 86 mm, females 84 mm; maximum recorded TL of examined material in males 562 mm, in females 611 mm.

##### Sum formula of dorsal scale reduction.

**Table:** 

(8–22)	(67–108)	(108–111)
4+5, 5+6	3+4, 4+5, 5+6, 6+7	3+4, 4+5
**(5)23----------------------------------21---------------------------------------19-------------------------------17**.		
4+5, 5+6, 6+7	3+4, 5+6	3+4, 4+5
(8–21)	(67–108)	(105–118)

##### Variation in dentition.

**Figure 10.**
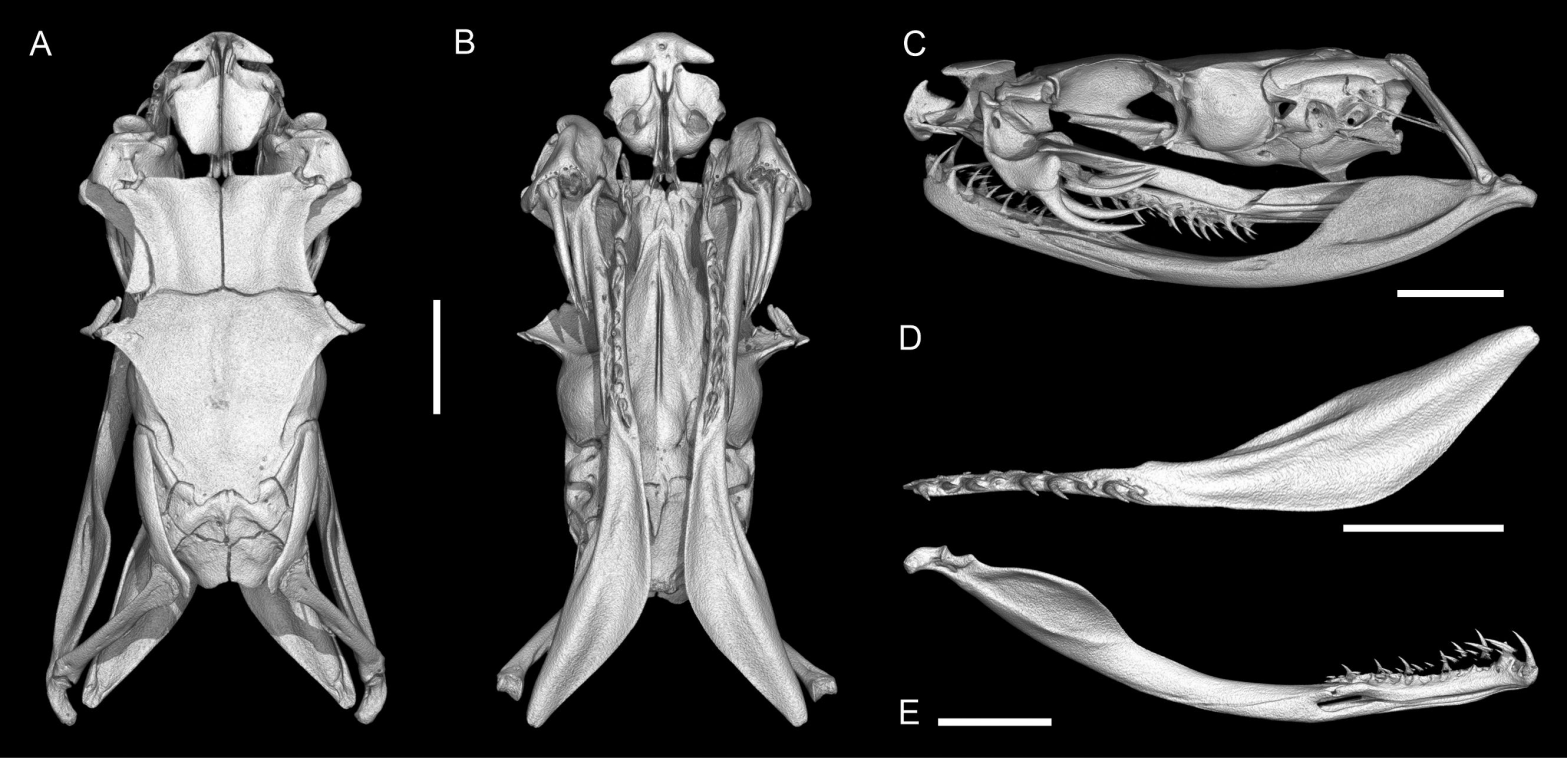
Skull of the adult male of *Gloydius chambensis*BMNH 1898.5.17.4: **A**. Dorsal; **B**. Ventral (lower jaws virtually extracted); **C**. Lateral view; **D**. Pterygoid virtually isolated, ventral view; **E**. Lower jaw virtually isolated, medial view. Scale bars: 4.0 mm.

Maxillary bone with two posteriorly curved fangs on each side. Main fang 4.98–5.45 mm in length, i.e., 26.0–28.4% of skull length. Discharge orifice 0.79–0.81 mm in length, i.e., 14.9–15.9% of fang length. Palatine bone with three or four posteriorly curved teeth slightly decreasing in size posteriorly. Pterygoid bone with nine posteriorly curved teeth, shorter than the palatine tooth, all nearly the same size. The posterior 60.9–61.1% of the pterygoid bone is without teeth. Mandibular bone with 10 or 11 posteriorly curved teeth gradually decreasing in size posteriorly. The first two teeth closer together than the rest. Splenial either 67.1% of length of angular or fused. The total length of splenial-angular complex spans 28.2–32.4% of the mandibular bone. The dental is 38.7–39.6% as long as the mandibular bone. The complete skull of the specimen BMNH 1898.5.17.4 is presented in Fig. 10.

##### Variation in life colouration and pattern.

**Figure 11.**
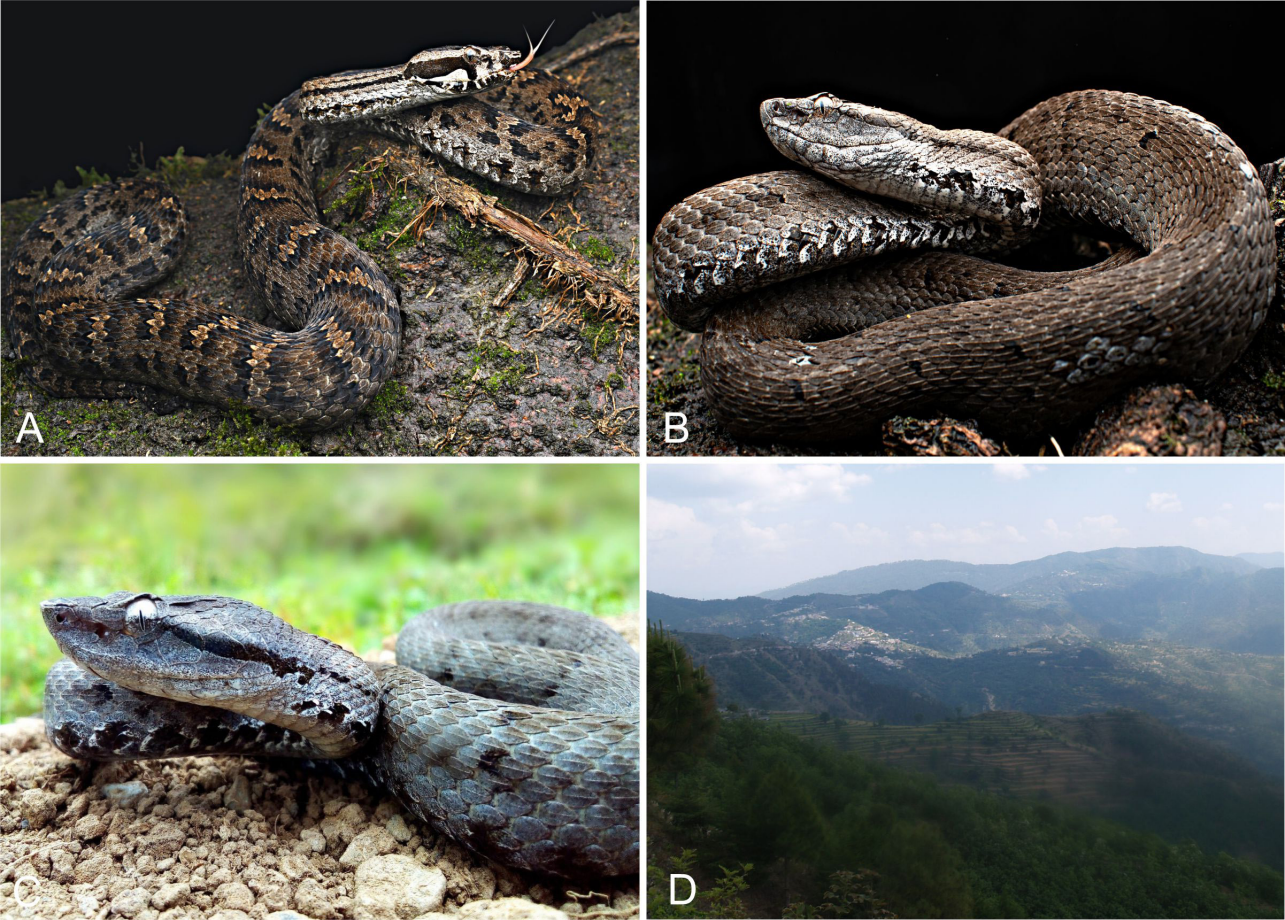
The colour and pattern in *Gloydius chambensis* and its typical habitat. **A**. The specimens (uncollected) from Teppa, Chamba District, Himachal Pradesh State, India, 2543 m elevation (photo by Girish Choure); **B**. *Gloydius chambensis* (uncollected) from Teppa, Chamba District, Himachal Pradesh State, India, 2543 m elevation (photo by Girish Choure); **C**. Specimens from Chamba Valley (photo by Rakeshwar Kapoor, https://www.inaturalist.org/observations/52247726); **D**. General view of the Chamba Valley (https://commons.wikimedia.org/wiki/File%3AChamba.jpg).

Dorsal ground colour can vary from Cinnamon-Drab (Colour 50) to Smoke Gray (266), with 32–46 very thin Sepia (279) coloured irregular cross bands their anterior borders sometimes edged with Cream White (52), the bands may be broken on the back and offset from each other; tail with 10–12 thin Sepia (279) crossbands; a row of small, irregular Sepia (279)-coloured and Cream White (52)-bordered spots runs along the outer edge of the ventrals; dorsal and lateral head same as dorsal body colour or Pale Neutral Gray (296) and densely mottled with Sepia (279); a Sepia (279) coloured postocular stripe with a mainly straight thin Cream White (52) lower margin runs from the posterior border of the eye to the posterior edge of the mandible, the height of the anterior part of the postocular stripe is variable and can cover up to two-thirds of the anterior lower temporal scale; neck with two paravertebral and two lateral wavy Sepia (279) coloured stripes and a very thin and short one in the middle of the dorsal head; venter with a colour gradient that becomes darker towards the tail; throat Pale Neutral Gray (296) coloured, gulars mottled with Dark Grayish Brown (284); subsequently, the venter ground colour changes gradually to Light Neutral Gray (297) and shows heavy Sepia (279) mottling; tail tip lighter. Examples of variation in the colouration and pattern of live individuals; see Fig. 11.

##### Distribution.

*Gloydius chambensis* is a narrowly distributed species of pitviper endemic to the western Himalaya, originally known only from the Chamba District of Himachal Pradesh, India. Confirmed records based on genetic and morphological data include the type locality Bhanjraru (1738 m) and Teppa (2543 m) in the Churah Valley. However, our results have significantly expanded the known distribution of the species. Based on a combination of distributional data and newly generated genetic evidence from museum specimens (BMNH 1898.5.17.4–5 and NHMW 17079:1), the known range of *G. chambensis* has been extended westward by approximately 260 km into the Kashmir Valley, specifically to Srinagar, where NHMW 17079:1 was collected. This extension suggests that the species may occupy a polygonal range of up to 16,000 km^2^ (less when considering suitable habitats only; Fig. 9).

##### Habitat and ecology.

*Gloydius chambensis* occurs primarily in alpine scrub and temperate pine forests, often in areas with thick pine needle litter on the forest floor. The terrain is typically rocky and interspersed with boulders and rock faces, providing natural shelter and basking spots. It inhabits an elevational range of ~400–2500 m in the lower montane to subalpine transition zone (Fig. 11), although most records for *G. chambensis* fall within the lower temperate zone (~1700 m; Kuttalam et al. 2022), particularly in the Churah Valley.

##### Conservation.

Despite this ecological specificity and documented human interaction (Kuttalam et al. 2022), the species remains unassessed by the IUCN Red List and unprotected under national conservation frameworks. The combination of its narrow range (but see the specimen from Kashmir Valley, NHMW 17079:1), morphological and genetic distinctiveness, and known anthropogenic threats such as forest degradation, increasing road and rural development, underscores the urgent need for targeted conservation measures. These should include especially habitat preservation, ecological monitoring, community engagement programs, and formal threat assessment to support its inclusion in conservation priority lists.

#### Gloydius himalayanus

Taxon classification

Animalia

SquamataViperidae

(Günther, 1864)

198FFC36-4257-56CB-A282-03A75C34EC93

Gloydius himalayanus (Günther, 1864): 393, pl. 24, figs A, A'.

##### Common name.

Himalayan Pitviper.

##### Type material.

**Figure 12.**
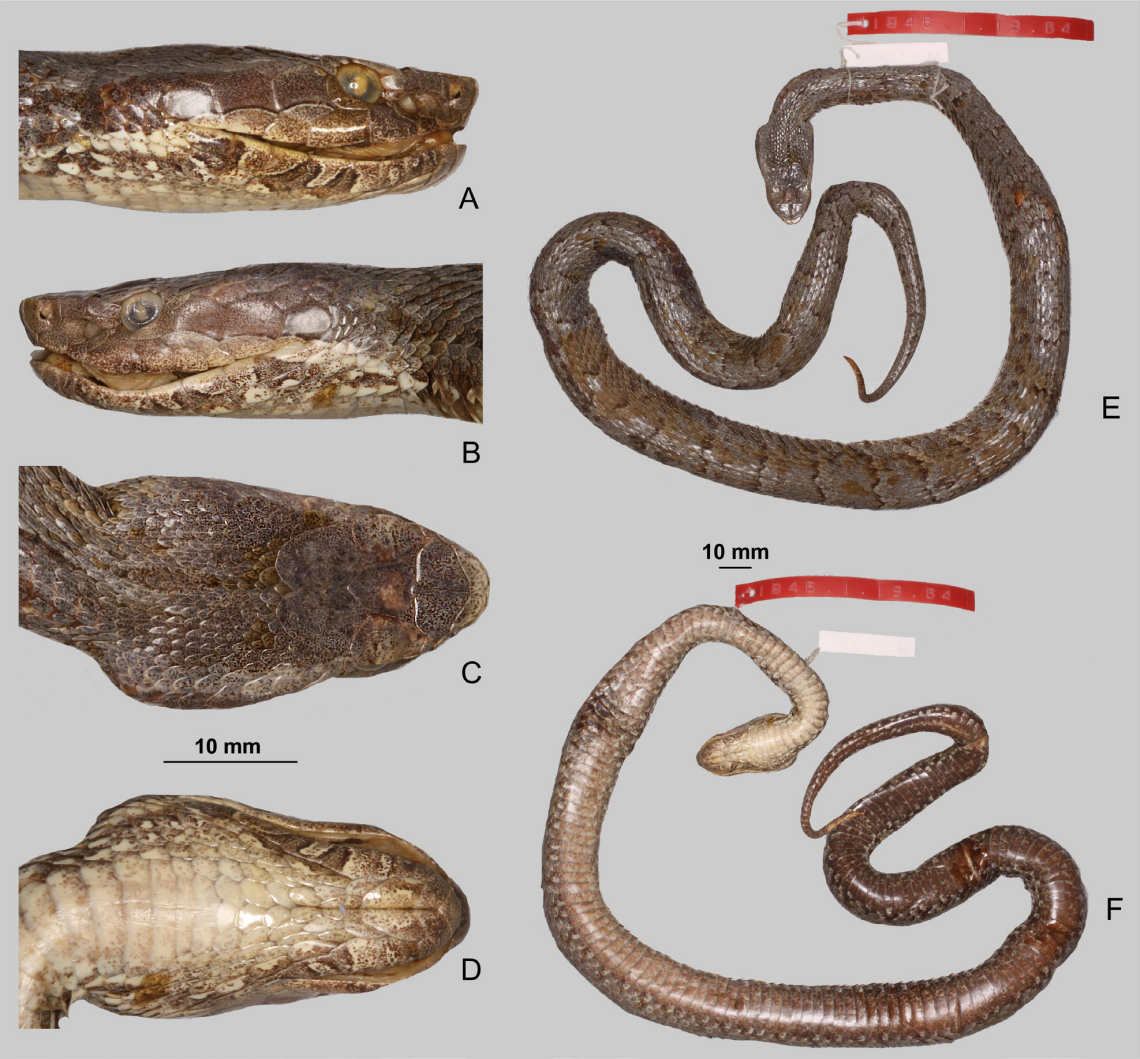
The adult male lectotype of *Gloydius himalayanus* (BMNH 1946.1.19.64). **A–D**. Lateral, dorsal and ventral views of the head; (**E, F**) dorsal and ventral views of the body.

The original description of *Halys himalayanus* Günther, 1864 is based on two syntypes originating from "Garhval, Himalaya (altitude 9000 feet)". Both specimens are preserved in the collection of the Natural History Museum, London: BMNH 1946.1.19.64 (Figs 1, 12; formerly BMNH 1860.3.19.1358), collected by the German explorers, the von Schlagintweit brothers; and BMNH 1946.1.18.75 (formerly BMNH 1860.3.19.1189), originating from the collection of the Danish physician and biologist Theodore Edward Cantor.

On behalf of the British East India Company (BEIC), and with financial support of the Prussian king Frederick William IV, and the monarch Maximilian II of Bavaria, the brothers Hermann, Adolf, and Robert von Schlagintweit undertook a four-year scientific expedition from 1854–1858 to explore the Indian subcontinent and its northern high mountain regions. They travelled, sometimes together, but also on separate routes, through India and the Himalaya up to the Tibetan plateau [Schlagintweit-Sakülünski 1871; on the controversial debate about the achievements of the expedition see Brescius (2015) and Kleidt (2024)].

The natural history objects collected during the von Schlagintweit expedition were the property of the BEIC but were brought to Berlin for scientific analysis and were then to be successively returned and reintegrated in the BEIC collections in London. Between 1858 and 1859, an unknown number of stuffed animals, as well as wet preserved reptiles and fishes were transferred from Berlin to the India Museum of the BEIC whose whereabouts are now unknown according to Kleidt (2024). Sharpe (1906) mentioned that the transfer of part of the India Museum collection to the BMNH began in 1860 (1859 according to Archer 1962: 63), but the final incorporation of the old BEIC collection took place in 1880 (1879 according to Kleidt 2015). Additionally, Günther’s (1860) overview of the herpetofauna of the Himalaya, which includes 118 listed specimens of amphibians and reptiles referable to material collected by the von Schlagintweit brothers, clarifies that objects from the expedition, including documents stating localities and altitudes at which each specimen was obtained, had already been handed over from the Museum of the BEIC specifically to him for examination (i.e., no later than May 1860 when his paper was published). Many of these specimens are subsequently listed in the published catalogues of the holdings of the herpetological collection of the BMNH (see Boulenger 1885, 1887, 1889, 1893, 1894, 1896). In his 1860 paper, Günther at the time working on the classification of the BMNH snake collection, mentioned also the von Schlagintweits’ specimen, which later served as one of the two syntypes of *Halys himalayanus* for the first time under the name "*Trigonocephalus affinis*" (Günther 1860: 164, 167, 172). It is not documented in the records of the herpetological department of the NHM London which of the von Schlagintweit brothers collected the specimen, and Günther’s (1860) paper, and his (1864) original description, also provide no clue.

According to the itinerary and map of the routes taken by the von Schlagintweit brothers in the western Himalaya, it becomes clear that only Adolf and Robert had travelled in Kumaon and Garhval between early July and mid-October 1855 (Schlagintweit et al. 1861a: 17 ff.). From the listed stations no. 50–55 visited in "*Kămáon and Gărhvál*" only the following are situated in Garhval as defined at that time by the von Schlagintweit brothers: "Mána (in Vishnuganga valley, two miles north of the Hindi temple Bádrinath, 30°47'0"N, 79°20'50"E, 10,670 feet)", "Mána Pass (two days journey north of Mána, 31°5'0"N, 79°23'25"E, 18,852 feet)", "Ussílla (highest village in the valley of Tons, 31°7'40"N, 78°18'10"E, 8940 feet)", "Măssúri, Banóg Hill (station, 30°28'30"N, 77°59'58"E, 7549 feet)" (Schlagintweit et al. 1861a: 197 ff., 1861b: map a, 1). Based on the altitude of 9000 feet [2743 m] given by Günther (1860, 1864), the later syntype specimen collected by the von Schlagintweit brothers could have been found in or around "Ussílla". This place is also mentioned for plants collected in early October 1855 by the von Schlagintweit brothers as "Usilla in the Sons [sic, Tons] valley" by Klatt (1880: 369). If the von Schlagintweit’s type specimen was indeed from "Ussílla" [also known as Oshól, a village no longer existing on the right bank of the Tons [Tonse or Supin] River Valley, near today’s Osla village in the southern Supin Range, Uttarkashi District, Uttarakhand State, India], then it was collected by Adolf von Schlagintweit, who was the only one of the brothers to visit this location during his second journey to Tibet on his return to Garhwal between 6–9 October 1855 (Schlagintweit et al. 1861a: 19).

Similar to the von Schlagintweits’ material, the second syntype of *H. himalayanus* was donated indirectly to the British Museum (Natural History) from Theodore Edward Cantor’s collection. Between 1842 and 1854, Cantor sent several general collections to the Museum of the British East India Company (India Museum), London, and many of the natural history specimens were transferred to the BMNH in 1860 (Boulenger 1906; Whitehead and Talwar 1976). Among these objects was probably Cantor’s specimen, which later served as second syntype and was registered by Günther on 19 March 1860, the month of Cantor’s death, under the number 1189. As mentioned above, Günther (1860) only listed the specimen from the von Schlagintweits collection in his treatise on the herpetofauna of the Himalaya. The first part of the Proceedings of the Zoological Society of London, which contains Günther’s work, was published between February and May 1860 (Duncan 1937), and Cantor’s specimen probably reached Günther’s hands too late to be considered.

Cantor worked as a physician in the service of the British East India Company based in Calcutta [Kolkata, West Bengal State, India], where he arrived in 1835. His duties took him to China (Chusan), the Malay Peninsula (Provinces of Malacca and Wellesley, Penang, and Singapore), and Bengal (Sundarbans, Ganges-Brahmaputra Delta), where he had the opportunity to make substantial zoological and botanical collections. It is documented that Cantor was posted to NW India twice during the First and Second Anglo-Sikh War for his military service in field hospitals from 1845–1846 and from 1848–1849 (Adler 2007, 2016; Archer 1962). It is possible that it was during these visits that he obtained the specimen that was later to become the second syntype of *H. himalayanus*.

Because of the better documented historic circumstances (route of travel) for the syntype collected by the von Schlagintweits, and in agreement with Art. 74.7 of the Code (ICZN 1999), we here designate BMNH 1946.1.19.64 as lectotype of *Halys himalayanus* Günther, 1864 to introduce a standard of application for the species group name *himalayanus* Günther using a single name-bearing specimen.

##### Type material examined.

**Figure 13.**
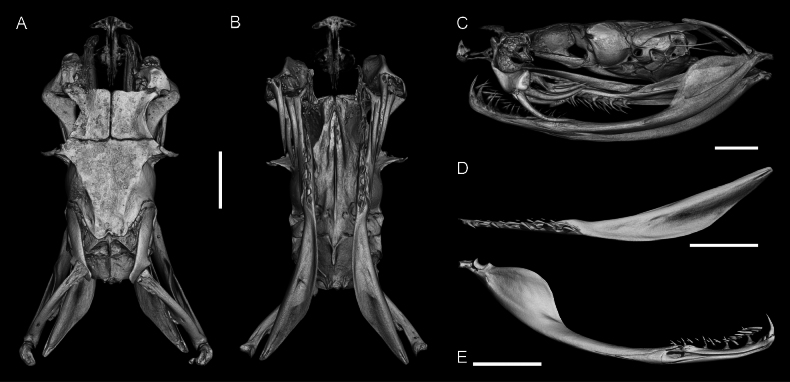
Skull of the adult male lectotype of *Gloydius himalayanus*BMNH 1946.1.19.64: **A**. Dorsal, **B**. Ventral (lower jaws virtually extracted) and **C**. Lateral view; **D**. Pterygoid virtually isolated, ventral view; **E**. Lower jaw virtually isolated, medial view. Scale bars: 4.0 mm.

***Lectotype***. • BMNH 1946.1.19.64, an adult male from "Garhval, Himalaya (altitude 9000 feet)" (Uttarkashi District, Uttarakhand State, India; see above for further discussion on the possible locality), collected by the brothers Adolf or Robert von Schlagintweit (Figs 6A, 12, 13). ***Paralectotype***. • BMNH 1946.1.18.75, an adult male, collector(s) not documented, ex Theodor Edward Cantor collection; other data as for the lectotype.

##### Description of the lectotype.

Adult male, indicated by the presence of hemipenes, in situ reaching level of 18^th^ subcaudal scale; 1/1 loreal, slightly higher than wide; nasal scale completely divided; 2/2 elongated preoculars, the lower one much narrower; 1/1 supraocular; 2/2 postoculars, upper scarcely reaching onto top of head, the lower can be described as postsubocular, because it extends below the eye, up to the level of the end of the third supralabial; rostral scale wider than long; two internasals much wider than long; two prefrontals, slightly longer than wide; frontal bell-shaped, 22% longer than wide; 3/3 anterior temporals upper two very small, and 3/3 posterior temporals of similar size, the lower one in contact with the second last supralabial; supralabials 7/7, second last twice as high as the previous one, in contact with lower posterior temporal, 3^rd^/3^rd^ in contact with the eye; pit opening as long as the horizontal diameter of the eye and encircled by 3/3 scales; 18 circum-pileus scales; 10/10 sublabials, first four in contact with anterior submaxillars; two pairs of submaxillars, anterior approx. twice as wide and 50% longer than posterior; followed by four rows of paired gular scales, slightly increasing in size posteriorly; dorsal scales in 21-21-17 rows, strongly keeled, except the outer row, which is smooth, paired apical pits on some dorsals, very weakly developed; four preventrals; 162 ventrals; cloacal plate entire; 50/51 paired subcaudal scales. Body compact, subcylindrical; tail short (TaL/SVL 0.145); SVL 537 mm; TaL 91 mm, head length measured from tip of snout to posterior border of parietals 17.5 mm, head length measured from tip of snout to posterior edge of mandible 28.5 mm, head width 16.4 mm.

##### Dorsal scale reduction formula.

**Table:** 

–	4+5(7)	4+5(13)	-5(108)	3+4(120)
**(5)27**------------------------**25**-------------------------**23**------------------------------**21**------------------------------**19**---------------------------**17(162)**.				
-6, -12(6)	4+5(7)	4+5(12)	5+6(111)	2+3(122)

##### Dentition.

Maxillary bone with two posteriorly curved fangs on each side. On left side, the lateral main fang is loose, both teeth are not connected to the maxilla on right side. Behind each main fang are 7/8 replacement fangs at different growth stages. Main fang 6.44 mm in length, i.e., 31.52% of skull length. Discharge orifice 0.97 mm in length, i.e., 15.06% of fang length.

Palatine bone with 3/3 posteriorly curved teeth slightly decreasing in size posteriorly. Teeth I and II loose on left, teeth I and III on right side. Lateral to each palatine tooth is a single replacement tooth at different growth stages.

Pterygoid bone with 9/9 posteriorly curved teeth, shorter than the palatine tooth, all nearly the same size. Teeth II, IV, VI and VIII loose on left side as well as on right side. The posterior ~64% of the pterygoid bone is without teeth.

Mandibular bone with 11/11 posteriorly curved teeth gradually decreasing in size posteriorly. The first two teeth closer together than the rest. Medial to each mandibular tooth are two replacement teeth in different growth stages. Teeth II, III, V, VII, IX and XI loose on left side. Teeth II, IV, VI and VIII loose on right side. Splenial 93% of length of angular, latter overlaps splenial with 8.19% of its total length. The complete skull of the lectotype BMNH 1946.1.19.64 is presented in Fig. 13.

##### Colouration and pattern.

**Figure 14.**
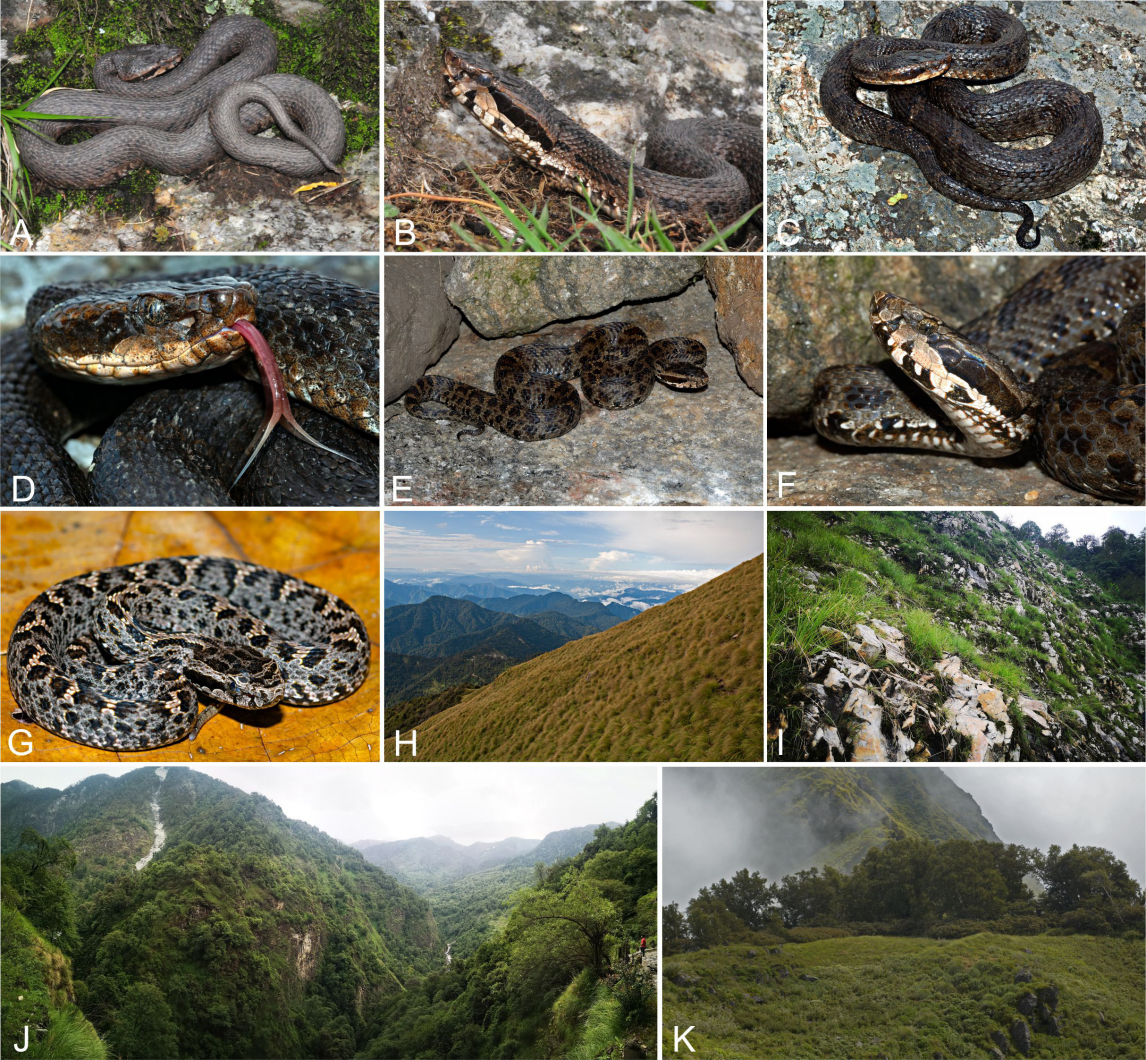
The colour and pattern in *Gloydius himalayanus* and its typical habitat. (**A, B**) Adult male (uncollected) from between Bhebra camp site and Manjhi (Baharat Range), Uttarkashi District, Uttarakhand State, India, 2400 m elevation (photo by Frank Tillack); (**C, D**) An uncollected specimen from Khaliya [Khulia] Danda, Pithoragarh District, Uttarakhand State, India, 3433 m elevation (photo by Ashok Captain and Emmanuel Theophilus); (**E, F**) An uncollected specimen from Dungair Dhar, Pithoragarh District, Uttarakhand State, India, 3535 m elevation (photo by Ashok Captain and Emmanuel Theophilus); **G**. Juvenile (uncollected) from Dhapa Bend, Pithoragarh District, Uttarakhand State, India, 1794 m elevation (photo by Ashok Captain and Emmanuel Theophilus); **H**. General view of the habitat of *Gloydius himalayanus* at Khaliya [Khulia] Danda, Pithoragarh District, Uttarakhand State, India, 3433 m elevation (photo by Ashok Captain and Emmanuel Theophilus); **I**. Microhabitat of *G. himalayanus* between Bhebra camp site and Manjhi (Baharat Range), Uttarkashi District, Uttarakhand State, India, 2400 m elevation; **J**. General view of the habitat of *G. himalayanus* between Bhebra camp site and Manjhi (Baharat Range), Uttarkashi District, Uttarakhand State, India, 2400 m elevation; **K**. Habitat of *G. himalayanus* at Dungair Dhar, Pithoragarh District, Uttarakhand State, India, 3535 m elevation (photo by Ashok Captain and Emmanuel Theophilus).

Colouration after ~170 years preservation in ~70% ethanol was recorded as follows: dorsal ground colour Light Neutral Gray (Colour 297), with 40 Pale Neutral Gray (296) irregular bands on body, one to two dorsal scales long and ten bands on tail, the anterior edge of dorsal bands shows thin Dark Grayish Brown (284) borders; all dorsal scales show a Dark Grayish Brown (284) mottling, which condenses dorsoventrally; dorsal and lateral head with same ground colour as dorsal body and densely mottled with Dark Grayish Brown (284); a wide Sepia (279) postocular stripe from the posterior border of the eye to the angle of the mouth, whose lower edge is bordered by a Dark Grayish Brown (284) wavy, narrow line; lower parts of posterior supralabials Pale Buff (1) and heavily mottled with Dark Grayish Brown (284); Venter with a colour gradient that becomes darker towards the back. Throat Pale Buff (1) coloured submaxillars and gulars mottled with Dark Grayish Brown (284); sublabials with an irregular Fawn Colour (258) pattern; in the further course, venter changes gradually from True Cinnamon (260) with heavy Dark Grayish Brown (284) mottling via Burnt Sienna (38) to Maroon (39); the dark ventral parts of posterior third of body and ventral tail are Pale Buff (1) mottled; tail tip Light Buff (2) coloured. Examples of variation in the colouration and pattern of live individuals; see Fig. 14.

##### Variation.

The paralectotype and additional examined material agree well with the lectotype in general appearance. For differences based on sexual dimorphism, morphometrics and scalation we refer to Tables 3, 4.

**Table 3.** Summary statistics of diagnostic meristic characters and morphological characters for males of the species of the *Gloydius himalayanus* complex. Highlighted character states are those that differ significantly (*p* < 0.05) from *G. himalayanus* (h), *G. hazarensis* sp. nov. (z), or *G. nepalensis* sp. nov. (n).

Males	*G. himalayanus*	*G. hazarensis* sp. nov.	*G. hindukushensis* sp. nov.	*G. nepalensis* sp. nov.
*Ven*				
*n*	16	6	1	13
Range (mean ± SD)	144–164 (**156.1**^n^ ± 5.1)	147–159 (153.5 ± 4.7)	**146**	140–158 (**147.9**^h^ ± 5.4)
*Ven+SubC*				
*n*	16	6	1	13
Range (mean ± SD)	192–218 (**205.3**^n^ ± 6.9)	195–211 (**203.2**^n^ ± 6.0)	**N/A**	**193.6** ^h,z^
*PrO*				
*n*	16	6	1	13
Range (mean ± SD)	2–3 (**2.1**^z^ ± 0.3)	2–3 (**2.8**^h,n^ ± 0.4)	**2**	**2 (2**^z^ ± 0)
*PrefW* (mm)				
*n*	16	7	1	12
Range (mean ± SD)	2.3–4.8 (**3.3**^n^ ± 0.7)	2.1–3.7 (**3.0**^n^ ± 0.6)	**2.32**	2.8–5.1 (**3.8**^h,z^ ± 0.6)
*INW* (mm)				
*n*	16	7	1	12
Range (mean ± SD)	1.5–4.0 (2.9 ± 0.7)	2.2–3.6 (**2.8**^n^ ± 0.5)	**-**	2.0–4.1 (**3.3**^z^ ± 0.6)

**Table 4.** Summary statistics of diagnostic meristic and morphological characters for females of the species of the *Gloydius himalayanus* complex. Highlighted character states are those that differ significantly (*p* < 0.05) from *G. himalayanus* (h), *G. hindukushensis* sp. nov. (u), *G. hazarensis* sp. nov. (z), or *G. nepalensis* sp. nov. (n).

Females	*G. himalayanus*	*G. hazarensis* sp. nov.	*G. hindukushensis* sp. nov.	*G. nepalensis* sp. nov.
*Ven*				
*n*	14	7	4	12
Range (mean ± SD)	150–164 (**158.9**^n,z^ ± 4.0)	158–165 (**162.4**^h,n^ ± 2.4)	147–159 (152.5 ± 5.5)	146–161 (**152.5**^h,z^ ± 5.1)
*Ven+SubC*				
*n*	14	7	3	12
Range (mean ± SD)	195–216 (**206**^n^*±* 6.9)	201–215 (**209.7**^n^*±* 4.7)	198–204 (200.3 *±* 3.2)	188–205 (**195.7**^h,z^*±* 5.7)
*SubL*				
*n*	14	7	4	12
Range (mean ± SD)	8–11 (**9.6**^n^*±* 0.8)	8–10 (**9.7**^n^*±* 0.8)	8–9 (8.8 *±* 0.5)	7–10 (**8.8**^h,z^*±* 0.8)
*PrO*				
*n*	14	7	4	12
Range (mean ± SD)	2–3 (**2.1**^z^*±* 0.4)	**3 (3**^n^*±* 0)	**3 (3**^n^*±* 0)	2 (**2**^u,z^*±* 0)
*DSR 3*				
*n*	14	7	4	12
Range (mean ± SD)	17 (**17**^u^*±* 0)	17 (17 *±* 0)	17–19 (**18**^h,n^*±* 1.2)	15–17 (**16.8**^u^*±* 0.6)
*PrefL* (*mm*)				
*n*	16	6	4	7
*Range* (mean ± SD)	1.5–4.6 (**3.5**^z^*±* 0.8)	2.0–3.7 (**2.7**^h^*±* 0.6)	1.8–4.16 (**2.7***±* 1.0)	3.1–3.7 (**3.3***±* 0.2)
*PrefW* (*mm*)				
*n*	16	6	4	7
Range (mean ± SD)	2.2–4.5 (**3.4**^u,z^*±* 0.5)	2.1–3.3 (**2.7**^h,n^*±* 0.5)	1.9–3.31 (**2.3**^h,n^*±* 0.7)	3.1–3.8 (**3.4**^u,z^*±* 0.3)
*INW* (*mm*)				
*n*	16	6	3	7
Range (mean ± SD)	2.3–3.9 (**3.1**^u,z^*±* 0.4)	1.3–2.3 (**1.7**^h^*±* 0.3)	1.5–1.73 (**1.6**^h^*±* 0.1)	2.3–3.2 (2.8 *±* 0.2)
*INL* (*mm*)				
*n*	16	6	3	7
Range (mean ± SD)	1.2–2.6 (**1.7**^u,z^*±* 0.3)	1.0–3.3 (**2.4**^h^*±* 1.0)	1.9–4.02 (**2.6**^h^*±* 1.2)	1.5–2.9 (2.0 *±* 0.5)
*INW.L* (*mm*)				
*n*	16	6	3	7
Range (mean ± SD)	1.4–2.8 (**1.9**^u,z^ ± 0.4)	0.5–2.3 (**0.9**^h^ ± 0.7)	0.43–0.8 (**0.7**^h^ ± 0.2)	0.8–1.8 (1.5 ± 0.4)

##### Sum formula of dorsal scale reduction in males.

**Table:** 

(6–14)	(6–23)	(90–110)	(103–130)
4+5, 6+7	4+5, -5, 5+6	3+4, 4+5, -5, 5+6	3+4, 4+5
**(5) 25 -------------------- 23 ---------------------------- 21 ------------------------------- 19 --------------------- 17**.			
4+5, 6+7	4+5, -5, 5+6	3+4, 4+5, 5+6	2+3, 3+4, 4+5
(6–13)	(6–25)	(85–113)	(106–137)

##### Sum formula of dorsal scale reduction in females.

**Table:** 

(6–11)	(11–26)	(90–107)	(103–117)
4+5, -5, 5+6, 6+7	4+5, 5+6	4+5, 5+6	3+4, 4+5
**(5) 25 ------------------------- 23 -------------------------- 21 ------------------------ 19 ----------------------- 17**.			
4+5, -5, 5+6, 6+7	3+4, 4+5, 5+6	3+4, 4+5, 5+6	3+4, 4+5
(6–11)	(10–24)	(92–105)	(105–117)

##### Variation in dentition.

Maxillary bone with two posteriorly curved fangs on each side. Main fang 3.89–6.44 mm in length, i.e., 23.95–31.52% of skull length. Discharge orifice 0.52–0.97 mm in length, i.e., 12.9–15.1% of fang length. Palatine bone with three posteriorly curved teeth slightly decreasing in size posteriorly. Pterygoid bone with 7–10 posteriorly curved teeth, shorter than the palatine tooth, all nearly the same size. The posterior 59.7–66.3% of the pterygoid bone is without teeth. Mandibular bone with 10–12 posteriorly curved teeth gradually decreasing in size posteriorly. The first two teeth closer together than the rest. Splenial either 93.0% of length of angular or fused. The total length of splenial-angular complex spans 22.9–29.0% of the mandibular bone. The dental is 36.1–39.3% as long as the mandibular bone.

##### Variation in life colouration and pattern.

Dorsal ground colour varies from Drab (Colour 19) to Cinnamon-Drab (50), Drab-Grey (256) or Lavender (202), with 29–40 Olive-Brown (278), Warm Sepia (40), or Dusky Brown (285) irregular bands on dorsal body. The bands can vary from one to three dorsal scales in length, can be interrupted vertebrally or can be broken in transverse rows of three roundish blotches. The anterior edge of the bands or blotches often shows a thin Dark Grayish Brown (284) border, whereas the upper row of blotches is separated by Pale Buff (1) coloured interspaces, especially in juveniles. All dorsal scales show a Dark Grayish Brown (284) mottling, which condenses dorsoventrally. Ventrolaterally, sometimes a small irregularly shaped Dusky Brown (285) spot, which is outlined with Smoky White (261), is present between the bands or the lower row of roundish blotches. Dorsal tail with 7–10 thin Olive-Brown (278) or Warm Sepia (40) coloured irregular bands. Tail tip lighter.

Dorsal head with same ground colour as dark body bands or blotches; canthus rostralis and upper temporal region usually lighter; neck usually with two paravertebral and two lateral Dark Grayish Brown (284) lines starting approximately at the level of the posterior edge of mandible and extending to the anterior border of first band or blotch; a wide Dusky Brown (285) postocular stripe runs from the posterior border of the eye to the angle of the mouth, whose lower edge is bordered by lighter Pale Buff (1) coloured wavy, narrow line, anterior part of postocular stripe covers more than two thirds of the anterior lower temporal scale; head sides including upper labials Pale Buff (1) coloured and heavily mottled with Dark Grayish Brown (284); venter with a colour gradient that becomes darker towards the tail. Throat Pale Buff (1) coloured, submaxillars and gulars mottled with Dark Grayish Brown (284); sublabials Pale Buff (1) coloured, sometimes with large irregular Dark Grayish Brown (284) blotches; in the further course, venter changes gradually from Pale Buff (1) via Pale Neutral Gray (296) or Drab-Gray (256) to Smoke Gray (268) or Olive brown (278) and is completely heavily mottled with Dark Grayish Brown (284).

##### Remarks on common and scientific name.

Wall (1910: 66) suggested the English common name "Brown Himalayan Viper", although we recommend using the more precise name "Brown Himalayan Pitviper". In Kashmir (where *G. chambensis* or *G. hazarensis* sp. nov. may also be present according to this study; Fig. 9), the pitviper is known as "pohur" by natives (Wall 1910). The epithet *himalayanus* has a long tradition in herpetology and is used as a reference to the species’ occurrence in the Himalayan region; it has been applied to several amphibians and reptiles from the area, e.g., *Duttaphrynus himalayanus* (Günther, 1864), *Tylototriton himalayanus* Khatiwada et al., 2015, *Cyrtopodion himalayanus* Duda & Sahi, 1978, *Ablepharus himalayanus* (Günther, 1864), *Rhabdophis himalayanus* (Günther, 1864) and *Protobothrops himalayanus* Pan et al., 2013.

##### Distribution.

Probably the first brief description of a venomous snake originating from the Himalaya that can be attributed to the *G. himalayanus* complex was given in a footnote in Vigne’s travelogue through Kashmir etc., published in 1842. Based on the results presented here (Fig. 9, Suppl. material 1: table SS3), this species is known exclusively from the Indian Himalaya. The westernmost localities include Dumas, Bairagarh in India, as reported by Kuttalam et al. (2022), while the easternmost locality is Lingurani (Captain, pers. comm. 2024). We estimate the polygonal range of the species to be ca 56,000 km^2^. However, we cannot exclude that *G. himalayanus* (sensu stricto) also occurs west of Himachal Pradesh e.g., in Kashmir. Therefore, until we have data to the contrary, we propose to list specimens from this transitional zone as *Gloydius cf. himalayanus*.

##### Habitat and ecology.

According to our data, the species inhabits elevations between ca 1000 and 3500 m in subtropical montane to subalpine zone (with a dubious record of ~4877 m introduced by Sclater (1891), see comment above on the distribution of the *G. himalayanus* complex) in northwestern India. The species prefers montane environments and is typically associated with coniferous forests, rocky slopes, alpine meadows, and areas with abundant leaf litter with cover such as logs, stones, and crevices (Manhas 2020; although the species affiliation of this population is unclear; see Fig. 14 and Suppl. material 1: table SS3). It exhibits a generalist microhabitat use but relies on thermally buffered refugia. Early reports from Kashmir, e.g., Alcock (1898), Wall (1899), Fenton (1910), and Dattatri (1985) described this snake as "very common to swarming", especially in the Liddar River Valley where it could be found almost everywhere. In Uttarakhand it is widely distributed and not difficult to find in suitable habitats in the Dehradun and Uttarkashi districts. Observations indicate that its presence is more frequent within protected areas, although the species can also occur near human settlements where the habitat is suitable.

##### Conservation.

*Gloydius himalayanus* is currently listed as Least Concern (LC) on the IUCN Red List (Limbu and Ghosh 2022), primarily due to its presumed wide distribution across the Southern Himalaya and its presence in multiple countries and protected areas. However, the taxonomic revision presented here significantly narrows the species’ confirmed range, restricting it to India. Consequently, a reassessment of its global conservation status is necessary. Although *G. himalayanus* is not listed in the CITES Appendices, it is legally protected in India under Schedule II of the Wildlife (Protection) Act, 1972, amended 2022. Despite its previous broad classification, the species faces several regional threats, including persecution (often fuelled by fear of snakebites), road mortality linked to expanding infrastructure, and habitat degradation resulting from agricultural activities and tourism-related construction. Pushpangadan et al. (2014) report that in some northwestern Indian rural communities, dead specimens or their body parts (e.g., fat and venom) are used for food or traditional medicines. Climate change may also pose an additional future threat by altering high-elevation habitats, although the extent of its impact remains unclear. While no species-specific conservation measures are currently in place, *G. himalayanus* benefits from some in-situ protection within Himalayan protected areas (e.g., Prasad et al. 2021).

#### Gloydius hindukushensis sp. nov.

Taxon classification

Animalia

SquamataViperidae

78DE78E2-577A-5E2F-95AF-F27D63FED0F2

https://zoobank.org/FD61634B-A2C3-4548-B1E2-DA3A0F39DB0C

##### Common names.

Hindu Kush Pitviper (English), ہندوکش گھڑے والا سانپ (Hindu Kush gaday wali afee, Urdu)

##### Type material.

**Figure 15.**
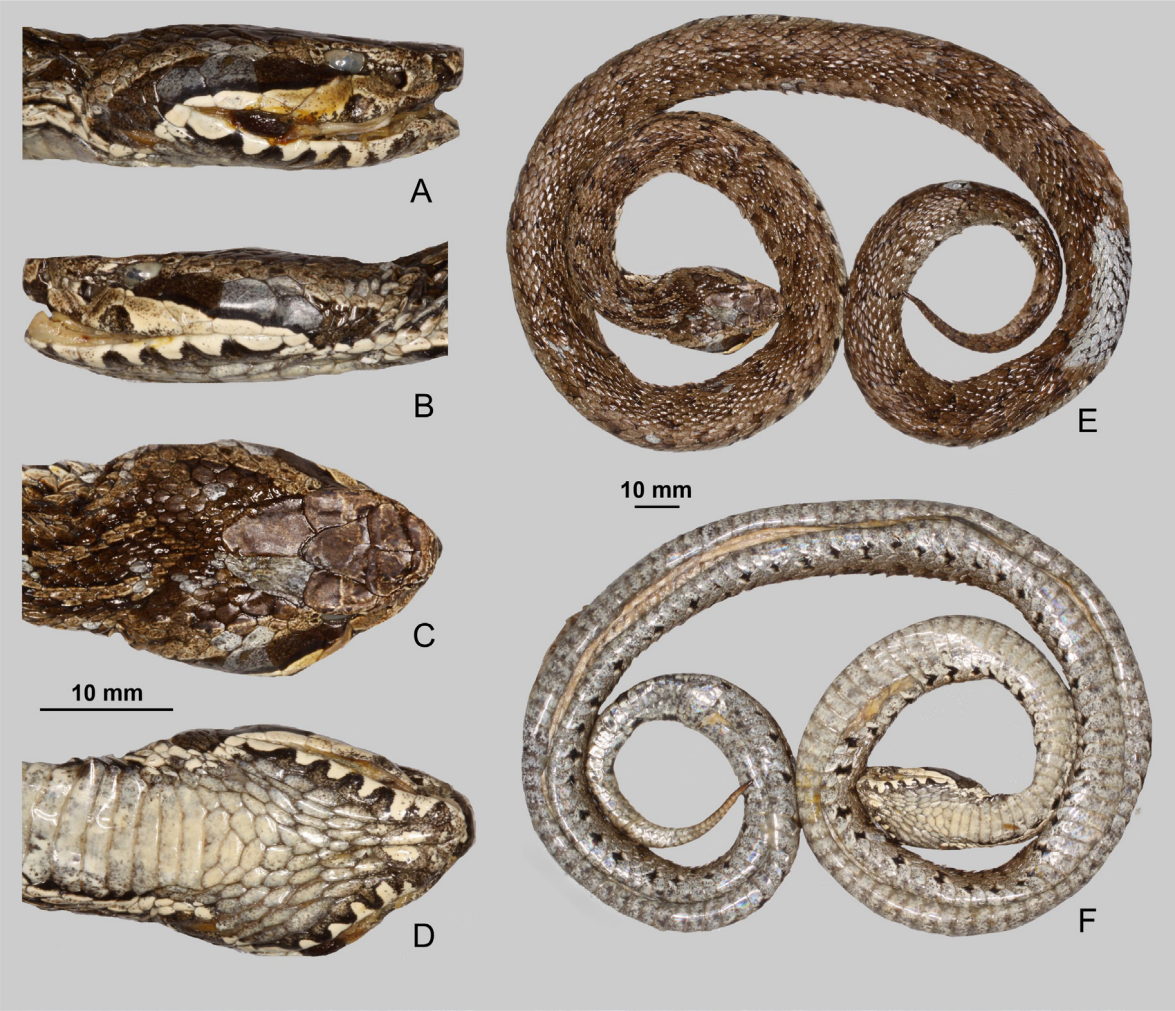
The adult female holotype of *Gloydius hindukushensis* sp. nov. (NHMW 41993). **A–D**. Lateral, dorsal and ventral views of the head; (**E, F**) dorsal and ventral views of the body.

***Holotype***. • NHMW 41993, an adult female from Kumrat Valley, Upper Dir District, Khyber Pakhtunkhwa Province, Pakistan, 35.5649°N, 72.1958°E, 2360 m altitude, found freshly killed by locals on 17 September 2020 by Daniel Jablonski (formerly CUHC 10085; Fig. 15). ***Paratype***. • CUHC 10088, a juvenile female, collected on 17 September 2020 by Daniel Jablonski, other data as for the holotype.

##### Description of the holotype.

Adult female, indicated by the absence of hemipenes; 1/1 loreal, slightly wider than high; nasal partially divided below naris; 3/3 elongated preoculars, the lowest is the narrowest; 1/1 supraocular; 3/3 postoculars, upper reaching onto top of head and touching the parietal, the lower can be described as postsubocular, because it extends under the eye, up to the level of the posterior border of the third supralabial; rostral scale wider than high; two internasals wider than long; two prefrontals, slightly longer than wide; frontal bell-shaped, as long as wide; 3/3 anterior temporals, upper two significantly smaller than the lower one, and 3/3 posterior temporals, the two upper of similar size, the lower one slightly larger and in contact with the second last supralabial; supralabials 7/7, second last three times higher than the previous one, in contact with lower posterior temporal, 3^rd^/3^rd^ in contact with the eye; pit opening shorter than horizontal diameter of eye and encircled by 3/3 scales; 18 circum-pileus scales; 9/9 sublabials, first four in contact with anterior submaxillars; two pairs of submaxillars, anterior nearly twice as wide and 80% longer than posterior; followed by five rows of paired gular scales, increasing in size posteriorly; dorsal scales in 23-21-19 rows, strongly keeled, except the outer row, which is only very slightly keeled, paired apical pits on some dorsals, very weakly developed; four preventrals; 155 ventrals; cloacal plate entire; 38/39 paired subcaudal scales. Body compact, subcylindrical; tail short (TaL/TL 0.122); SVL 578 mm; TaL 80 mm, head length measured from tip of snout to posterior border of parietals 17.9 mm, head length measured from tip of snout to posterior edge of mandible 27.0 mm, head width 15.9 mm.

##### Dorsal scale reduction formula.

**Table:** 

-4(6)	4+5(12)	4+5(101)	4+5(121)
(5)**24** ---------------------------- **23** --------------------------------------- **21** ----------------------------------- **19** -------------------------------- **17**(155).			
–	4+5(12)	4+5(100)	5+6(118)

##### Dentition.

**Figure 16.**
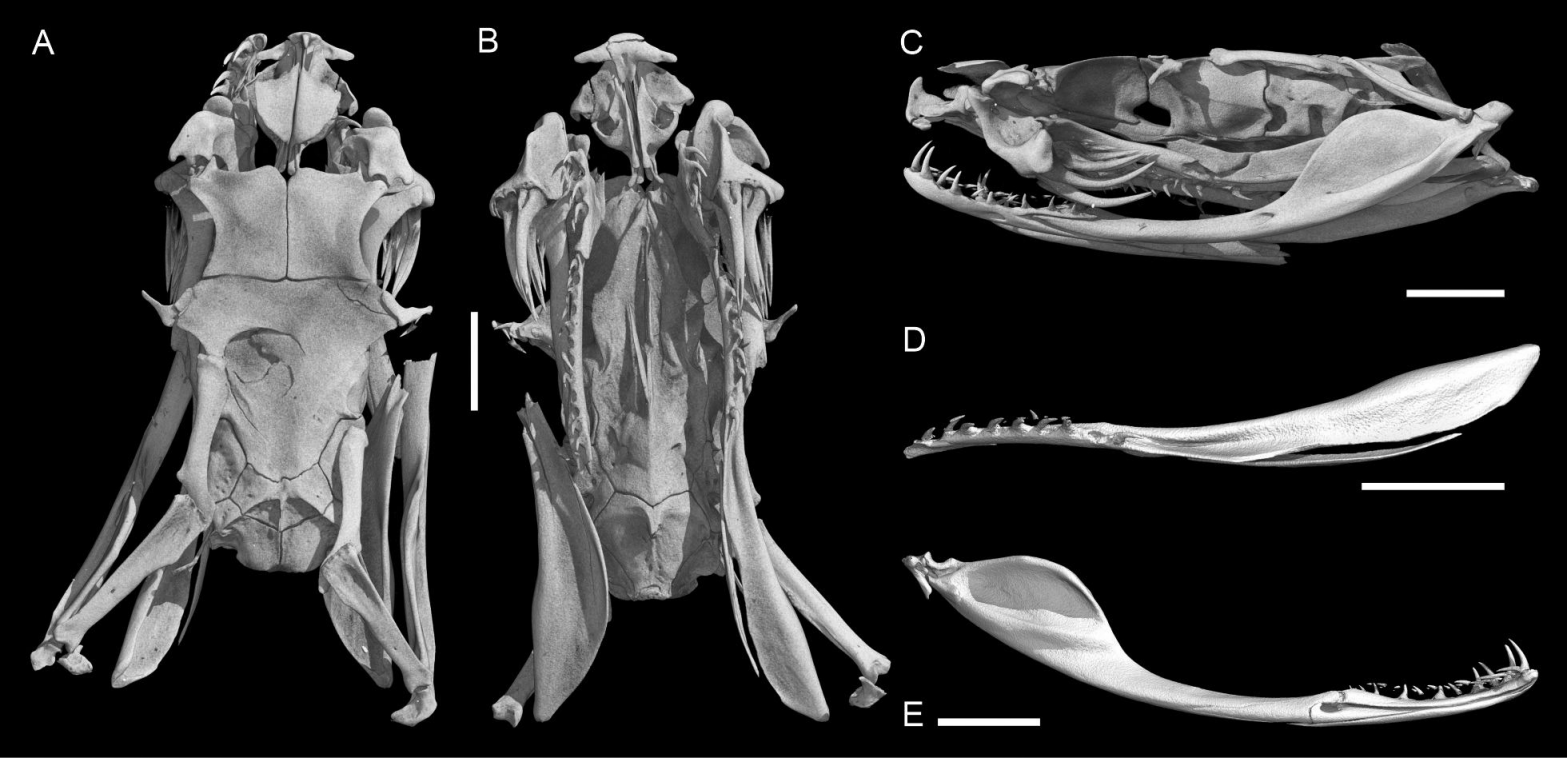
Skull of the adult female holotype of *Gloydius hindukushensis* sp. nov., NHMW 41993: **A**. Dorsal, **B**. Ventral (lower jaws virtually extracted) and **C**. Lateral view; **D**. Pterygoid virtually isolated, ventral view; **E**. Lower jaw virtually isolated, medial view. Scale bar: 4.0 mm.

Maxillary bone with two posteriorly curved fangs on each side. Both teeth are not connected to the maxilla on the right side. Behind the main fangs are 6/7 replacement fangs at different growth stages. Main fang 5.27 mm in length, i.e., 25.5% of skull length. Discharge orifice 1.14 mm in length, i.e., 21.6% of fang length. Palatine bone with 4/4 posteriorly curved teeth slightly decreasing in size posteriorly. Teeth III and IV loose on left, teeth II and IV on right side. Lateral to each palatine tooth is a single replacement tooth at different growth stages. Pterygoid bone with 10/12 posteriorly curved teeth, shorter than the palatine tooth, all nearly the same size. Tooth IX loose on left, teeth II, IV, VI, VII, IX and XII loose on right side. Pterygoid bone broken between tooth III and IV on left, as well as behind the tooth row on right side. The posterior 75.0% of the pterygoid bone is without teeth. Mandibular bone with 14/14 posteriorly curved teeth gradually decreasing in size posteriorly. The first two teeth closer together than the rest. Medial to each mandibular tooth are up to two replacement teeth in different growth stages. Teeth I, IV, VI, VIII, X, XII, and XIV loose on left side. Teeth I, III, V, VII, VIII, X, XI, and XIII loose on right side. Splenial 67.2% of length of angular, latter overlaps splenial with 18.3% of its total length. The total length of splenial-angular complex spans 33.4% of the mandibular bone. The dental is 38.5% as long as the mandibular bone. The complete skull of the holotype NHMW 41993 is presented in Fig. 16.

##### Colouration and pattern.

**Figure 17.**
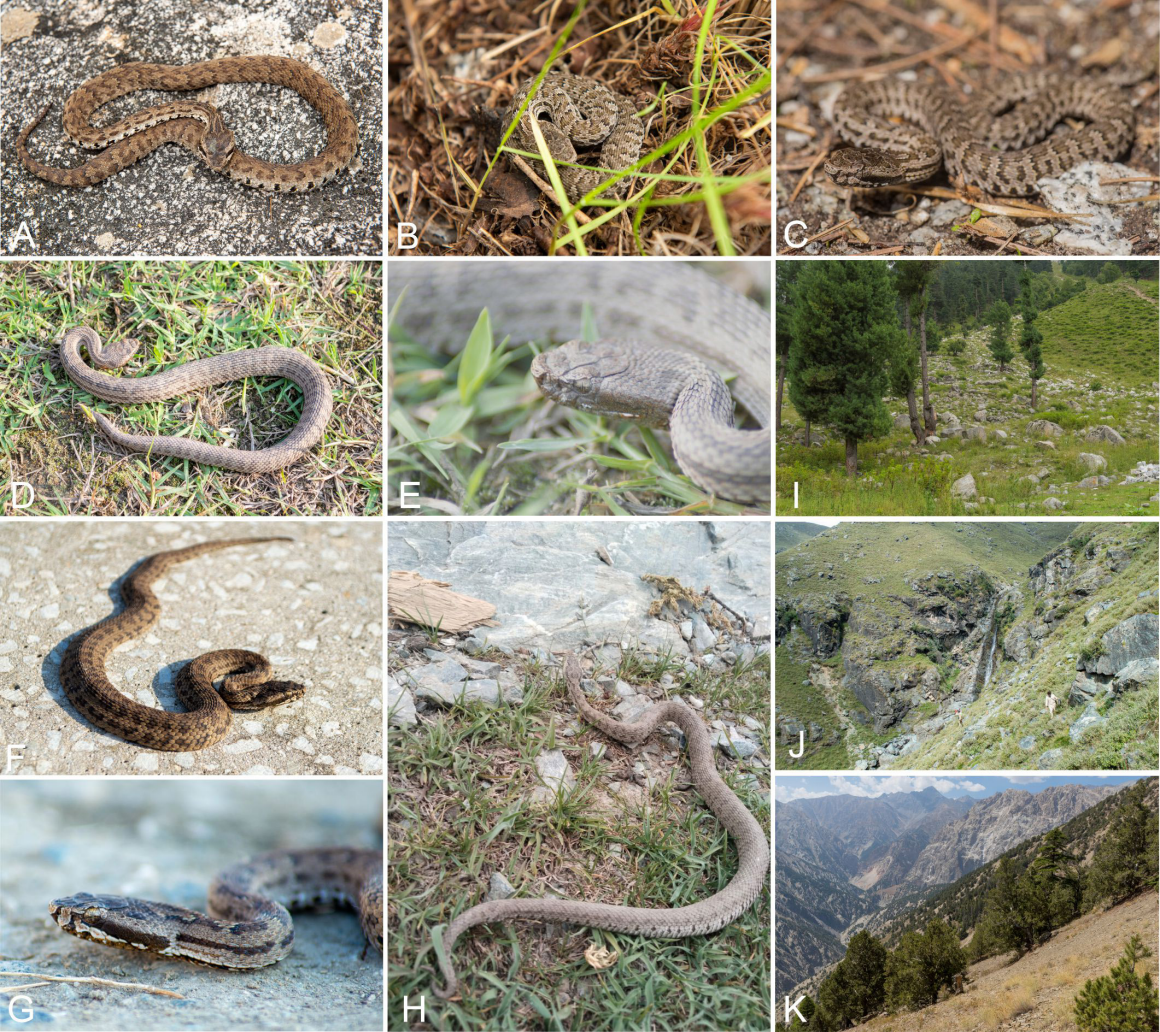
The colour and pattern in *Gloydius hindukushensis* sp. nov. and its typical habitat. **A**. Freshly killed holotype (NHMW 41993) from Kumrat Valley, Khyber Pakhtunkhwa, Pakistan; (**B, C**) The paratype (CUHC 10088) from Kumrat Valley, Khyber Pakhtunkhwa, Pakistan, all photos by Daniel Jablonski; (**D, E**) The specimen PMNH 5150 collected on 30 April 2023 from Lal Qilla, Lower Dir, Khyber Pakhtunkhwa, Pakistan; (**F, G**) The specimen PMNH 5149 collected on 16 October 2023 from Lal Qilla, Lower Dir, Khyber Pakhtunkhwa, Pakistan, photos by Muhammad Idrees; **H**. The specimens (uncollected) observed on 28 May 2025 from Broon area, Bomborate, Kalash Valley, Chitral, Khyber Pakhtunkhwa, Pakistan, photo by Abdul Basit; **I**. The habitat of the type locality of *G. hindukushensis***sp. nov**. in Kumrat Valley, photo by Daniel Jablonski; **J**. The habitat in Lal Qilla, photo by Muhammad Idrees; **K**. The habitat of the species in Chitral Gol National Park, Chitral, Pakistan, photo by Daniel Jablonski.

Colouration after approximately five years preservation in ~70% ethanol was recorded as follows: Dorsal ground colour Drab-Gray (Colour 256), with 37 Sepia (279) coloured dorsolateral roundish blotches which are four to five dorsal scales wide and show a pale Drab-Gray (256) centre; most blotches open ventrolaterally or the lower outer edges disintegrate and form a ventrolateral row of small spots; below that a row of small, irregular rhombic or x-shaped Sepia (279) coloured and Cream White (52) bordered spots runs along the outer edge of the ventrals; the dorsolateral blotches merge vertebrally and form an irregular, sometimes interrupted zigzag line; dorsal tail with ten irregular blotches in the same colouration as on the body; dorsal head Sepia (279) coloured; lateral head colour Pale Buff (1) to Cream White (52) with dense Sepia (279) mottling, most intense in the loreal and upper temporal region; a wide Olive-Brown (278) postocular stripe from the posterior border of the eye to the posterior edge of the mandible, whose lower edge is bordered by a dark Sepia (279), wavy narrow line; lower parts of posterior supralabials Pale Buff (1) with a few Dark Grayish Brown (284) speckles; lower labials with small triangle shaped Sepia (279) coloured markings on their sutures; neck with two wavy lateral Sepia (279) stripes and a shorter elongated spot in the centre; venter with a colour gradient that becomes darker towards the tail; throat Pale Neutral Gray (296) coloured, submaxillars and gulars mottled with Dark Grayish Brown (284); in the further course, venter ground colour changes gradually to Light Neutral Gray (297) and shows heavy Sepia (279) mottling; tail tip Medium Fawn (258) coloured. Examples of variation in the colouration and pattern of live individuals; see Fig. 17.

##### Variation.

The paratype and other examined material agree well with the holotype in general appearance. For differences based on sexual dimorphism, morphometrics and scalation we refer to Tables 3, 4, 6.

##### Sum formula of dorsal scale reduction in females.

**Table:** 

(12–13)	(84–101)	(107–121)
4+5	4+5, 5+6	4+5
**(10)23 ------------------------ 21 ---------------------------------------- 19 -------------------------------- 17**.		
4+5	4+5, 5+6	4+5, 5+6
(11–12)	(83–100)	(107–118)

Variation in dentition unknown.

##### Variation in life colouration and pattern.

Dorsal ground colour varies from Drab-Gray (Colour 256) to Medium Fawn (257) with 37–44 Ground Cinnamon (270) to Olive-Brown (278) coloured dorsolateral blotches on body and 10–12 on tail; the lighter centres of the body blotches are set off from the darker edge with fine white speckles.

##### Etymology.

The specific epithet hindukushensis is derived from the Hindu Kush mountain range, where the new species was discovered. The name is a Latinised adjective meaning "from the Hindu Kush". The term is of Persian origin, with a debated etymology that reflects the region’s role as a barrier between Central and South Asia. The Hindu Kush mountain range, spanning Afghanistan and Pakistan, forms a major biogeographic divide, separating distinct faunas on its eastern and western slopes. This pattern aligns with our findings of a distinct lineage within the *G. himalayanus* complex, whose distribution is shaped by mountain ridges and river valleys. The new species is distributed across the Pakistani portion of the Hindu Kush west of the Indus River. This study represents the first application of this epithet to a reptile species and highlights the region’s ecological and evolutionary distinctiveness, which supports other range-restricted or endemic species, such as *Paradactylodon mustersi* (Smith, 1940), *Chrysopaa sternosignata* (Murray, 1885), *Altiphylax levitoni* (Golubev & Szczerbak, 1979), or *Laudakia nuristanica* (Anderson & Leviton, 1969).

##### Distribution.

*Gloydius hindukushensis* sp. nov. is only known from a few localities between the border with Afghanistan and the Indus River in the south-eastern and eastern foothills of the Hindu Kush Mountains in Pakistan (own data and Sclater 1891; McMahon 1899; Wall 1911; Minton 1966; Gloyd and Conant 1990; Jamal et al. 2018). A combination of genetic and morphological data confirms the species from several localities in the Pakistani districts of Chitral, Swat, Upper and Lower Dir in the Khyber Pakhtunkhwa Province and from the districts of Gilgit and Diamer in the Gilgit-Baltistan Province (see Fig. 9, Suppl. material 1: table SS3).

These distribution points suggest a range confined to the eastern slopes of the Hindu Kush, and while the species’ presence in eastern Afghanistan remains unconfirmed, it has been previously considered likely (Gloyd and Conant 1990). The estimated extent of occurrence (EOO) is ca 31,000 km^2^, although actual occupancy is expected to be smaller due to limited known localities and the species’ apparent geographical isolation.

##### Habitat and ecology.

Wall (1911) described the snake (classified as *Ancistrodon himalayanus*) as common at Madak Lasht in Chitral where he found numerous specimens on the ground between rocks where they hide in beds of needles from various conifers e.g., *Picea morinda*, *Abies webbiana* and *Cedrus deodara*. Minton (1966) reported the Hindu Kush Pitviper (classified as *Agkistrodon himalayanus*) as plentiful at Liakot village (Swat) where it was found on rocky lightly wooded hillsides. Recently, verified locality records place this species at elevations ranging from approximately 1660 m (Torwal, Swat District, Pakistan) to 2888 m (Dog Dara, Upper Dir District, Pakistan). Other significant localities include the type locality in the Kumrat Valley (2360 m) and areas near Lal Qilla (2446–2700 m). This elevational range situates *G. hindukushensis* sp. nov. within montane coniferous and mixed forest zones, encompassing temperate pine forests, alpine scrub, and open rocky terrain interspersed with grassy cover and scattered shrubs (Fig. 17). These habitats are characterised by cool, humid conditions, with heavy snowfall in winter and a monsoon-influenced wet season, providing a suitable microclimate and ecological niche for this high-altitude viper.

##### Conservation.

An early account by McMahon (1899), referring to the species under the name *Halys himlayanus*, noted its abundance in the wooded tracts of the Gilgit District. However, its restricted geographic distribution in the Pakistani part of the Hindu Kush, high-altitude ecological specialisation, and apparent habitat specificity suggest that the species may be particularly susceptible to the impacts of climate change, anthropogenic habitat alteration, and possible range contraction. In Pakistan, populations historically attributed to *G. himalayanus* are frequently persecuted and killed by local communities due to fear or a lack of basic knowledge of snake behaviour, a threat that also led to the death of the holotype specimen described in this study. These combined pressures underscore the need for targeted conservation measures and systematic field surveys to evaluate population status, distribution boundaries, and prevailing threats.

#### Gloydius hazarensis sp. nov.

Taxon classification

Animalia

SquamataViperidae

B6B9CCFF-7603-5A5D-80AD-6567D371F49E

https://zoobank.org/07A9C4B5-6D4A-4D89-AE12-3298D4D0CFDA

##### Proposed common names.

Hazara Pitviper (English), हज़ारा पिट वाइपर (Hazārā Piṭ Vāipar; Hindi) and ہزارہ گڑھے والا سانپ (Hazara gaday wali afee; Urdu).

##### Type material.

**Figure 18.**
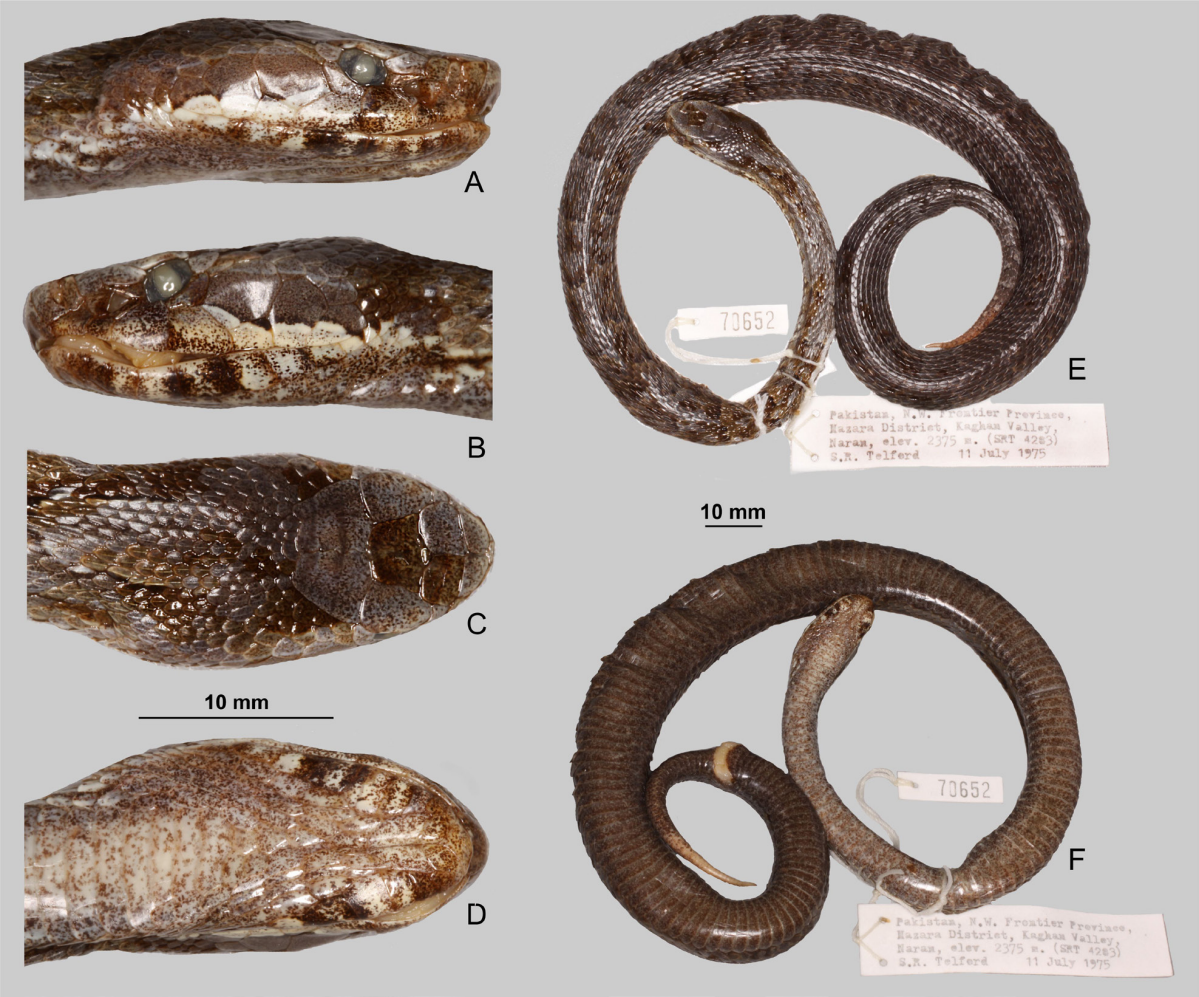
The adult female holotype of *Gloydius hazarensis* sp. nov. (UF 70652). **A–D**. Lateral, dorsal and ventral views of the head; (**E, F**) dorsal and ventral views of the body.

***Holotype***. • UF 70652 (field no. SRT 4283), an adult female from Naran town, Mansehra District, Khyber Pakhtunkhwa, Pakistan, 34.9061°N, 73.6494°E, 2375 m altitude, collected on 11 July 1975 by Sam Rountree Telford Jr. (Fig. 18). ***Paratypes***. • NHMW 17078:1, adult male from Murree, Murree District, Punjab Province, collected in 1878 by the Moravian-Austrian zoologist Ferdinand Stoliczka; UF 70656–70657 (field nos. SRT 5162–5163), adult females, other data as for the holotype; UF 82634, adult female from Nathia Gali, Abbottabad District, Khyber Pakhtunkhwa, Pakistan, 34°04'N, 73°23'E, ~2400 m altitude, collected on 14 July 1986 by Walter Auffenberg.

##### Description of the holotype.

Adult female, indicated by the absence of hemipenes; 1/1 loreal, as high as wide; nasal partially divided below naris; 3/3 elongated preoculars, the lowest is the narrowest; 1/1 supraocular; 2/2 postoculars, upper reaching onto top of head and touching the parietal, the lower can be described as postsubocular, because it extends under the eye, up to the level of the posterior border of the third supralabial; rostral scale wider than high; two internasals wider than long; two prefrontals, wider than long; a small oval scale between posterior end of prefrontal suture and frontal; frontal trapezoidal, as long as wide, anterior edge wider than posterior; 2/3 anterior temporals, upper significantly smaller than the lower one, and 3/2 posterior temporals, the lower one slightly larger than upper and in contact with the second last supralabial; supralabials 7/7, second last two and a half times higher than the previous one, in contact with lower posterior temporal, 3^rd^/3^rd^ in contact with the eye; pit opening shorter than horizontal diameter of eye and encircled by 3/3 scales; 15 circum-pileus scales; 10/10 sublabials, first four in contact with anterior submaxillars; two pairs of submaxillars, anterior nearly twice as wide and 50% longer than posterior; followed by four rows of paired gular scales, increasing in size posteriorly; dorsal scales in 21-20-17 rows, strongly keeled, except the outer row, which is only very slightly keeled, paired apical pits on some dorsals, very weakly developed; three preventrals; 163 ventrals; cloacal plate entire; 40/41 paired subcaudal scales. Body compact, subcylindrical; tail short (TaL/TL 0.123); SVL 400 mm; TaL 56 mm, head length measured from tip of snout to posterior border of parietals 13.7 mm, head length measured from tip of snout to posterior edge of mandible 20.3 mm, head width 11.3 mm.

##### Dorsal scale reduction formula.

**Table:** 

5+6(9)	4+5(80)	+5(86)	-5(89)	3+4(105)
(5)**23** ------------------ **21** ------------------------------- **20** ----------------------- 2**1** --------------------------- **19** ----------------------------- **17**(163).				
3+4(8)	–	–	5+6(96)	3+4(106)

##### Dentition.

**Figure 19.**
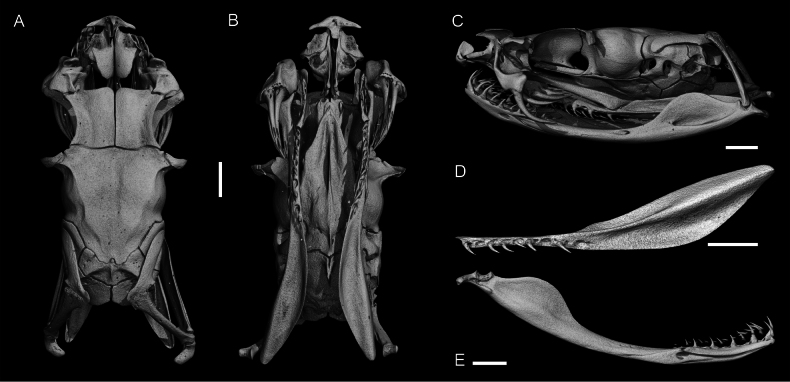
Skull of the adult female holotype of *Gloydius hazarensis* sp. nov., UF 70652: **A**. Dorsal, **B**. Ventral (lower jaws virtually extracted) and **C**. Lateral view; **D**. Pterygoid virtually isolated, ventral view; **E**. Lower jaw virtually isolated, medial view. Scale bar: 2.0 mm.

Maxillary bone with two posteriorly curved fangs on each side. On both sides the medial fang is not connected to the maxilla. Behind the main fangs are 5/6 replacement fangs at different growth stages. Main fang 3.98 mm in length, i.e., 24.1% of skull length. Discharge orifice 0.76 mm in length, i.e., 19.1% of fang length. Palatine bone with 4/3 posteriorly curved teeth slightly decreasing in size posteriorly. Teeth one and three loose on left, all teeth loose on right side. Lateral to each palatine tooth is a single replacement tooth at different growth stages. Pterygoid bone with 9/9 posteriorly curved teeth, shorter than the palatine tooth, all nearly the same size. Teeth II, V, VII and IX loose on left side. Teeth I, II, IV, VI and VIII loose on right side. The posterior 60.8% of the pterygoid bone is without teeth. Mandibular bone with 12/12 posteriorly curved teeth gradually decreasing in size posteriorly. The first two teeth closer together than the rest. Medial to each mandibular tooth are up to two replacement teeth in different growth stages. Teeth I, III, V, VII, IX, XI and XII loose on left side. Teeth I, III, V, VII, IX and XI loose on right side. Splenial and angular bones are fused. The total length of splenial-angular complex spans 27.0% of the mandibular bone. The dental is 36.4% as long as the mandibular bone. The complete skull of the holotype UF 70652 is presented in Fig. 19.

##### Colouration and pattern.

**Figure 20.**
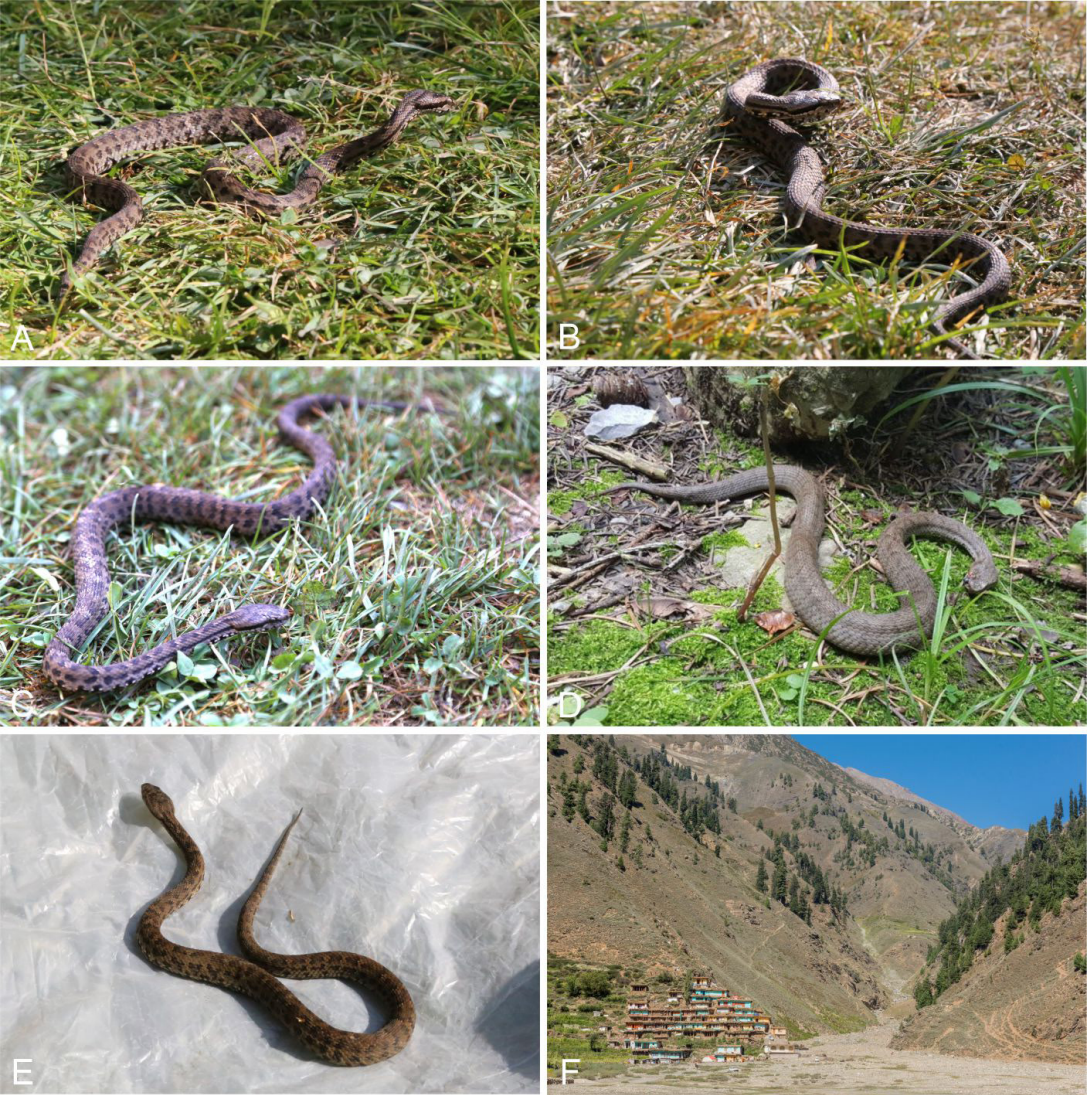
The colour and pattern in *Gloydius hazarensis* sp. nov. and its typical habitat. **A–E**. Adult specimens from Naran Valley, Khyber Pakhtunkhwa, Pakistan (photos by Rafaqat Masroor) and the habitat of the type locality of the species near Naran town (F; photo by Daniel Jablonski).

Colouration after ~50 years preservation in ~70% ethanol was recorded as follows: dorsal ground colour Pale Neutral Gray (Colour 296), with 34 Olive-Brown (278) coloured dorsolateral roundish blotches that are 4–6 dorsal scales wide and show a light Pale Neutral Gray (296) centre; most blotches open vertebrally and ventrolaterally or the lower outer edges disintegrate and form a ventrolateral row of small spots; below that a row of small, irregular Olive-Brown (278) coloured spots runs along the outer edge of the ventrals; dorsal tail with five irregular blotches in the same colouration as on the body; dorsal head Pale Neutral Gray (296) coloured and densely Olive-Brown (278) mottled; loreal and upper temporal region of head Pale Neutral Gray (296) with dense Olive-Brown (278) mottling; a wide Light Neutral Gray (297) postocular stripe from the posterior border of the eye to the posterior edge of the mandible, whose lower edge is bordered by a Dark Grayish Brown (284), wavy narrow line; lower parts of posterior supralabials Cream White (52) with Sepia (279) speckles; lower labials with Sepia (279) coloured markings on their sutures; venter with a colour gradient that becomes darker towards the tail; throat Pale Neutral Gray (296) coloured with dense Sepia (279) mottling; in the further course, venter ground colour changes gradually to Medium Neutral Gray (298) and shows a very dense Sepia (279) mottling; tail tip Drab-Gray (256) coloured. Examples of variation in the colouration and pattern of live individuals; see Fig. 20.

##### Variation.

The paratypes and other examined material agree well with the holotype in general appearance. For differences based on sexual dimorphism, morphometrics and scalation we refer to Tables 3, 4, 7.

##### Sum formula of dorsal scale reduction in males.

**Table:** 

(12–14)	(60–69)	(111–113)
4+5, 5+6	4+5, 6+7	3+4, 6+7
**(10) 23 --------------------------- 21 --------------------------------- 19 --------------------------------------- 17**.		
3+4, 5+6	4+5, 5+6	3+4, 5+6
(11–13)	(51–69)	(112–115)

##### Sum formula of dorsal scale reduction in females.

**Table:** 

(6–16)	(79–111)	(105–127)
-5, 5+6	-4, 4+5,-5	3+4, 3+5
**(10) 23 ------------------------------ 21 ------------------------------------- 19 -------------------------------- 17**.		
3+4, -5, 5+6	-5, 5+6	3+4, 4+5
(6–16)	(86–109)	(106–127)

##### Variation in dentition.

Maxillary bone with two posteriorly curved fangs on each side. Main fang 3.98–5.99 mm in length, i.e., 24.1–29.2% of skull length. Discharge orifice 0.76–1.17 mm in length, i.e., 14.7–22.3% of fang length. Palatine bone with 3–5 posteriorly curved teeth slightly decreasing in size posteriorly. Pterygoid bone with 8–11 posteriorly curved teeth, shorter than the palatine tooth, all nearly the same size. The posterior 56.3–61.6% of the pterygoid bone is without teeth. Mandibular bone with 11 or 12 posteriorly curved teeth gradually decreasing in size posteriorly. The first two teeth closer together than the rest. Splenial either 94.3% of length of angular or fused. The total length of splenial-angular complex spans 24.2–27.2% of the mandibular bone. The dental is 34.6–37.3% as long as the mandibular bone.

##### Variation in colouration and pattern.

Dorsal ground colour can darken considerably into Sepia (279) during preservation, so that the dorsal pattern elements of the head and body are no longer recognisable; number of dorsolateral blotches can vary from 29–34 on body and from 5–7 on tail.

##### Etymology.

The specific epithet *hazarensis* is a latinised adjective referring to the Hazara region of northeastern Pakistan, where the new species was discovered, where its type locality lies, and where it is currently known to occur. This region lies between the Indus River to the west, the foothills of the western Himalaya to the east, and the Karakoram to the north, which together create natural barriers. It includes the Pakistani districts of Abbottabad, Mansehra, Haripur, Battagram, Torghar, Kohistan (east of the Indus River) in Khyber Pakhtunkhwa Province, and is characterised by rugged montane landscapes, river valleys, and rich temperate forests. The name highlights the geographic origin of the species and reflects the ecological distinctiveness of this transitional zone between the Indo-Gangetic Plains and the western Himalayan biodiversity corridor (Khan 2006). The term should not be confused with the ethnically defined "Hazara" region of central Afghanistan (Hindu Kush), as the two are geographically and culturally distinct. The name was previously used for example for the endemic frog of the region, Rana (Paa) hazarensis Dubois & Khan, 1979 (today *Nanorana hazarensis*) or for an invalid subspecies of the Black-spined toad, *Bufo melanostictus hazarensis* Khan, 2001, today in the synonymy of *Duttaphrynus melanostictus* (Schneider, 1799).

##### Distribution.

According to the data presented here, the western range limit of the species is likely defined by the Upper Indus Valley, which separates the Hindu Kush from the western Himalaya (Fig. 9). The northern boundary is probably formed by the Karakoram Range, while the southern limit appears to be the Indus Plains, south of Murree in Punjab Province, Pakistan. The eastern distribution remains unclear. *Gloydius hazarensis* sp. nov. is so far only reported from northern Pakistan. Records are known from the districts of Kolai Palas, Mansehra, and Abbottabad in the Khyber Pakhtunkhwa Province, from the districts of Murree and Rawalpindi in the Punjab Province and from the districts of Neelam, Poonch and Bagh of the Azad Jammu and Kashmir, Pakistan-administered region (own data and Blanford 1878; Unwin in Lawrence 1895; Fenton 1910; Telford 1980; Khan and Tasnim 1986; Gloyd and Conant 1990; Showler 1998; Masroor 2017; Shabir 2022; Hadi and Junaid 2024; see also Fig. 9 and Suppl. material 1: table SS3). The species is likely present around the Kashmir Valley; however, more extensive sampling and DNA barcoding are required to delineate the range limits between *G. hazarensis* sp. nov. and *G. chambensis* in the area. We estimate the total range of the species to be ca 27,500 km^2^.

##### Habitat and ecology.

Little is known about the habitat and ecology of the species, as it was previously classified under *G. himalayanus* (see Khan and Tasnim 1986; Khan 2006; Masroor 2012). Based on verified locality data, the species occurs at elevations ranging from approximately 1630 m to 2900 m. This elevational range places *G. hazarensis* sp. nov. within montane to subalpine ecological zones, where climatic conditions are generally cool to cold, with seasonal snowfall and a pronounced monsoon period (see Fig. 20). For example, in Murree hills and surrounding Himalayan foothill regions of Punjab, Pakistan it can be found in moderate to steep slopes, rocky terrain, and a mosaic of forest patches interspersed with grassy clearings and human-altered landscapes in the subtropical pine forest, found between 1000–2000 m. Such habitat is characterised by *Pinus roxburghii*, which forms the dominant canopy, with an understory comprising *Dodonaea viscosa*, *Carissa opaca*, and various grasses, e.g., *Cymbopogon jwarancusa*. At higher elevations (up to ~2900 m), *G. hazarensis* sp. nov. is found in temperate forests, which are cooler and moister and include a mix of coniferous (*Pinus wallichiana*, *Cedrus deodara*, *Abies pindrow*) and broad-leaved trees (*Quercus incana*, *Aesculus hippocastanum*). These forests offer greater structural complexity, higher humidity, and abundant prey, making them ecologically ideal for *G. hazarensis* sp. nov. Telford (1980) described the species (classified as *Agkistrodon himalayanus*) from the Kaghan Valley as common on wooded slopes (a large flat granite slab), observed basking in the morning sun. Some encountered specimens seem to be sluggish and docile when disturbed, others try to escape under rocks or into surrounding undergrowth. Showler (1998) reports on specimens referable to the Hazara Pitviper from the Palas Valley and describes sites between 2400 and 2750 m elevation where he found the pitvipers mostly on southern slopes. The slopes consisted mostly of scree and were covered with herbaceous vegetation, grass banks of *Ephedra gerardiana* and *Viburnum cottonifolium* shrubs. One specimen he observed near Satoe was in woodland dominated by *Picea smithiana* with *Cedrus deodara*, *Pinus wallichiana* and *Acer* sp. with sparse ground flora consisting of *Viola* sp., *Galium* sp., *Fragaria nubicola*, *Polygonum* sp. and ferns. Little is known about the food spectrum of the Hazara Pitviper. Only recently, Hadi and Junaid (2024) reported how they observed this pitviper (classified as *G. himalayanus*) on a Himalayan white pine (*Pinus wallichiana*) capturing a small passerine bird, the Rufous-bellied Niltava (*Niltava sundara*), and devouring it within four minutes. Reproductive data are only reported by Telford (1980) for three gravid females which gave birth to five to six neonates in captivity between the end of August and the beginning of September. At birth, the neonates had an average snout-vent length of 146.9 mm and an average tail length of 24.3 mm. Further research focusing on populations now assigned to *G. hazarensis* sp. nov. is necessary to better understand its ecological requirements across the species range.

##### Conservation.

The species is currently unprotected. Existing legal protection applies only to populations previously classified under *G. himalayanus*. The species is frequently killed by residents (Fig. 20D).

#### Gloydius nepalensis sp. nov.

Taxon classification

Animalia

SquamataViperidae

5E547ECE-BB9E-5272-8C7B-311678FF74DE

https://zoobank.org/1F673071-866E-4ECA-A46D-ED6546BCA7AE

##### Common names.

Nepali Pitviper (English), *Andho sarpa* and *Bhyagute sarpa* (Nepali, according to Shah and Tiwari 2004; Sharma et al. 2013).

##### Type material.

**Figure 21.**
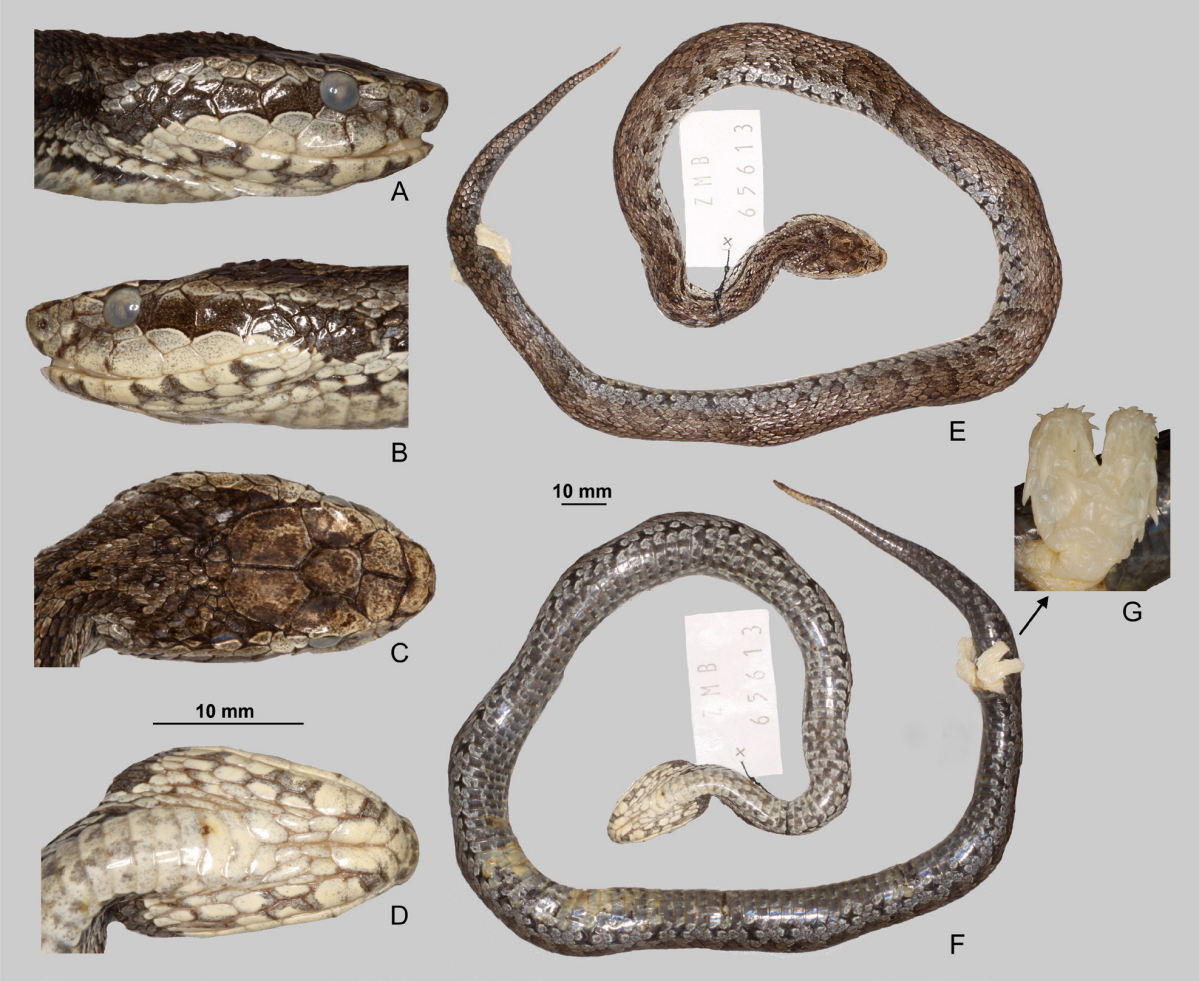
The adult male holotype of *Gloydius nepalensis* sp. nov. (ZMB 65613). **A–D**. Lateral, dorsal and ventral views of the head; (**E, F**) dorsal and ventral views of the body; **G**. Hemipenis.

***Holotype***. • ZMB 65613, an adult male from Kalopani village, Mustang District, Gandaki Province, Nepal, 28.6167°N, 83.6000°E, 2500 m altitude, donated by Matthias Lorenz (Fig. 21). ***Paratypes***. • BMNH 1953.1.1.71, juvenile female collected 8 miles west of Tibrikot, Dolpa District, Karnali Province, Nepal by William Russell Sykes in 1952; MHNG 1329.6 and MHNG 1329.8, male and female from Dhorpatan, Baglung District, Gandaki Province, Nepal, collected by L. Naef in September 1963 and R. Weiersmüller in October 1963 respectively; NME R 0544/07 (field no. SN 6500), adult male from Kermi, 14 km northwest of Simikot, Humla District, Karnali Province, Nepal, collected on 12 June 2001 by Jörg Weipert; NHMK 254 (formerly RMNH.RENA 20514), adult male from Gurja Ghat, 5 km east of Dhorpatan, Baglung District, Gandaki Province, Nepal, collected on 14 July 1981 by Paul E. Ouboter, Lurly M.R. Nanhoe and Karan B. Shah; RMNH.RENA.20512, adult male from Tal village, Manang District, Gandaki Province, Nepal, collected on 3 May 1981 by Lurly M.R. Nanhoe and Paul E. Ouboter; RMNH.RENA.20513, adult female from Dhorpatan, Baglung District, Gandaki Province, Nepal, collected on 13 July 1981 by Lurly M.R. Nanhoe and Paul E. Ouboter; ZMB 65612, adult female from Syang village (Jomsom) Mustang District, Gandaki Province, Nepal, donated by Matthias Lorenz; ZSM 154/1973/1–2, adult male and female, ZSM 154/1973/4, adult male, and ZSM 156/1973/1, adult male, all from Kalopani village, Mustang District, Gandaki Province, Nepal, collected on 8 June 1973 by Ulrich Gruber and Dieter Fuchs; ZSM 157/1973, adult male from Tukuche village, Mustang District, Gandaki Province, Nepal, collected on 23 June 1973 by Ulrich Gruber and Dieter Fuchs.

##### Description of the holotype.

Adult male, indicated by the presence of hemipenes; 1/1 loreal, significantly higher than wide; nasal completely divided; 2/2 elongated preoculars, the lower narrower; 1/1 supraocular; 2/2 postoculars, upper reaching onto top of head and touching the parietal, the lower can be described as postsubocular, because it extends under the eye, up to the level of the posterior border of the third supralabial; rostral scale wider than high; two internasals wider than long; two prefrontals, longer than wide; frontal bell-shaped, barely longer than wide; 2/2 anterior temporals, upper significantly smaller than the lower one, and 2/2 posterior temporals, the lower one slightly larger than upper and in contact with the second last supralabial; supralabials 7/7, second last nearly three times higher than the previous one, in contact with lower posterior temporal, 3^rd^/3^rd^ in contact with the eye; pit opening shorter than the horizontal diameter of eye and encircled by 3/3 scales; 14 circum-pileus scales; 9/9 sublabials, first three in contact with anterior submaxillars; two pairs of submaxillars, anterior twice as wide and 67% longer than posterior; followed by three rows of paired gular scales, increasing in size posteriorly; dorsal scales in 21-21-17 rows, strongly keeled, except the outer row, which is only very slightly keeled, paired apical pits on some dorsals, very weakly developed; three preventrals; 145 ventrals; cloacal plate entire; 42/42 paired subcaudal scales. Body compact, subcylindrical; tail short (TaL/TL 0.154); SVL 418 mm; TaL 76 mm, head length measured from tip of snout to posterior border of parietals 15.0 mm, head length measured from tip of snout to posterior edge of mandible 22.8 mm, head width 13.0 mm.

##### Dorsal scale reduction formula.

**Table:** 

-6(6)	-5(8)	3+4(14)	5+6(94)	4+5(116)
(5)**27** ---------------------- **25** -------------------------- **23** ------------------------- 2**1** ---------------------------- **19** --------------------------- **17**(145).				
-6(6)	-5(8)	4+5(14)	5+6(96)	4+5(116)

##### Dentition.

**Figure 22.**
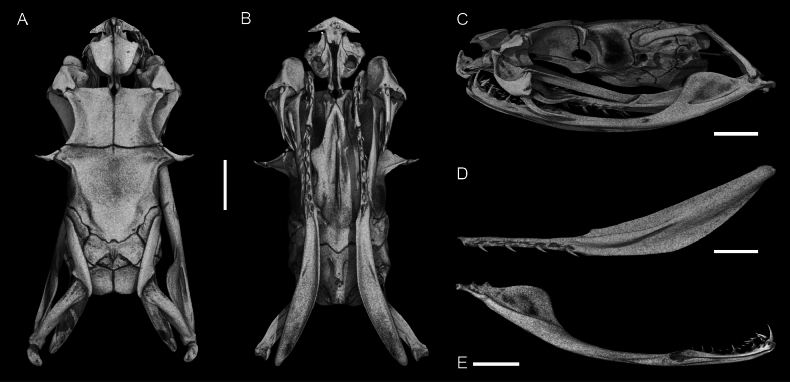
Skull of the adult male holotype of *Gloydius nepalensis* sp. nov., ZMB 65613: **A**. Dorsal, **B**. Ventral (lower jaws virtually extracted) and **C**. Lateral view; **D**. Pterygoid virtually isolated, ventral view; **E**. Lower jaw virtually isolated, medial view. Scale bar: 3.0 mm.

Maxillary bone with two posteriorly curved fangs on each side. On both sides the lateral fang is missing. Behind the main fangs are 7/8 replacement fangs at different growth stages. Main fang 3.98 mm in length, i.e., 22.8% of skull length. Discharge orifice 0.70 mm in length, i.e., 17.6% of fang length. Palatine bone with 3/3 posteriorly curved teeth slightly decreasing in size posteriorly. Teeth II loose on left, teeth I and III loose on right side. Lateral to each palatine tooth is a single replacement tooth at different growth stages. Pterygoid bone with 8/8 posteriorly curved teeth, shorter than the palatine tooth, all nearly the same size. Teeth I, III, V and VII loose on left side, as well as on right side. The posterior 64.5% of the pterygoid bone is without teeth. Mandibular bone with 11/12 posteriorly curved teeth gradually decreasing in size posteriorly. The first two teeth closer together than the rest. Medial to each mandibular tooth are up to two replacement teeth in different growth stages. Teeth I, III, V and VII loose on left side. Teeth I, III, VII, IX and XI loose on right side. Splenial and angular bones are fused. The total length of splenial-angular complex spans 30.3% of the mandibular bone. The dental is 37.6% as long as the mandibular bone. The complete skull of the holotype ZMB 65613 is presented in Fig. 22.

##### Colouration and pattern.

**Figure 23.**
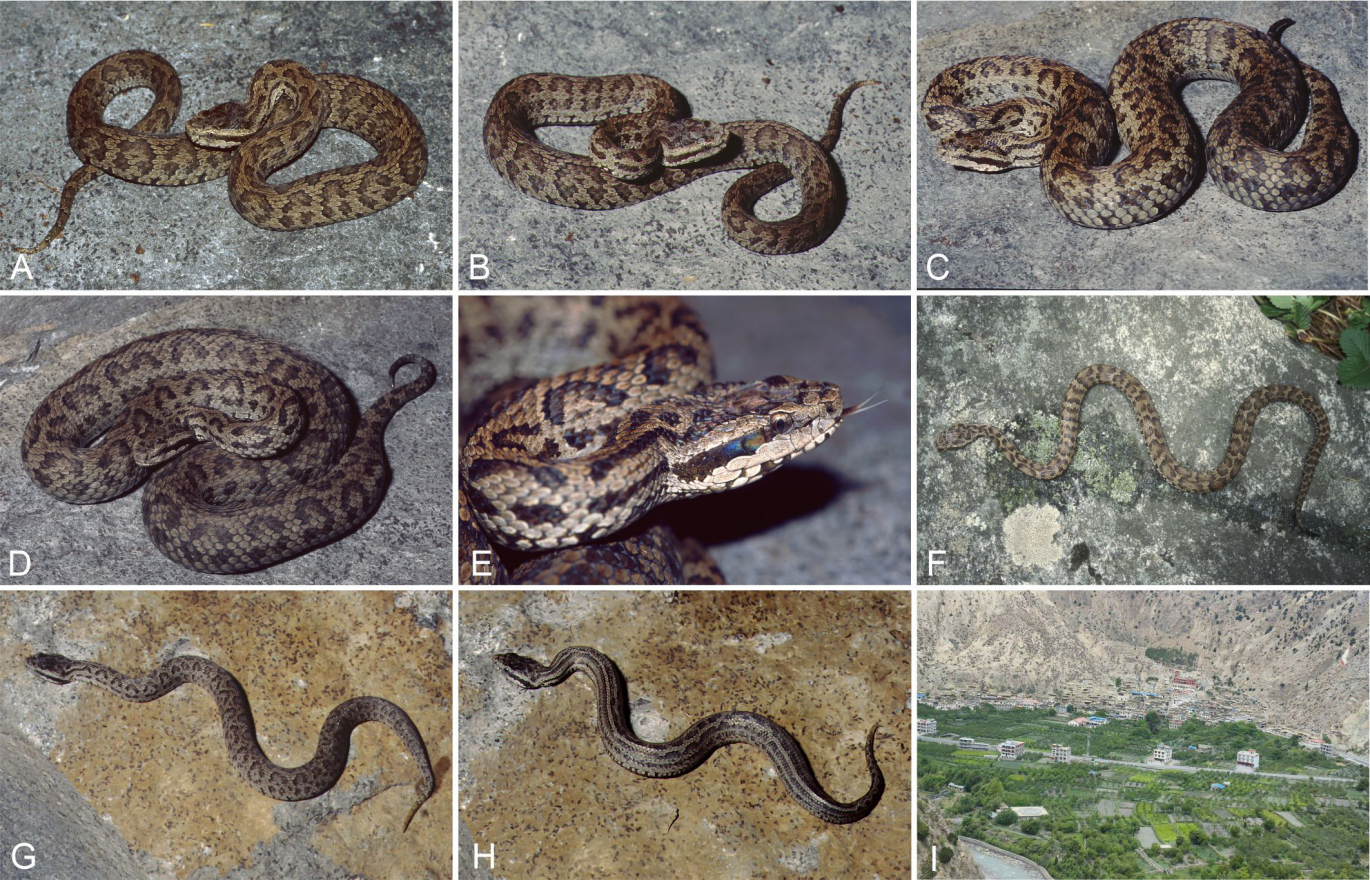
The colour and pattern in *Gloydius nepalensis* sp. nov. and its habitat. (**A, B**) The holotype (ZMB 65613, adult male) from Kalopani village, Mustang District, Gandaki Province, Nepal, 2500 m elevation; **C–E**. The paratype (ZMB 65612, adult female) from Syang village, Mustang District, Gandaki Province, Nepal, 2700 m elevation; **F**. The paratype (NME R 0544/07), adult male from Kermi, 14 km northwest of Simikot, Humla District, Karnali Province, Nepal (photo by Ulrich Scheidt); **G**. Neonate of the blotched phase (ZMB 65607, female, mother from Syang village, Mustang District, Gandaki Province, Nepal, 2700 m elevation); **H**. Neonate of the striped phase (ZMB 65606, male; mother from Syang village, Mustang District, Gandaki Province, Nepal, 2700 m elevation); **I**. Representative habitat of *G. nepalensis* sp. nov. in the vicinity of Marpha, Mustang District, Gandaki Province, Nepal, courtesy National Trust for Nature Conservation Annapurna Conservation Area Project, Jomsom, Mustang, Nepal.

Colouration after ~28 years preservation in ~70% ethanol was recorded as follows: Dorsal ground colour Medium Fawn (Colour 257), with 32 Olive-Brown (278) roundish dorsolateral blotches with lighter Medium Fawn (257) centre, the outer edges of the blotches with darker Sepia (279) coloured margins, some blotches with a small dark Olive-Brown (278) spot in their centres; other blotches can be divided horizontally and form two semicircles or break up into four curved spots; lateral body blotches do not merge vertebrally; all dorsal scales show a dense Dark Grayish Brown (284) mottling; below the lateral blotches, a row of small, irregular rhombic or x-shaped Sepia (279) coloured and Cream White (52) bordered spots runs along the outer edge of the ventrals; tail with nine irregularly shaped Olive-Brown (278) spots; dorsal and upper lateral head sides with same ground colour as dorsal body and densely mottled with Dark Grayish Brown (284); pileus with small irregular shaped Olive-Brown (278) spots; neck with three Olive-Brown (278) stripes, the middle one shortest; a wide Sepia (279) postocular stripe runs from the posterior border of the eye to the posterior edge of the mandible, whose lower edge is bordered by a Dark Grayish Brown (284) wavy, narrow line; anterior and lower parts of posterior supralabials Pale Buff (1) and mottled with Dark Grayish Brown (284); venter with a colour gradient that becomes darker towards the tail; throat Pale Buff (1) coloured, submaxillars and gulars with numerous small Dark Grayish Brown (284) spots; sublabials Pale Buff (1) with small irregular Dark Grayish Brown (284) spots on their sutures; posteriorly, the venter gradually changes, from Pale Neutral Gray (296) with heavy Dark Grayish Brown (284) mottling via Light Neutral Gray (297) to Medium Neutral Gray (298); additionally to the dark mottling all ventrals with three to five squarish Dark Neutral Gray (299) or Dark Grayish Brown (284) spots which merge in the posterior third of the body; tail tip Light Buff (2) coloured. Examples of variation in the colouration and pattern of live individuals; see Fig. 23.

**Table 5.** Summary of main dorsal scale reduction in % of ventrals for examined specimens of the *Gloydius himalayanus* complex (*individual data for both sexes not available).

Main DSR reductions	23-21			21-19			19-17		
	♂♂	♀♀	x̄	♂♂	♀♀	x̄	♂♂	♀♀	x̄
*G. himalayanus*	3.9–15.0%	6.6–16.0%	~9.2%	59.0–71.3%	55.1–72.7%	~66.3%	71.4–82.0%	64.0–72.5%	~72.3%
G. chambensis*	4.9–21.3%		~11.2%	41.1–68.8%		~55.8%	66.7–75.2%		~70.6%
*G. hazarensis* sp. nov.	7.2–9.2%	3.7–9.7%	~7.1%	33.6–45.4%	49.1–67.3%	~49.5%	73.0–75.7%	63.2–77.0%	~71.6%
*G. hindukushensis* sp. nov.	–	7.1–8.4%	~7.7%	–	53.9–65.2%	~59.5%	–	69.5–78.1%	~73.3%
*G. nepalensis* sp. nov.	5.5–15.2%	7.4–24.2%	~10.3%	56.2–68.8%	46.6–74.2%	~62.6%	64.9–80.0%	62.8–81.1%	~71.4%

**Table 6.** Main meristic and mensural traits of type material of *Gloydius hindukushensis* sp. nov.

	NHMW 41993	CUHC 10088
Type status	Holotype	Paratype
Sex	Female	Female
SVL	578	162
TaL	80	25
HL1	17.9	9.4
HL2	27.0	12.5
HW	15.9	8.3
DSR	23/21/19	23/21/17
PreVen	4	3
Ven	155	154
SubC	39	38
CpP	18	17
PrO	3/3	3/3
PoO	3/3	3/3
SupL	7/7	7/7
SubL	9/9	9/9

**Table 7.** Main meristic and mesural traits of type material of *Gloydius hazarensis* sp. nov.

	UF 70652	UF 70656	UF 70657	UF 82634	NHMW 17078:1
Type status	Holotype	Paratype	Paratype	Paratype	Paratype
Sex	Female	Female	Female	Female	Male
SVL	400	404	481	561	518
TaL	56	64	68	83	85
HL1	13.7	13.4	14.6	17.7	17.4
HL2	20.3	20.5	23.8	28.6	24.9
HW	11.3	10.9	13.2	15.9	17.2
DSR	21/20/17	21/20/17	21/21/17	21/21/17	23/21/17
PreVen	3	3	4	2	4
Ven	163	161	164	166	155
SubC	41	44	40	44	48
CpP	15	17	14	19	15
PrO	3/3	3/3	3/3	3/3	2/2
PoO	2/2	2/2	2/2	3/2	3/3
SupL	7/7	7/7	7/7	7/7	7/7
SubL	10/10	10/10	10/10	10/10	10/10

**Table 8.** Main meristic and mensural traits of type material of *Gloydius nepalensis* sp. nov.

	ZMB 65613	BMNH 1953.1.1.71	MHNG 1329.6	MHNG 1329.8	NHMK 254	NME R 0544/07	RMNH.RENA. 20512	RMNH.RENA. 20513	ZMB 65612	ZSM 154/1973/1	ZSM 154/1973/2	ZSM 154/1973/4	ZSM 156/1973/1	ZSM 157/1973
Type status	Holotype	Paratype	Paratype	Paratype	Paratype	Paratype	Paratype	Paratype	Paratype	Paratype	Paratype	Paratype	Paratype	Paratype
Sex	Male	Female	Male	Female	Male	Male	Male	Female	Female	Male	Female	Male	Male	Male
SVL	418	167	507	490	505	549	356	477	521	455	453	487	509	482
TaL	76	28	81	76	74	78	53	76	80	84	70	76	83	85
HL1	15.0	10.2	18.2	15.8	15.8	17.5	12.5	16.8	18.0	15.7	14.2	17.8	17.8	16.4
HL2	22.8	14.3	24.5	24.9	20.2	25.9	19.1	25.0	25.9	24.1	23.3	27.3	25.9	25.3
HW	13.0	7.9	13.2	14.3	14.7	17.4	12.1	15.1	17.0	16.3	15.3	20.0	18.8	14.6
DSR	21/ 21/17	23/ 21/17	21/ 19/15	21/ 19/17	n.n./19/n.n.	21/ 21/17	23/ 21/17	23/ 21/17	22/ 23/17	23/ 21/17	21/ 21/17	23/ 21/17	21/ 21/17	23/ 21/17
PreVen	3	3	1	2	1	1	2	2	3	2	1	3	2	3
Ven	145	159	150	149	145	158	145	150	153	147	154	146	147	145
SubC	42	41	41	39	41	44	43	39	41	43	40	39	39	42
CpP	14	16	16	16	13	17	14	13	18	15	14	15	14	15
PrO	2/2	2/2	2/2	2/2	2/2	2/2	2/2	2/2	2/2	2/2	2/2	2/2	2/2	2/2
PoO	2/2	2/2	2/2	2/2	2/2	3/2	2/2	3/3	2/2	2/2	2/2	2/2	2/3	2/2
SupL	7/7	7/7	6/6	6/7	7/7	7/7	7/7	7/7	8/7	7/7	7/7	7/7	7/7	7/7
SubL	9/9	9/10	9/10	7/7	9/9	9/9	9/9	8/8	9/8	9/9	9/9	9/9	8/8	9/9

##### Variation.

The paratypes and other examined material agree well with the holotype in general appearance. For differences based on sexual dimorphism, morphometrics and scalation we refer to Tables 3, 4, 8. Meristic and morphometric characters of *G. nepalensis* sp. nov. can be summarised as follows: a single loreal, higher than wide or sometimes as high as wide; nasal completely divided or only exceptionally partly divided below naris; always two elongated preoculars, the lower narrower; a single supraocular; usually two, rarely three postoculars, upper reaching onto top of head and touching the parietal, the lower one can be described as postsubocular and touches with its anterior side the upper posterior edge of the third supralabial; rostral scale wider than high; two internasals wider than long; two prefrontals, ratio of length to width variable sometimes almost the same length; frontal bell-shaped, little longer than wide or as long as wide; predominantly three, often two, exceptionally four anterior and predominantly three, rarely four or two, exceptionally five posterior temporals; usually seven, rarely six, exceptionally eight supralabials, always the third in contact with the eye; 13–20 circum-pileus scales; usually nine, sometimes eight, rarely seven or ten sublabials, predominantly first three, rarely first four in contact with anterior submaxillars; two pairs of submaxillars, anterior usually twice as wide and 31–100% longer than posterior; followed by 2–4 rows of paired gular scales, increasing in size posteriorly; anterior dorsal scales in 21 or 23, sometimes in 22 rows, midbody dorsal scales predominantly in 21, rarely in 19, exceptionally in 22 or 23 rows, posterior dorsal scales predominantly in 17, rarely in 15, exceptionally in 16 rows; predominantly two, sometimes one or three, rarely four or no preventrals; 140–161 ventrals (males 140–158, females 146–161); 38–48 paired subcaudal scales (males 39–48, females 38–42); sum of ventral and subcaudal scales 185–207 (males 185–207, females 188–205). Body compact, subcylindrical; tail short, ratio TaL/TL 0.124–0.172 (males 0.124–0.172, females 0.127–0.156); maximum recorded SVL of examined material in males 609 mm, in females 535 mm; maximum recorded TaL of examined material in males 93 mm, in females 80 mm; maximum recorded TL of examined material in males 648 mm, in females 614 mm.

##### Sum formula of dorsal scale reduction in males.

**Table:** 

(6–7)	(6–8)	(8–23)	(15–106)	(96–116)	(140)
-6, 5+6, -12	-5, 3+4, 4+5, 5+6	3+, 4+5, 5+6	-5, 4+5, 5+6	3+4, 4+5	-4
**(5)27 ---------------- 25 ---------------------- 23 ---------------- 21 -------------- 19 ------------- 17 ------------15**.					
-6, 3+4. -13	-5, 3+4, 4+5. 5+6	4+5, -10	4+5, 5+6, 6+7	3+4, 4+5	-4
(6)	(6–9)	(8–23)	(15–105)	(98–116)	(140)

##### Sum formula of dorsal scale reduction in females.

**Table:** 

(7)	(6–16)	(11–92)	(69–115)	(93–133)
-6	3+4, 5+6	4+5, 5+6, -V	-5, 4+5, 5+6, -10	3+4, 4+5
**(5)27 ----------25 -------------------- 23 ------------------- 21 ----------------------- 19 ------------------------- 17**.				
-6	4+5, 5+6	4+5, 5+6	4+5, -5, 5+6	4+5
(7)	(7–17)	(11–18)	(74–112)	(96–131)

##### Variation in dentition.

Maxillary bone with two posteriorly curved fangs on each side. Main fang 3.38–5.85 mm in length, i.e., 22.8–30.6% of skull length. Discharge orifice 0.55–1.35 mm in length, i.e., 14.84–23.1% of fang length. Palatine bone with three or four posteriorly curved teeth slightly decreasing in size posteriorly. Pterygoid bone with 7–10 posteriorly curved teeth, shorter than the palatine tooth, all nearly the same size. The posterior 60.9–66.1% of the pterygoid bone is without teeth. Mandibular bone with 10–12 posteriorly curved teeth gradually decreasing in size posteriorly. The first two teeth closer together than the rest. Splenial either 87.7–173.9% of length of angular or fused. The total length of splenial-angular complex spans 24.2–36.6% of the mandibular bone. The dental is 35.4–38.8% as long as the mandibular bone.

##### Variation in live colouration and pattern.

Dorsal ground colour can vary from Tawny Olive (Colour 17) to Drab-Gray (256) or Smoke Gray (266); number of dorsolateral blotches varies from 26–33 on body and from 6–10 on tail; in some populations (e.g., Humla) body blotches can merge vertebrally and form irregularly shaped broad bands especially across the posterior part of body and tail; rarely a nearly completely striped morph is reported (Gumprecht et al. 2004: 66, fig. 1; Sharma et al. 2013: 51, fig. 83); dorsal ground colour can darken considerably to Sepia (279) during preservation, so that the dorsal pattern elements of the head and body are no longer recognisable.

##### Etymology.

The specific epithet *nepalensis* is a latinised adjective meaning "from Nepal," referring to the country where the species was discovered. Located in the central Himalaya, Nepal encompasses an exceptionally diverse range of ecosystems, from subtropical lowlands to alpine environments. The name honours the geographic origin of the species and highlights the country’s ecological significance as part of the Himalayan biodiversity hotspot, which supports many range-restricted and endemic species. It also acknowledges Nepal’s increasing role in herpetological research and conservation in South Asia. The name has previously been used for various amphibian and reptile species, including *Scutiger nepalensis* Dubois, 1974, *Minervarya nepalensis* (Dubois, 1975), *Cyrtodactylus nepalensis* (Schleich & Kästle, 1998), or *Ablepharus nepalensis* (Eremchenko & Helfenberger, 1998).

##### Distribution.

*Gloydius nepalensis* sp. nov. is a central Himalayan species and so far reported only from western and west-central Nepal (Fig. 9). Records are known from the districts of Humla, Jumla, and Dolpa in the Karnali Province and from the districts of Mustang, Baglung, Myagdi, and Manang in the Gandaki Province (own data; Smith and Battersby 1953; Kramer 1977; Nanhoe and Ouboter 1987; Sura 1987; Gloyd and Conant 1990; Shah 1995; Schleich and Kästle 2002; see also Fig. 9 and Suppl. material 1: table SS3). The distribution of the species is based on 26 geographic coordinates forming a polygon with a range of ca 20,400 km^2^.

Various authors (e.g., Rai 2002; Schleich and Kästle 2002; Shah and Tiwari 2004; Kästle et al. 2013) list *G. himalayanus* (sensu lato) for eastern Nepal. According to our research, there is still no credible evidence, neither photographs nor preserved voucher specimens, from east of the Annapurna region. Earlier records were mostly based on misidentified Mountain Pitvipers (*Ovophis monticola*) as listed and depicted in Schleich and Kästle (2002: 1068 No 1, 2 and 15, pl. 111, figs 329–330; see also comments by Gumprecht et al. 2004: 20).

##### Habitat and ecology.

*Gloydius nepalensis* sp. nov. is recorded from elevations between 1640 and 3220 m (see Suppl. material 1: table SS3). The species is associated with dry areas where it was found in open rocky habitats with scattered small bushes and stones on slopes near dry coniferous forests consisting of *Picea* and *Pinus* spp. It is recorded from subalpine shrublands and alpine meadows but also from the vicinity of small rivers with low trees and near cultivated areas (Dierl and Gruber 1979; Nanhoe and Ouboter 1987: 47; Gumprecht et al. 2004: 348 fig. I; Shah and Tiwari 2004). This species is not strictly nocturnal. It feeds on frogs, small lizards, and rodents (pers. obs.; Nanhoe and Ouboter 1987; Schleich and Kästle 2002; Shah and Tiwari 2004). Hibernation periods differ depending on local climate and altitude and can last up to seven months. Reproduction is ovoviviparous with mating in April or May and the birth of 5–7 neonates in September, with a total length varying from 163–199 mm and a weight of 3.5–5.1 g (pers. obs.).

##### Conservation.

The species is considered to be common in the distributional area (Shah 1995; Shrestha 2001; Schleich and Kästle 2002; Shah and Tiwari 2004) and Nepalese populations are listed under *Gloydius himalayanus* as "Least Concern" by the IUCN Red List of Threatened Species (Limbu and Ghosh 2022).

## Discussion

Our study presents the most comprehensive taxonomic assessment to date of the *G. himalayanus* complex, using an extensive integrative approach, and reveals substantially greater diversity in the Himalaya and Hindu Kush than previously recognised. Until now, only two species within this complex, the type species *G. himalayanus* (first described by Albert Carl Ludwig Gotthilf Günther in 1864 as *Halys himalayanus*) and *G. chambensis* had been genetically examined (Shi et al. 2021; Kuttalam et al. 2022). However, our integrated genetic, morphological, and osteological analyses, together with ecological and distributional evidence, identify three additional well-supported evolutionary lineages in the region. The discovery of five species within the same complex not only substantially increases the known diversity but also reveals phenotypic variation related to discovered lineages that had previously been misinterpreted as intraspecific polymorphism (Gloyd and Conant 1990). The description of *G. hazarensis* sp. nov., *G. hindukushensis* sp. nov., and *G. nepalensis* sp. nov., more than 160 years after the initial description of *G. himalayanus*, thus underscores the long-term underestimation of cryptic diversity in this region and highlights the taxonomic neglect of the Himalaya and adjacent Hindu Kush.

In mountain systems, deeply incised river valleys often coincide with sharp faunal turnover and can function as long-term barriers to dispersal, thereby promoting lineage sorting and allopatric divergence (e.g., Zhou et al. 2017; Dufresnes et al. 2026). In this context, we provisionally suggest that the distributions of the lineages within the *G. himalayanus* complex are structured by four major mountain river valleys (Fig. 9): the Upper Indus Valley (between the Hindu Kush and Himalaya), the Kashmir Valley, the Ravi Valley (western Himalaya), and the Karnali Valley (central Himalaya). These valleys may have functioned as historical barriers, promoting allopatric diversification and shaping the present-day distribution of the complex. Compared with previous studies related to the genus *Gloydius*, our results refine both the diversity and geographic limits of the complex, notably by clarifying the distribution of the nominal species *G. himalayanus* (now restricted to the western Himalaya, i.e., Himachal Pradesh and Uttarakhand, India; Fig. 9) and by demonstrating the westernmost extension of *G. chambensis* into the Kashmir Valley based on museogenomic data. Future work should further resolve these distribution boundaries, particularly across the putative contact zones (Fig. 9) that remain poorly constrained, most notably between *G. chambensis* and *G. himalayanus*, given currently available sampling and data. Overall, our findings underscore the value of integrative, multi-evidence approaches for resolving long-standing taxonomic uncertainties and for delineating species’ ranges in the still under-studied high-elevation faunas of High Asia.

Although the five lineages identified here exhibit clear genetic differentiation, divergence levels across *Gloydius* are highly variable (cf. Ding et al. 2011; Asadi et al. 2019; Shi et al. 2021; Kuttalam et al. 2022; Zhang et al. 2022; Xu et al. 2023). This variability cautions against relying primarily on mitochondrial data for species delimitation, as such an approach may contribute to taxonomic inflation (Dufresnes et al. 2023; Wüster et al. 2024), a concern already raised for several *Gloydius* species descriptions (e.g., Wagner et al. 2016b). Without consistently available nuclear DNA datasets, diversity within *Gloydius* cannot be robustly assessed; therefore, further descriptions, particularly of shallow lineages within the genus, should be treated with caution. While our genetic evidence is dominated by mitochondrial markers, we also include phased nuclear data, and our inference is not based on DNA alone. Instead, we apply an integrative framework incorporating extensive evidence from external morphology, osteology, ecology, and distribution, which independently supports the recognition of these lineages as deeply divergent and distinct. Moreover, the genetic distances among lineages within the *G. himalayanus* complex are substantially greater than those reported for several other *Gloydius* taxa described using more limited datasets (e.g., cyt *b* divergence of ~2.2% between *G. intermedius* and *G. shedaoensis*, and similarly low values among other species, versus >10% on the same marker among lineages within the *G. himalayanus* complex; Suppl. material 1: table SS2).

In this context, questions arise regarding the potential need for future taxonomic re-evaluation of some *Gloydius* species characterised by low genetic divergence, which may instead represent intraspecific variation (subspecies rank) rather than distinct species. Therefore, there is a clear need to establish criteria that define the taxonomic level appropriate for a given phylogenetic lineage within the genus *Gloydius*. Genomic-scale approaches, which can reveal mito-nuclear inconsistencies, are expected to play a crucial role in developing these criteria and refining species boundaries in future taxonomic studies. Even within the *G. himalayanus* complex itself, further taxonomic changes cannot be ruled out. The intraspecific genetic variability observed in *G. himalayanus* or *G. hazarensis* sp. nov. is noteworthy (Fig. 2A; PMNH 4110 vs 4240). These findings underscore the importance of future comprehensive genomic-scale analyses (if suitable samples become available) which will be essential for clarifying taxonomic limits.

In the regional context, a particularly important taxonomic question arises regarding *Gloydius halys boehmei* Nilson, 1983, described from the northwestern slopes of the Hindu Kush region of Afghanistan. Apart from belonging to the *G. halys* complex and having a distinguishable colour pattern, *G. h. boehmei* is morphologically differentiated from *G. hindukushensis* sp. nov. by several additional traits, including the presence of 23 scale rows at mid-dorsum (vs 21), a lower number of subcaudals (35–40 vs 38–50) and a higher number of sublabials (10–11 vs 7–9). However, the newly described *G. hindukushensis* sp. nov. (although not yet confirmed for Afghanistan) occurs geographically close to the type locality of *G. h. boehmei* (Nilson, 1983) (ca 290 km in a straight line). The geographic distribution of genealogical lineages suggests a major break associated with the Hindu Kush, separating main clades of the genus, namely the *halys* and *himalayanus* complexes. Fieldwork and DNA sampling in eastern and central Afghanistan, particularly in Baghlan, Kabul, Nangarhar, Nuristan, Panjsheer, and adjacent provinces, are critical to resolving these taxonomic and phylogeographic questions.

In conclusion, our results represent a substantial step forward in understanding the biodiversity of the southern part of High Asia and help resolve the long-standing taxonomic uncertainties within the *G. himalayanus* complex. They further emphasise the broader need for integrative taxonomic and biogeographic studies of the region’s fauna. As observed in the *G. himalayanus* complex, several recent studies have revealed the presence of ancestral or deeply divergent phylogenetic lineages (often representing independent but morphologically similar taxa) distributed in the Himalaya and along the Hindu Kush, particularly among amphibians and reptiles (Agarwal et al. 2014, 2022; Gowande et al. 2021; Hofmann et al. 2017, 2019, 2024a, 2024b; Dufresnes et al. 2019; Jablonski et al. 2021b, 2024; Mirza et al. 2021; Lee et al. 2023; Bhattarai et al. 2025). These recent findings suggest that the Himalaya and Hindu Kush serve as an exceptional natural laboratory for studying speciation processes and for refining taxonomic frameworks within some of Earth’s most complex montane ecosystems.

## Supplementary Material

XML Treatment for Gloydius chambensis

XML Treatment for Gloydius himalayanus

XML Treatment for Gloydius hindukushensis

XML Treatment for Gloydius hazarensis

XML Treatment for Gloydius nepalensis

## Additional information

### Artificial Intelligence (AI) use

Regarding the use of AI in the preparation of this manuscript, the authors declare the following:

AI-assisted copy editing.

### Author ORCIDs

Daniel Jablonski https://orcid.org/0000-0002-5394-0114

Frank Tillack https://orcid.org/0000-0003-1418-6531

Kristin Mahlow-Tillack https://orcid.org/0000-0002-0625-965X

Alice Petzold https://orcid.org/0000-0002-0743-2004

Chitra B. Baniya https://orcid.org/0000-0002-8746-7601

Rafaqat Masroor https://orcid.org/0000-0001-6248-546X

Sylvia Hofmann https://orcid.org/0000-0003-0621-9049

### Data availability

All of the data that support the findings of this study are available in the main text or Supplementary Information.

## Supplementary materials

Supplementary material 1 Additional information Data type pdf https://binary.pensoft.net/file/1652575This dataset is made available under the Open Database License (http://opendatacommons.org/licenses/odbl/1.0/). The Open Database License (ODbL) is a license agreement intended to allow users to freely share, modify, and use this Dataset while maintaining this same freedom for others, provided that the original source and author(s) are credited. Daniel Jablonski, Frank Tillack, Kristin Mahlow-Tillack, Alice Petzold, Madita Wilzo, Abhijit Das, Muhammad Idrees, Chitra B. Baniya, Rafaqat Masroor, Sylvia Hofmann
