# Supplementary material for: Integrative taxonomy reveals previously undescribed diversity within the Gloydius himalayanus complex (Squamata, Viperidae, Crotalinae) from the Himalaya and Hindu Kush
Source: Zookeys. 2026 May 21;1280:83–153. doi: 10.3897/zookeys.1280.182768 (PMC13220052; doi:10.3897/zookeys.1280.182768)

A peer-reviewed open-access journal
ZooKeys
Launched to accelerate biodiversity research

Supplementary material for

Daniel Jablonski, Frank Tillack, Kristin Mahlow-Tillack, Alice Petzold, Madita Wilzo, Abhijit Das, Muhammad Idrees, Chitra B. Baniya, Rafaqat Masroor, Sylvia Hofmann

Integrative taxonomy reveals an undescribed diversity in the *Gloydius himalayanus* complex (Squamata, Viperidae, Crotalinae) from the Himalaya and Hindu Kush

Supplementary material 1

Table S1. Accession numbers and internal specimen identifiers used in genetic datasets for *Gloydius* species and outgroup taxa. The table includes markers from mitochondrial DNA (12S, 16S, cytochrome *b*, ND4) and nuclear DNA (PRLR, NT3, C-mos), with checkmarks indicating inclusion in each dataset. Specimen codes and GenBank accession numbers are provided where available.

species	dataset 1 (mtDNA 2,693 bp)	dataset 2 (mt+nDNA 3,882 bp)	Internal or voucher numbers	12S (408 bp)	16S (482 bp)	cytochrome *b* (1125 bp)	ND4 (678 bp)	PRLR (208 bp)	NT3 (473 bp)	C-mos (508 bp)
Gloydius angusticeps		√	JS1708G5C	-	-	-	-	OP450854	OP450889	OP422579
Gloydius angusticeps	√		JS1306G1A	KY040540	KY040572	KY040626	KY040647	-	-	-
Gloydius angusticeps		√	SH-2014 PLF5	-	-	KF997927	KF997980	-	-	KF997937
Gloydius blomhoffi	√	√	B524	AY352780	AY352719	AY352751	AY35281	OP450855	OP450890	OP422580
Gloydius brevicaudus	√		-	EU913477	EU913477	EU913477	EU913477	-	-	-
Gloydius caraganus	√		426	MZ958021	MZ957012	MZ959165	MZ959158	-	-	-
Gloydius caucasicus	√		913	MZ958022	MZ957013	MZ959166	MZ959159	-	-	-
Gloydius chambensis	√	√	17.v13	-	**	**	**	**	OP450900	OP422591
Gloydius chambensis	√	√	17.v20	OP508266	OP518268	OP480162	OP407965	OP450867	OP450901	OP422592
Gloydius chambensis	√	√	HARC-R 259 (18.13)	-	OP518269	-	OP407966	OP450868	-	OP422588
Gloydius chambensis		√	19.38	**	**	**	**	OP450871	OP450902	OP422593
Gloydius chambensis	√	√	19.40	-	OP518272	OP480164	-	OP450872	OP450903	OP422594
Gloydius chambensis	√	√	19.44	OP508268	OP518273	-	OP407970	OP450873	OP450904	OP422589
Gloydius chambensis	√	√	19.50	OP508269	-	OP480165	OP407971	OP450874	OP450905	OP422595
Gloydius chambensis	√	√	NHMW 17079.1	PX280590	PX280554	-	-	-	-	-
Gloydius chambensis	√	√	BMNH 1898.5.17.4	PX280591	PX280555	PX290130	PX290141	-	-	-
Gloydius chambensis	√	√	BMNH 1898.5.17.5	PX280592	PX280556	PX290131	PX290140	-	-	-
Gloydius changdaoensis		√	B893	OP508265	-	OP480160	-	OP450860	-	OP422583
Gloydius changdaoensis		√	B895	-	-	-	-	OP450861	OP450895	-
Gloydius changdaoensis		√	JSSD1510C1	-	-	KX063823	KX063796	OP450862	OP450896	OP422584
Gloydius changdaoensis	√		-	MT731652	MT731652	MT731652	MT731652	-	-	-
Gloydius cognatus (*intermedius* in GenBank)		√	GP188	-	-	JQ687488	JQ687469	-	-	JQ687507
Gloydius cognatus		√	L1	-	-	-	-	OP450863	OP450897	OP422585
Gloydius cognatus	√		JS130947	KY040532	KY040564	KY040622	KY040643	-	-	-
Gloydius halys		√	DLG11	-	-	KX063805	KX063778	OP450864	OP450898	OP422586
Gloydius halys		√	JS1407H9	-	-	KY040618	KY040639	OP450865	OP450899	OP422587
Gloydius halys	√		SYNU1301908	KY040526	KY040558	KX063802	KX063775	-	-	-
Gloydius hazarensis sp. nov.	√	√	PMNH 4110	PX280593	PX280557	PX290126	PX290138	-	PX290112	-
Gloydius hazarensis sp. nov.	√	√	PMNH 4240	PX280594	PX280558	PX290118	-	-	-	PX290114
Gloydius himalayanus	√	√	17.v03	OP508270	OP518274	OP480166	OP407972	OP450866	OP450906	-
Gloydius himalayanus		√	18.30	-	-	-	-	OP450869	-	OP422596

Gloydius himalayanus	√	√	18.31	-	-	-	OP407973	-	-	OP422597
Gloydius himalayanus	√	√	HM 19.30	MZ958982	MZ958980	MZ959173	MZ959172	OP450870	OP450907	OP422598
Gloydius himalayanus	√	√	MK559438	MK559438	MK559438	MK559438	MK559438	-	-	-
Gloydius himalayanus	√	√	BB87	-	-	PX290127	-	-	-	-
Gloydius himalayanus	√	√	BB88	-	-	PX290128	-	-	-	-
Gloydius himalayanus	√	√	BMNH 1946.1.19.64	PX280595	PX280559	-	-	-	-	-
Gloydius himalayanus	√	√	ZMB 2940	PX280596	PX280560	PX290129	PX290139	-	-	-
Gloydius hindukushensis sp. nov.	√	√	NHMW 41993	PX280598	PX280561	PX290119	PX290133	PX290110	-	-
Gloydius hindukushensis sp. nov.	√	√	CUHC 10088	PX280597	PX280562	PX290120	PX290134	-	PX290113	-
Gloydius hindukushensis sp. nov.	√	√	PMNH 5150	-	PX280563	PX290121	PX290135	PX290111	-	-
Gloydius huangi	√		CIB533422012	MW722961	MW721129	MZ355578	MW732035	-	-	-
Gloydius intermedius		√	GP1328		OP518276	JQ687502	JQ687483	OP450875	-	JQ687521
Gloydius intermedius	√		-	KM186844	KM186844	KM186844	KM186844	-	-	-
Gloydius lateralis	√		CIB119377 JZ01	ON362225	ON362229	ON42341	ON423421	-	-	-
Gloydius lipipengi	√		JS1408G2	KY040542	KY040574	KY040628	KY040649	-	-	-
Gloydius liupanensis	√	√	GP198	OP508271	OP518277	JQ687491	JQ687472	OP450876	-	JQ687510
Gloydius liupanensis		√	GP206	OP508272	-	JQ687492	JQ687473	OP450877	-	JQ687511
Gloydius monticola	√		JS1607DL1	KY040549	KY040581	KY040635	-	-	-	-
Gloydius nepalensis sp. nov.	√	√	NME 070555	PX280600	PX280564	PX290116	PX290132	-	-	-
Gloydius nepalensis sp. nov.	√	√	NME R 054407	PX280604	-	PX290117	-	-	-	-
Gloydius nepalensis sp. nov.	√	√	RMNH.RENA 20512	PX280599	PX280565	-	-	-	-	-
Gloydius nepalensis sp. nov.	√	√	ZMB 65611	PX280601	PX280566	PX290122	-	-	-	-
Gloydius nepalensis sp. nov.	√	√	ZMB 65612	PX280602	PX280567	PX290123	PX290136	PX290109	-	-
Gloydius nepalensis sp. nov.	√	√	ZMB 65613	PX280603	PX280568	PX290124	PX290137	-	-	-
Gloydius qinlingensis		√	GP197	OP508273	-	JQ687490	JQ687471	OP450879	OP450911	JQ687509
Gloydius qinlingensis	√	√	JS1505QL1	KY040534	KY040566	KY040623	KY040644	OP450880	OP450912	OP422600
Gloydius qinlingensis		√	QL2	-	-	-	-	OP450878	OP450910	OP422599
Gloydius qinlingensis (*rubromaculatus* in GenBank)	√		SAFS2019001	MW690593	MW690593	MW690593	MW690593	-	-	-
Gloydius qinlingensis (*strauchi* in GenBank)	√		-	MF523224	MF523224	MF523224	MF523224	-	-	-
Gloydius rickmersi	√	√	ZMB 80360	-	-	PX290125	-	-	-	PX290115
Gloydius rickmersi	√		MHNG 2752.69	-	-	-	KM078592	-	-	-
Gloydius rubromaculatus	√	√	IOZ002317	KY040546	KY040578	KY040632	KY040653	OP450881	OP450913	OP422601
Gloydius rubromaculatus		√	JS1607Y5	KY040548	KY040580	KY040634	KY040655	OP450882	OP450914	OP422602
Gloydius saxatilis		√	GP191	-	-	JQ687489	JQ687470	-	-	JQ687508
Gloydius shedaoensis		√	B849	-	OP518278	-	OP407974	OP450883	OP450915	OP422603
Gloydius shedaoensis		√	B856	OP508274	OP518279	OP480167	OP407975	OP450884	OP450916	OP422604
Gloydius shedaoensis		√	GP1100	-	-	JQ687498	JQ687479	-	-	JQ687517
Gloydius shedaoensis		√	GP1110	-	-	JQ687499	JQ687480	-	-	JQ687518
Gloydius shedaoensis	√		1119	KT726957	KT726957	KT726957	KT726957	-	-	-
Gloydius stejnegeri		√	JS151054	KY040539	KY040571	KY040625	KY040646	OP450886	OP450918	OP422605

Gloydius stejnegeri	√		SYNU1510145	KY040538	KY040570	KX063815	KX063788	-	-	-
Gloydius strauchi		√	JSI1410G3	-	-	-	-	OP450887	OP450919	OP422606
Gloydius strauchi	√		JS1309G3	KY040543	KY040575	KY040629	KY040650	-	-	-
Gloydius swild		√	GP175	-	-	JQ687487	JQ687468	-	-	JQ687506
Gloydius swild	√		GR1	OK210582	OK184551	OK239653	OK239648	-	-	-
Gloydius tsushimaensis	√		-	JN870186	JN870196	JN870203	JN870211	-	-	-
Gloydius ussuriensis	√		-	KP262412	KP262412	KP262412	KP262412	-	-	-
Gloydius ussuriensis		√	GP1326	OP508275	OP518280	JQ687501	JQ687482	OP450888	-	JQ687520
Gloydius variegatus	√		CD2	OK210581	OK184550	OK239652	OK239647	-	-	-
Crotalus adamanteus - outgroup	√	√	ROM 18130	MH626511	MH626511	MH626511	MH626511	-	KX694982	KX694780
Porthridium arcosae - outgroup	√		750	EU624241	EU624275	-	-	-	-	-
Porthridium lansbergii rozei - outgroup	√		787	EU624242	EU624276	AY713375	-	-	-	-
Porthridium nasutum - outgroup	√	√	ROM 42214	KX694567	KX694658	KX694838	-	-	KX694988	KX694790
Sistrurus miliarius - outgroup	√		-	MK330875	MK330875	MK330875	MK330875	-	-	-

**We treated specimens 17.v13, 19.38 and their sequences as likely misidentified or contaminated and excluded them from all tree analyses, because BLAST searches of the cytochrome *b* (OP480163) and PRLR (OP776641) sequences attributed to *Gloydius chambensis* by Kuttalam et al. (2022) recovered top matches to *Daboia russelii*.

The material of the genus *Gloydius* examined for this study

Symbol ● denotes for specimens CT scanned for analysis of the osteology and symbol * for specimens used in analysis of dorsal scale reduction.

Gloydius chambensis

India: BMNH 1898.5.17.4●*–5*, Pangi River Valley, Himachal Pradesh State; HARC R259 (holotype of *Gloydius chambensis* Kuttalam et al., 2022), Bhanjraru, Chamba District, Himachal Pradesh State; NMW 17079:1●*, Srinagar, Kashmir Division, Jammu and Kashmir State.

Gloydius halys caraganus

Kazakhstan: ZMB 8729●, “Mangyschlak” [Mangyshlak Peninsula], Mangystau Region; ZMB 66160–66164●, Syr Darya region.

Gloydius halys caucasicus

Afghanistan: NHMD R-6912●*, Sabzak Pass (Kowtal-e Sabzak), Paropamisu Range, Herat Province.

Gloydius halys boehmei

Afghanistan: ZFMK 8648●* (holotype of *Agkistrodon halys boehmei* Nilson, 1983), Andarab River Valley, Andarab District, Baghlan Province.

Gloydius halys halys

Russia: ZMB 84762●, "Tscholesmantal" [Chulyshman River Valley], Altai Republic.

Gloydius hazarensis sp. nov.

Pakistan: BNHS 2504, Thandiani, Abbottabad District, Khyber Pakhtunkhwa Province; BNHS 2516, Rawalpindi, Punjab Province; NMW 17078:1●* (paratype of *G. hazarensis* sp. nov.), Murree, Murree District, Punjab Province; PMNH 318, Kel, Neelum River Valley, Neelam District, Azad Jammu and Kashmir, Pakistan-administered region; PMNH 396, Par Palas, Kolai-Palas District, Hazara Division, Khyber Pakhtunkhwa; PMNH 1655, Makra Top, Kotla River Valley, Machiara National Park, Bagh district, Azad Jammu and Kashmir, Pakistan-administered region; PMNH 1979, Badi (Large), Gagai Nala, Upper Taobat village, Neelum River Valley, Sharda Tehsil, Neelam District, Azad Jammu and Kashmir, Pakistan-administered

region; PMNH 4109–4110, Sharan (Manchi Forest), Kaghan River Valley, Mansehra District, Khyber Pakhtunkhwa Province; PMNH 4240, Mughalabad, Murree Tehsil, Rawalpindi District, Punjab Province; UF 70652●* (holotype of *G. hazarensis* sp. nov.), UF 70665●, UF 70656* (paratype of *G. hazarensis* sp. nov.), UF 70657●* (paratype of *G. hazarensis* sp. nov.), UF 70658*, UF 70672, Naran town, Mansehra District, Khyber Pakhtunkhwa; UF 82634●* (paratype of *G. hazarensis* sp. nov.), Nathia Gali, Abbottabad District, Khyber Pakhtunkhwa.

Gloydius himalayanus

India: BMNH 1946.1.19.64●* (formerly BMNH 1860.3.19.1358), (lectotype of *Halys himalayanus* Günther, 1864 by present designation), „Garhval Himalayas", Uttarakhant State; BMNH 1946.1.18.75●* (formerly BMNH 1860.3.19.1189), (paralectotype of *Halys himalayanus* Günther, 1864 by present designation), „Garhval Himalayas", Uttarakhant State; BMNH 1872.4.17.352 "Khassya" [in error; Western Himalayas]; BMNH 1872.4.17.353 "Khassya" [in error; Western Himalayas]; BMNH 1872.4.17.354 "Khassya" [in error; Western Himalayas]; BNHS 2505, Kullu, Kullu District, Himachal Pradesh State; BNHS 2506, Shimla, Himachal Pradesh State; BNHS 2507, Dalhousie, Chamba District, Himachal Pradesh State; BNHS 2508, Dalhousie, Chamba District, Himachal Pradesh State; BNHS 2509, Shimla, Himachal Pradesh State; BNHS 2511, Manali, Kullu District, Himachal Pradesh State; BNHS 2513, Dalhousie, Chamba District, Himachal Pradesh State; BNHS 2514, Dalhousie, Chamba District, Himachal Pradesh State; NMW 15134:2* Kotgur [Kothgar] village, Thesil Kumarsain, Simla District, Himachal Pradesh State; NMW 17077:2* Shimla, Himachal Pradesh State; RMNH.RENA.4096A*, Shimla, Himachal Pradesh State; RMNH.RENA.4096B●*, Shimla, Himachal Pradesh State; RMNH.RENA.4096C●*, Shimla, Himachal Pradesh State; SMNS 4374●*, Dehradun, Uttarakhand State; WII-ADS81*, Sau Kharak, Chamoli District, Uttarakhand State; WII-ADR199, Benog, Dehradun district, Uttarakhand State; WII-ADR2023, Taluka, Uttarkashi District, Uttarakhand State; WII ADS82*; ZMA.RENA.16233*, Rampur Bushahr, Shimla District, Himachal Pradesh State; ZMB 8452, Darjeeling [in error]; ZMB 32485 without exact locality; ZMB 2940 Kotgur [Kothgar] village, Thesil Kumarsain, Simla District, Himachal Pradesh State; ZMB 65963 (formerly ZMB 2940 B), Kotgur [Kothgar] village, Thesil Kumarsain, Simla District, Himachal Pradesh State; ZMH R04852*–R04856*, R04860*, and R04880*, all from Molta, Chamoli District, Uttarakhand State; ZMH R04857*, Timli, Dehradun District, Uttarakhand State [in error, see discussion above under distribution].

Gloydius hindukushensis sp. nov.

Pakistan: BNHS 2517, Chitral, Chitral District, Khyber Pakhtunkhwa Province; CUHC 10088* (paratype of *G. hindukushensis* sp. nov.), Kumrat Valley, Upper Dir District, Khyber Pakhtunkhwa Province; NMW 41993●*, formerly CUHC 10085 (holotype of *G. hindukushensis* sp. nov.), Kumrat Valley, Upper Dir District, Khyber Pakhtunkhwa Province; PMNH 5150 (p21), Lal qilla, Lower Dir District, Khyber Pakhtunkhwa Province, N 34.98984 E 71.90301, 2700 m a.s.l.; PMNH 5149 (p48) and PMNH 5151, Lal qilla, Lower Dir District, Khyber Pakhtunkhwa Province, 35.03375, 71.86942, 2446 m a.s.l.

Gloydius nepalensis sp. nov.

Nepal: BMNH 1953.1.1.69●, Munigoon, Jumla District, Karnali Province; BMNH 1953.1.1.70●*, Hurikot, Dolpa District, Karnali Province; BMNH 1953.1.1.71* (paratype of *G. nepalensis* sp. nov.), Tibrikot, Dolpa District, Karnali Province; BMNH 1953.1.1.72*–73, Sialgarhi, Jumla District, Karnali Province; MHNG 1329.5*, Tukche, Mustang District, Gandaki Province; MHNG 1329.6* (paratype of *G. nepalensis* sp. nov.), MHNG 1329.7 and MHNG 1329.8* (paratype of *G. nepalensis* sp. nov.), Dhorpatan, Baglung District, Gandaki Province; NHMK 254 (formerly RMNH.RENA 20514) (paratype of *G. nepalensis* sp. nov.), adult male from Gurja Ghat, 5 km east of Dhorpatan, Baglung District, Gandaki Province; NME R 0543/07* (paratype of *G. nepalensis* sp. nov.), Simikhola, Humla District, Karnali Province; NME R 0544/07*, Kermi village, Humla District, Karnali Province; RMNH.RENA.20512●* (paratype of *G. nepalensis* sp. nov.), Tal village, Manang District, Gandaki Province; RMNH.RENA.20513* (paratype of *G. nepalensis* sp. nov.), Dhorpatan, Baglung District, Gandaki Province; ZMB 65606–65610, ZMB 65611●, ZMB 65612●* (paratype of *G. nepalensis* sp. nov.), Syang village (Jomsom,) Mustang District, Gandaki Province; ZMB 65613●* (holotype of *G. nepalensis* sp. nov.), Kalopani village, Mustang District, Gandaki Province; ZSM 154/1973/1●*–2* (paratypes of *G. nepalensis* sp. nov.), ZSM 154/1973/4* (paratype of *G. nepalensis* sp. nov.), ZSM 156/1973/1*(paratype of *G. nepalensis* sp. nov.), ZSM/1973/2*, ZSM 372/1981/1*, Kalopani village, Mustang District, Gandaki Province; ZSM 157/1973●* (paratype of *G. nepalensis* sp. nov.), Tukuche village, Mustang District, Gandaki Province; ZSM 330/1988*, Jumla, Jumla District, Karnali Province.

Table S2. Uncorrected genetic distance (p-distance) values for cytochrome *b* and ND4, calculated separately for all available *Gloydius* species and for the *G. himalayanus* complex. P-distances ≤ 3% are highlighted by red-shaded cells.

**Cyt *b***	*Gloydius* species	1	2	3	4	5	6	7	8	9	10	11	12	13	14	15	16	17	18	19	20	21	22	23	24	25	26	27	28	29
1	*angusticeps*																													
2	*blomhoffi*	11.3																												
3	*brevicaudus*	11.8	9.7																											
4	*caraganus*	9.4	9.8	12.7																										
5	*caucasicus*	9.9	10.6	12.9	3.8																									
6	*chambensis*	13.7	14.8	15.0	14.1	13.7																								
7	*changdaoensis*	9.6	10.4	12.9	5.0	5.1	13.5																							
8	*cognatus*	9.9	10.6	12.5	4.4	4.3	13.8	5.2																						
9	*halys*	9.2	9.5	12.6	3.6	3.9	14.0	4.4	3.9																					
10	*hazarensis* sp. nov.	13.4	15.0	15.1	13.4	12.6	10.6	14.5	13.2	13.8																				
11	*himalayanus*	12.6	13.7	14.6	12.7	12.7	11.3	12.9	12.8	12.9	11.4																			
12	*hindukushensis* sp. nov.	12.7	13.1	15.1	13.4	12.6	11.0	13.6	12.3	13.1	10.2	11.0																		
13	*huangi*	7.9	11.6	12.8	10.9	10.6	13.1	9.7	10.7	10.6	13.1	12.5	12.8																	
14	*intermedius*	9.4	9.7	13.1	3.4	3.5	14.0	4.4	3.7	0.4	14.4	13.1	13.6	10.4																
15	*lateralis*	8.9	10.0	11.9	9.3	9.1	13.4	9.5	8.7	9.0	13.2	12.6	12.1	9.7	9.1															
16	*lipipengi*	8.2	11.3	12.6	8.5	9.2	13.0	8.6	8.8	9.2	12.0	12.4	11.7	7.3	9.2	9.1														
17	*liupanensis*	8.4	11.6	12.3	8.9	8.8	12.7	9.8	9.4	9.1	12.4	13.1	12.3	9.4	9.0	8.9	8.4													
18	*monticola*	10.0	11.2	14.3	10.6	11.5	14.1	10.9	11.2	11.5	13.9	13.7	14.0	8.4	11.7	10.3	8.1	10.6												
19	*nepalensis* sp. nov.	13.7	13.9	14.5	14.2	13.5	11.2	14.2	14.0	14.2	12.6	9.5	12.1	13.4	14.0	13.5	13.4	13.1	14.8											
20	*qinlingensis*	9.2	9.3	11.0	8.5	8.8	13.1	9.3	9.0	8.7	12.5	11.8	13.2	8.2	9.0	7.9	7.7	8.0	9.8	13.5										
21	*rickmersi*	9.2	10.9	12.5	3.9	3.4	14.6	5.5	4.8	3.4	13.8	13.2	13.3	10.5	3.4	9.7	9.4	9.7	11.3	14.1	9.3									
22	*rubromaculatus*	8.6	10.2	11.5	8.7	8.8	13.1	9.3	8.9	8.7	12.3	12.5	12.8	7.8	9.0	8.5	5.7	8.1	9.1	13.9	4.0	9.2								
23	*shedaoensis*	10.4	11.2	13.4	4.1	4.6	15.0	5.6	4.7	3.4	15.2	14.3	14.4	11.4	3.7	9.9	10.0	10.0	12.1	14.8	9.7	5.0	9.8							
24	*stejnegeri*	9.5	10.1	12.5	3.6	3.7	14.3	4.9	3.9	2.6	13.7	13.4	13.6	10.9	2.8	9.2	9.3	8.8	10.5	14.8	9.2	3.7	9.1	4.0						
25	*strauchi*	9.1	9.5	11.1	8.6	8.9	13.2	9.7	9.2	8.8	12.7	12.1	13.3	8.2	9.2	7.6	7.6	8.1	9.9	13.9	0.6	9.4	3.7	9.8	9.3					
26	*swild*	9.2	9.0	11.8	7.8	8.1	12.3	8.1	8.4	7.8	13.3	12.8	12.2	9.0	7.9	6.7	8.4	8.1	10.2	13.6	7.6	8.8	8.0	8.6	8.4	7.5				
27	*tsushimaensis*	11.0	4.4	9.4	9.3	9.4	14.4	10.5	11.3	10.8	15.4	14.2	13.8	11.1	11.1	10.3	10.7	11.6	10.6	14.1	10.1	11.5	10.3	11.4	10.1	10.4	9.0			
28	*ussuriensis*	10.5	5.7	10.2	9.8	10.5	14.6	11.2	11.1	11.4	15.1	14.3	13.7	11.0	11.2	11.1	10.5	12.0	11.5	14.6	10.6	11.3	10.6	11.7	11.1	11.0	11.0	2.8		
29	*variegatus*	8.5	10.9	12.2	9.6	9.3	12.9	9.2	9.0	9.2	12.9	13.2	12.3	8.0	9.3	10.2	4.9	9.5	9.2	13.6	9.0	9.3	6.4	10.5	9.6	9.0	8.4	11.0	11.4	

ND4	*Gloydius* species	1	2	3	4	5	6	7	8	9	10	11	12	13	14	15	16	17	18	19	20	21	22	23	24	25	26	27	28	29
1	*angusticeps*																													
2	*blomhoffi*	8.6																												
3	*brevicaudus*	10.4	10.9																											
4	*caraganus*	9.2	10.5	13.4																										
5	*caucasicus*	10.1	11.1	14.2	4.8																									
6	*chambensis*	10.6	10.4	13.9	12.3	11.8																								
7	*changdaoensis*	9.4	10.9	14.3	4.4	5.8	12.7																							
8	*cognatus*	8.7	10.8	13.7	4.5	5.0	11.6	4.2																						
9	*halys*	8.9	11.6	12.4	4.4	4.7	11.8	4.3	3.8																					
10	*hazarensis* sp. nov.	13.0	13.1	14.4	13.4	13.2	11.6	13.9	13.7	13.0																				
11	*himalayanus*	11.4	11.7	14.4	11.7	12.4	9.6	12.6	11.9	11.9	14.1																			
12	*hindukushensis* sp. nov.	10.7	11.3	12.7	11.4	11.7	11.2	12.5	11.4	11.4	12.5	10.8																		
13	*huangi*	8.4	9.8	12.9	11.1	10.7	11.2	11.1	10.7	10.7	12.5	12.9	13.6																	
14	*intermedius*	8.7	11.4	12.5	4.2	4.6	11.7	4.1	3.6	0.1	13.0	11.8	11.5	10.5																
15	*lateralis*	8.4	11.1	13.8	8.7	9.9	11.8	8.9	9.5	9.2	13.2	12.6	12.2	9.5	9.0															
16	*lipipengi*	8.1	9.7	11.9	10.6	10.8	11.3	11.3	11.0	10.2	12.0	12.0	11.6	8.3	10.1	9.2														
17	*liupanensis*	7.6	9.1	11.2	8.4	9.0	10.8	9.0	8.7	8.6	12.5	12.5	10.6	9.3	8.4	7.5	8.1													
18	*monticola*	7.6	10.1	11.9	11.7	11.2	10.8	10.7	10.6	10.9	14.4	12.4	12.0	8.4	10.7	8.9	8.1	7.6												
19	*nepalensis* sp. nov.	9.6	10.8	12.2	11.3	10.6	10.3	11.1	10.6	10.8	11.9	9.6	8.4	11.8	10.7	11.9	11.2	10.6	9.6											
20	*qinlingensis*	6.1	9.4	11.1	9.8	9.8	10.6	9.8	9.5	9.6	13.6	11.5	11.0	7.8	9.5	8.7	7.2	6.7	7.3	9.4										
21	*rickmersi*	9.6	11.6	14.2	4.8	5.5	13.7	5.6	4.8	5.0	13.9	13.2	12.5	11.1	4.9	10.8	10.8	9.8	11.9	11.5	10.2									
22	*rubromaculatus*	7.1	9.2	11.4	10.0	9.8	10.9	9.7	9.7	9.3	13.0	11.7	11.2	7.8	9.2	8.6	5.6	7.9	7.7	9.9	4.0	10.0								
23	*shedaoensis*	10.1	11.1	14.6	4.4	5.2	12.5	4.1	4.1	4.0	12.7	11.8	12.2	11.4	3.8	10.1	11.1	9.2	11.5	11.5	10.8	4.7	10.5							
24	*stejnegeri*	8.9	10.6	13.6	4.2	4.1	11.5	4.1	3.0	3.2	13.2	11.5	11.7	10.7	3.1	9.2	10.2	8.3	10.4	10.1	9.3	4.4	9.2	3.4						
25	*strauchi*	6.5	9.2	11.4	9.8	9.6	10.7	9.4	9.3	9.4	12.9	11.7	11.4	8.0	9.2	8.5	7.8	6.5	7.3	9.9	3.5	10.3	5.8	10.3	8.9					
26	*swild*	8.3	9.1	12.2	9.2	9.0	10.3	9.7	9.8	9.0	12.9	11.7	12.1	9.3	8.8	5.6	8.3	7.2	7.5	10.5	8.6	10.2	8.0	10.2	8.1	8.1				
27	*tsushimaensis*	9.2	4.7	10.4	10.5	10.6	10.9	11.0	9.8	11.3	12.9	12.0	12.1	10.2	11.2	11.7	10.8	9.4	9.9	11.0	9.8	11.2	10.0	10.6	10.7	9.6	10.1			
28	*ussuriensis*	9.9	4.8	11.5	10.9	10.5	11.6	11.4	9.8	11.9	12.5	12.7	11.7	10.2	11.8	11.4	11.3	10.3	10.9	11.7	10.5	11.8	10.3	10.8	10.3	10.2	10.3	3.5		
29	*variegatus*	7.4	9.4	12.2	10.1	10.2	11.3	10.0	9.6	9.9	12.9	12.0	12.1	7.6	9.7	8.6	3.5	8.7	6.3	10.0	7.1	9.6	5.2	10.3	9.3	7.4	7.8	10.3	10.3	

Table S3. Gazetteer for the *Gloydius himalayanus* complex: Pakistan – India – Nepal. Remarks to Gazeteer: Coordinates in decimal format WGS84 were usually formed from the initial coordinates given in brackets below them. Status as of 22 September 2025.

No.	Locality name	Region	Country	Coordinates Decimal WGS84 (verbatim data)	Elevation (m)	Sources	Confirmed or estimated species affiliation
1	Chitral	Chitral District, Khyber Pakhtunkhwa Province	Pakistan	N 35.88333 E 71.80000 (35°53′N, 71°48′ E)	3048	Gloyd and Conant (1990); BMNH 1910.10.26.4–7; BNHS 2517	*hindukushensis* sp. nov.
2	Madaglasht [Madak Lasht], Chitral	Lower Chitral District, Khyber Pakhtunkhawa Province	Pakistan	N 35.77527 E 72.02555 (35°46´31´´N, 72°01´32´´E)	2650	Wall (1911b)	*hindukushensis* sp. nov.
3	Gilgit	Gilgit District, Gilgit-Baltistan Province	Pakistan	N 35.91694 E 74.30499 (35°55´01´´N, 74°18´18´´ E)	~1500	Sclater (1891); McMahon (1899); ZSIK 8774	*hindukushensis* sp. nov.
4	Chilas, Kashmir	Diamer District, Gilgit-Baltistan Province	Pakistan	N 35.42194 E 74.09861 (35°25´19´´N, 74°05´55´´E)	1180	Gloyd and Conant (1990); BNHS 2519	*hindukushensis* sp. nov.
5	Kumrat Valley	Upper Dir District, Khyber Pakhtunkhwa Province	Pakistan	N 35.56490 E 72.19583	2360	this study; NMW 41993 (formerly CUHC 10085), holotype of *Gloydius hindukushensis* sp. nov.; CUHC 10088 paratype of *G. hindukushensis* sp. nov.	*hindukushensis* sp. nov.
6	Dir	Upper Dir District, Khyber Pakhtunkhwa Province	Pakistan	N35.22333 E 71.86055 (35°13´24´´N, 71°51´38´´E)	1764–2119	Jamal et al. (2018)	*hindukushensis* sp. nov.
7	Dog Dara [Union Council]	Upper Dir District, Khyber Pakhtunkhwa Province	Pakistan	N 35.44388 E 72.32027 (35°26´38´´N, 72°19´13´´E)	2490–2888	Jamal et al. (2018)	*hindukushensis* sp. nov.
8	near Lal quilla (1)	Lower Dir District, Khyber Pakhtunkhwa Province	Pakistan	N 35.03375 E 71.86942	2446	this study; PMNH 5149 (p48); PMNH 5151	*hindukushensis* sp. nov.
9	near Lal quilla (2)	Lower Dir District, Khyber Pakhtunkhwa Province	Pakistan	N 34.98984 E 71.90301	2700	this study; PMNH 5150 (p21)	*hindukushensis* sp. nov.
10	Liakot	Swat District, Khyber Pakhtunkhwa Province	Pakistan	N 35.39333 E 72.59805 (35°23´36´´N, 72°35´53´´E)	2100	Minton (1966)	*hindukushensis* sp. nov.
11	Malam Jabba	Swat District, Khyber Pakhtunkhwa Province	Pakistan	N 34.80443 E 72.54806	1850	https://www.inaturalist.org/observations/170594199	*hindukushensis* sp. nov.
12	Torwal (Mankial)	Swat District, Khyber Pakhtunkhwa Province	Pakistan	N 35.23663 E 72.56742	1660	https://www.inaturalist.org/observations/174556294	*hindukushensis* sp. nov.
13	Northeast of Sappar	Swat District, Khyber Pakhtunkhwa Province	Pakistan	N 35.21403 E 72.63794	2380	https://www.inaturalist.org/observations/222464585	*hindukushensis* sp. nov.
14	Breathbeck, Bar Balas	Kolai-Palas District, Khyber Pakhtunkhwa Province	Pakistan	N 34.98972 E 73.09361 (34°59´23´´N, 73°05´37´´E)	2400–2650	Showler (1998)	*hazarensis* sp. nov.
15	Satoe, Bar Palas	Kolai-Palas District, Khyber Pakhtunkhwa Province	Pakistan	N 34.99583 E 73.04583 (34°59´45´´N, 73°02´45´´E)	2400	Showler (1998)	*hazarensis* sp. nov.
16	Dhar (above Kundal)	Kolai-Palas District, Khyber Pakhtunkhwa Province	Pakistan	N 35.00166 E 73.00583 (35°00´06´´N, 73°00´21´´E)	2750	Showler (1998)	*hazarensis* sp. nov.
17	above Shukiser [Sukkhi Ser]	Kolai-Palas District, Khyber Pakhtunkhwa Province	Pakistan	N 35.02194 E 73.22333 (35°01´19´´N, 73°13´24´´E)	2500	Showler (1998)	*hazarensis* sp. nov.
18	Par Palas [Bar Palas]	Kolai-Palas District, Khyber Pakhtunkhawa Province	Pakistan	N 34.96888 E 72.99083 (34°58´08´´N, 72°59´27´´E)	2500-2700	this study; PMNH 0396	*hazarensis* sp. nov.
19	Kel, Neelum River Valley	Neelam District, Azad Jammu and Kashmir, Pakistan-administered region	Pakistan	N 34.83525 E 74.35781	2256	this study; PMNH 0318	*hazarensis* sp. nov.
20	Badi (Large), Gagai Nala, Upper Taobat village, Neelum River Valley	Neelam District, Azad Jammu and Kashmir, Pakistan-administered region	Pakistan	N 34.74781 E 74.74631	2385	this study; PMNH 1979	*hazarensis* sp. nov.
21	Thaoba [Taobat] at Kishengunga [Kishanganga/Neelum] river	Neelam District, Azad Jammu and Kashmir, Pakistan-administered region	Pakistan	N 34.72555 E 74.71194 (34°43´32´´N, 74°42´43´´E)	~2300	Fenton (1910)	*hazarensis* sp. nov.
22	Rawalakot	Poonch District, Azad Jammu and Kashmir, Pakistan-administered region	Pakistan	N 33.85333 E 73.75138 (33°51′12″N 73°45′05″E)	~1630	Shabir (2022)	*hazarensis* sp. nov.
23	Sharan (Manchi Forest, Kaghan River Valley)	Mansehra District, Khyber Pakhtunkhwa Province	Pakistan	N 34.70485 E 73.43649	2463	this study; PMNH 4109; PMNH 4110; Masroor (2017)	*hazarensis* sp. nov.
24	Hazara [Region]	Mansehra District, Khyber Pakhtunkhwa Province	Pakistan	N 34.89861 E 73.64583 (34°53´55´´N, 73°38´45´´E)	2400	Unwin in Lawrence (1895)	*hazarensis* sp. nov.
25	3.3 km S Naran, Kaghan River Valley, 2200 m	Mansehra District, Khyber Pakhtunkhawa Province	Pakistan	N 34.88055 E 73.62722 (34°52´50´´N, 73°37´38´´E)	~2200	Telford (1980); UF 70652, holotype of *Gloydius hazarensis* sp. nov.: UF 70651, UF70653–70675, UF 119811-12	*hazarensis* sp. nov.
26	Shogren [Shogran]	Mansehra District, Khyber Pakhtunkhwa Province	Pakistan	N 34.64111 E 73.46361 (34°38´28´´N, 73°27´49´´E)	2350	USNM 153757	*hazarensis* sp. nov.
27	Shogran	Mansehra District, Khyber Pakhtunkhwa Province	Pakistan	N 34.63806 E 73.46258	2364	Hadi and Junaid (2024)	*hazarensis* sp. nov.
28	Nathiagali [Nathia Gali]	Abbottabad District,	Pakistan	N 34.07305 E 73.38111 (34°04′23´´N, 73°22´52´´E)	2450	Khan and Tasnim (1986); UF 82634	*hazarensis* sp. nov.

		Khyber Pakhtunkhawa Province					
29	Thundiana [Thandiani]	Abbottabad District, Khyber Pakhtunkhwa Province	Pakistan	N 34.23333 E 73.35000 (34°14′N 73°21′E)	2750	Gloyd and Conant (1990); BNHS 2504	*hazarensis* sp. nov.
30	Makra Top, Kotla River Valley, Machiara National Park	Bagh District, Azad Jammu and Kashmir, Pakistan-administered region	Pakistan	N 34.56255 E 73.61289	2900	this study; PMNH 1655	*hazarensis* sp. nov.
31	Mari or Marri [Murree]	Murree District, Punjab Province	Pakistan	N 33.90416 E 73.39027 (33°54′15″N, 73°23′25″E)	2300	Blanford (1878); Gloyd and Conant (1990); NMW 17078:1; ZSIK 8438	*hazarensis* sp. nov.
32	Ghora Galli, Punjab [Ghora Gali]	Muree District, Punjab Province	Pakistan	N 33.87638, E 73.33749 (33°52´35´´N 73°20‘15´´E)	1800	Gloyd and Conant (1990); BNHS 2515	*hazarensis* sp. nov.
33	Mughalabad	Rawalpindi District, Punjab Province	Pakistan	N 33.89680 E 73.42694	1738	this study; PMNH 4240	*hazarensis* sp. nov.
34	Forest Block SW of Badwan	Kupwara District, Jammu and Kashmir State	India	N 34.61474 E 74.77113	3520	https://www.inaturalist.org/observations/129777918	*hazarensis* sp. nov., *chambensis* or *himalayanus*
35	Gurys [Gurez or Gurais]	Bandipora District, Jammu and Kashmir State	India	N 34.63330 E 74.83330	2580	Vigne (1842: 213, footnote).	*hazarensis* sp. nov., *chambensis* or *himalayanus*
36	West of Shadi Pora	Bandipora District, Jammu and Kashmir State	India	N 34.17523 E 74.66518	1600	https://www.inaturalist.org/observations/228418939	*hazarensis* sp. nov., *chambensis* or *himalayanus*
37	Bakhtaor beyond Kauzilwán [Kazalwan] on the Gilgit road	Bandipora District, Jammu and Kashmir State	India	N 34.64361 E 74.69944 (34°38´27´´N, 74°41´58´´E)	2400	Fenton (1910)	*hazarensis* sp. nov., *chambensis* or *himalayanus*
38	Erin nala [Erin Nallah (river); Erin Nar]	Bandipora District, Jammu and Kashmir State	India	N 34.41027 E 74.72333 (34°24´37´´N, 74°43´24´´E)	~1800	Fenton (1910)	*hazarensis* sp. nov., *chambensis* or *himalayanus*
39	Gilgit road between Karagbal [Koragbal/Korgbal, India] and Burzil [Pakistan], 8,000 ft.	Bandipora District, Jammu and Kashmir State	India	N 34.64694 E 74.71888 (34°38´49´´N, 74°43´08´´E)	~2440	Alcock (1898)	*hazarensis* sp. nov., *chambensis* or *himalayanus*
40	Gulmerg [Gulmarg]	Baramulla District, Jammu and Kashmir State	India	N 34.05000 E 74.38333 (34°4'N, 74°23' E)	2700	Gloyd and Conant (1990); BMNH 1896.11.20.7–8	*hazarensis* sp. nov., *chambensis* or *himalayanus*
41	Kashmir (Cashmere) valley	Srinagar District, Jammu and Kashmir State	India	N 34.06888 E 74.72027 (34°04´08´´N, 74°43´13´´E)	1700	Anonymous (1884); Scalter (1891, ZSIK 12602)	*hazarensis* sp. nov., *chambensis* or *himalayanus*
42	Sirinapur [Srinagar]	Srinagar District, Jammu and Kashmir State	India	N 34.09000 E 74.78999 (34°5′24″N, 74°47′24″E)	1730	Steindachner (1869, NMW 17079:1-2); Gloyd and Conant (1990); BNHS 2518; USNM 20883–20884	*chambensis* (according to genetic data of NMW 17079:1)
43	Dachigaon [Dachigam], 1895 m	Srinagar District, Jammu and Kashmir State	India	N 34.12444 E 74.94694 (34°07´28´´N, 74°56´49´´E)	1895	Sahi and Duda (1985)	*hazarensis* sp. nov., *chambensis* or *himalayanus*
44	between Sonamurg [Sonamarg] and Kharbu, Ladak	Ganderbal District, Jammu and Kashmir State	India	N 34.30027 E 75.29138 (34°18´01´´N, 75°17´29´´E)	~2700	Anonymous (1872); Sclater (1891); ZSIK 8573	*hazarensis* sp. nov., *chambensis* or *himalayanus*
45	East of Sonamarg	Ganderbal District, Jammu and Kashmir State	India	N 34.29688 E 75.31261	2700	https://www.inaturalist.org/observations/228404609	*hazarensis* sp. nov., *chambensis* or *himalayanus*
46	Darakyja Mountain (Pir Panjal Range)	Poonch District, Jammu and Kashmir State	India	N 33.62993 E 74.51997	3490	Minton (1966), Khan and Tasnim (1986); Gloyd and Conant (1990); AMNH 39395	*hazarensis* sp. nov., *chambensis* or *himalayanus*
47	vicinity of Poonch [Punch], ca. 1000 m	Poonch District, Jammu and Kashmir State	India	N 33.77030 E 74.09250	~1000	Murthy and Sharma (1976)	*hazarensis* sp. nov., *chambensis* or *himalayanus*
48	Palawar [Pulwama]	Pulwama District, Jammu and Kashmir State	India	N 33.86666 E 74.90000 (33°52'N, 74°54'E)	1639	Gloyd and Conant (1990); USNM 48471-48473	*hazarensis* sp. nov., *chambensis* or *himalayanus*
49	above Kollur [Kular], Siddar [Liddar] River Valley	Anantnag District, Jammu and Kashmir State	India	N 33.91916 E 75.25250 (33°55´09´´ N, 75°15´09´´E)	2200	Unwin in Lawrence (1895)	*hazarensis* sp. nov., *chambensis* or *himalayanus*
50	above Batkot (Liddar River Valley)	Anantnag District, Jammu and Kashmir State	India	N 33.94036 E 75.29936	2040	https://www.inaturalist.org/observations/197612133	*hazarensis* sp. nov., *chambensis* or *himalayanus*
51	Goojar [Gurjar [Shepherd] village], Siddar [Liddar] River Valley [Lidderwat]	Anantnag District, Jammu and Kashmir State	India	N 34.15833 E 75.24138 (34°09´30´´ N, 75°14´29´´E)	2770	Fenton (1910)	*hazarensis* sp. nov., *chambensis* or *himalayanus*
52	Mundlan [Mondlan], Siddar [Liddar] River Valley [near Pahalgam]	Anantnag District, Jammu and Kashmir State	India	N 34.04555 E 75.29611 (34°02´44´´N, 75°17´46´´E)	2250	Fenton (1910)	*hazarensis* sp. nov., *chambensis* or *himalayanus*
53	South of Mondlan	Anantnag District, Jammu and Kashmir State	India	N 34.04222 E 75.29683	2200	https://www.inaturalist.org/observations/226457893	*hazarensis* sp. nov., *chambensis* or *himalayanus*
54	11 km E Batkote near Pahlgam [Pahalgam]	Anantnag District, Jammu and Kashmir State	India	N 34.01722 E 75.30749 (34°01´02´´N, 75°18´27´´E)	2300	Dattatri (1985); Gloyd and Conant (1990)	*hazarensis* sp. nov., *chambensis* or *himalayanus*
55	near Chandanwari (9,000 ft.)	Anantnag District, Jammu and Kashmir State	India	N 34.02361 E 75.29638 (34°01´25´´N 75°17´47´´E)	2740	Ahmad (1946)	*hazarensis* sp. nov., *chambensis* or *himalayanus*
56	around Pahlgam [Pahalgam], Lidder River Valley, 7,500 ft.	Anantnag District, Jammu and Kashmir State	India	N 34.01444 E 75.32444 (34°00´52´´N, 75°19´28´´E)	2280	Wall (1899), Sahi (1979)	*hazarensis* sp. nov., *chambensis* or *himalayanus*
57	Buj Marg, Siddar [Lidder] River Valley above Páhlgám [Pahalgam]	Anantnag District, Jammu and Kashmir State	India	N 34.033055 E 75.31027 (34°01´59´´N, 75°18´37´´E)	2250	Fenton (1910)	*hazarensis* sp. nov., *chambensis* or *himalayanus*
58	Hajan [Betaab] Valley south of Phraslan	Anantnag District, Jammu and Kashmir State	India	N 34.05406 E 75.36388	2400	https://www.inaturalist.org/observations/206754496	*hazarensis* sp. nov., *chambensis* or *himalayanus*
59	Aishmugam [Aishmuquam]	Anantnag District, Jammu and Kashmir State	India	N 33.86138 E 75.28500 (33°51´41´´N, 75°17´06´´E)	1900	Dattatri (1985)	*hazarensis* sp. nov., *chambensis* or *himalayanus*
60	Kishtwar, 1670 m	Kishtwar District, Jammu and Kashmir State	India	N 33.30666 E 75.77333 (33°18´24´´N, 75°46´24´´E)	1670	Sahi (1979); Sahi and Duda (1985)	*hazarensis* sp. nov., *chambensis* or *himalayanus*

61	Yurod	Kishtwar District,<br>Jammu and Kashmir State	India	N 33.82998 E 75.55240	2550	https://www.inaturalist.org/observations/167171063	*hazarensis* sp. nov., *chambensis* or *himalayanus*
62	Doda City	Doda District,<br>Jammu and Kashmir State	India	N 33.10955 E 75.54648<br>(33°08'34.41´´N, 75°32'47.33´´E)	980–1280	Manhas et al. (2018)	*hazarensis* sp. nov., *chambensis* or *himalayanus*
63	Zatinda	Doda District,<br>Jammu and Kashmir State	India	N 33.09291 E 75.63881<br>(33°5'34.48´´N, 75°38'19.74´´E)	1600–2300	Manhas et al. (2018);<br>Manhas (2020)	*hazarensis* sp. nov., *chambensis* or *himalayanus*
64	Nai-Bhallara	Doda District,<br>Jammu and Kashmir State	India	N 33.08908, E 75.70840<br>(33°05'20.69´´N, 75°42'30.24´´E)	1700–2300	Manhas et al. (2018);<br>Manhas (2020)	*hazarensis* sp. nov., *chambensis* or *himalayanus*
65	Bhaderwah, 1650 m<br><br>Bhaderwah	Doda District,<br>Jammu and Kashmir State	India	N 32.98500 E 75.70750<br>(32°59´06´´N, 75°42´27´´E)<br>N 32.97996 E 75.71591<br>(32°58'47.89´´N, 75°42'57.27´´E)	1650<br><br>1500–1800	Sahi (1979); Sahi and Duda (1985); Manhas et al. (2018); Manhas (2020)	*hazarensis* sp. nov., *chambensis* or *himalayanus*
66	Ramnagar, 800 m	Udhampur District,<br>Jammu and Kashmir State	India	N 32.80750 E 75.30861<br>(32°48´27´´N, 75°18´31´´E)	800	Sahi and Duda (1985)	*hazarensis* sp. nov., *chambensis* or *himalayanus*
67	Goduk [sic] (near Siachen Glacier, Karakorum Range according to Gloyd and Conant (1990), in error) [Ladak]	Ladakh	India	N 34.28416 E 75.96444<br>(provisional)	3000<br>(provisional)	Anderson (1872); ZSIK 3094	*hazarensis* sp. nov., *chambensis* or *himalayanus*
68	Dras	Kargil District, Ladakh	India	N 34.4325 E 75.75194<br>(34°25´57´´N, 75°45´07´´E)	~3100	Blanford (1878)	*hazarensis* sp. nov., *chambensis* or *himalayanus*
69	Bhanjraru	Chamba District,<br>Himachal Pradesh State	India	N 32.83909 E 76.14932	1738	Kuttalam et al. (2022), holotype of *Gloydius chambensis*: HARC R259	*chambensis*
70	Dumas, Bairagarh	Chamba District,<br>Himachal Pradesh State	India	N 32.90916 E 76.15000<br>(32°54´33´´N, 76°09´00´´E	1750	Kuttalam et al. (2022)	*himalayanus*
71	Khushnagri	Chamba District,<br>Himachal Pradesh State	India	N 32.85694 E 76.15583<br>(32°51´25´´N, 76°09´21´´E)	1670	Kuttalam et al. (2022)	*himalayanus*
72	Tharvai [Tarwai]	Chamba District,<br>Himachal Pradesh State	India	N 32.88944 E 76.17500<br>(32°53´22´´N, 76°10´30´´E)	1500	Kuttalam et al. (2022)	*himalayanus*
73	Bairagarh	Chamba District,<br>Himachal Pradesh State	India	N 32.89972 E 76.16250<br>(32°53´59´´N, 76°09´45´´E)	2230	Kuttalam et al. (2022)	*himalayanus*
74	Kalatop workstation, Khajjiar	Chamba District,<br>Himachal Pradesh State	India	N 32.55055 E 76.01861<br>(32°33´02´´N, 76°01´07´´E)	2400	Kuttalam et al. (2022, ZSI Solan)	*himalayanus*
75	Dalhousi (Silverton)	Chamba District,<br>Himachal Pradesh State	India	N 32.53333 E 75.95000<br>(32°32´ N 75°57´ E)	1800–2050	Boyd (1910); Gloyd and Conant (1990); BNHS 2507; BNHS 2508; BNHS 2513; BNHS 2514	*himalayanus* or *chambensis*
76	Chamba, Pangi [Ravi] River Valley	Chamba District,<br>Himachal Pradesh State	India	N 32.55694 E 76.12777<br>(32° 33′ 25´´N, 76°07'40´´E)	~1000	Gloyd and Conant (1990); BMNH 1898.5.17.4–5	*chambensis*
77	Teppa	Chamba District,<br>Himachal Pradesh State	India	N 32.93870 E 76.25076	2543	G. Choure pers. comm. 2024	*chambensis*
78	Bakloh	Chamba district,<br>Himachal Pradesh State	India	N 32.46549 E 75.92562	1400	Gloyd and Conant (1990); BMNH 1930.5.8.972 (osteol. prep.)	*himalayanus* or *chambensis*
79	Northwest of Jiyoti (Sundrani Dhar)	Chamba district,<br>Himachal Pradesh State	India	N 32.52731 E 76.23604	3000	https://www.inaturalist.org/observations/226249154	*himalayanus* or *chambensis*
80	below Tirund	Kangra District,<br>Himachal Pradesh State	India	N 32.26909 E 76.33228	2300	https://www.inaturalist.org/observations/14102201	*himalayanus*
81	Talampur [Palampur]	Kangra District,<br>Himachal Pradesh State	India	N 32.11527 E 76.53999<br>(32°06´55´´N, 76°32´24´´E)	~1300	Gloyd and Conant (1990); BNHS 2510; Kuttalam et al. (2022)	*himalayanus*
82	Dharmsala, at foot of the glacier, 16,000 ft.	Kangra District,<br>Himachal Pradesh	India	N 32.24880, E 76.47694<br>(provisional)	4600<br>(provisional)	Sclater (1891); ZSIK 12875	*himalayanus*
83	Sainj	Kullu District,<br>Himachal Pradesh State	India	N 31.07916 E 77.39166<br>(31°04´45´´N, 77°23´30´´E)	1320	Kuttalam et al. (2022)	*himalayanus*
84	Banjar	Kullu District,<br>Himachal Pradesh	India	N 31.65172 E 77.38610	1650	https://www.inaturalist.org/observations/126067080	*himalayanus*
85	North of Nasogi	Kullu District,<br>Himachal Pradesh State	India	N 32.23690 E 77.16539	2660	https://www.inaturalist.org/observations/7352766	*himalayanus*
86	Northeast of Nasogi	Kullu District,<br>Himachal Pradesh State	India	N 32.24209 E 77.17167	2240	https://www.inaturalist.org/observations/167759180	*himalayanus*
87	Rolla campsite, Great Himalayan National Park	Kullu District,<br>Himachal Pradesh State	India	N 31.67127 E 77.48247	2090	https://www.inaturalist.org/observations/163473108	*himalayanus*
88	Shangarh	Kullu District,<br>Himachal Pradesh State	India	N 31.74434 E 77.39560	2150	https://www.inaturalist.org/observations/111514301	*himalayanus*
89	Mashyar	Kullu District,<br>Himachal Pradesh State	India	N 31.68576 E 77.51154	2440	https://www.inaturalist.org/observations/126768082	*himalayanus*
90	Chippni	Kullu District,<br>Himachal Pradesh State	India	N 31.68576 E 77.51154	2000	https://www.inaturalist.org/observations/162634652	*himalayanus*
91	East of Bahang, Kugti Wildlife Sanctuary	Kullu District,<br>Himachal Pradesh State	India	N 32.27042 E 77.19270	2320	https://www.inaturalist.org/observations/61759250	*himalayanus*
92	Bashisht	Kullu District,<br>Himachal Pradesh State	India	N 32.27339 E 77.18990	2240	https://www.inaturalist.org/observations/62041186	*himalayanus*
93	Deohari	Kullu District,<br>Himachal Pradesh State	India	N 31.76553 E 77.32880	1960	https://www.inaturalist.org/observations/100611193	*himalayanus*

94	Manali	Kullu District, Himachal Pradesh State	India	N 32.24317 E 77.18924	2050	Gloyd and Conant (1990); BNHS 2511; Captain in Gumprecht et al. (2004)	*himalayanus*
95	East of Larankelo	Kullu District, Himachal Pradesh State	India	N 32.08884 E 77.15596	1720	https://www.inaturalist.org/observations/251050204	*himalayanus*
96	Old Manali	Kullu District, Himachal Pradesh State	India	N 32.25503 E 77.16969	2060	https://www.inaturalist.org/observations/179477574	*himalayanus*
97	West Manali	Kullu District, Himachal Pradesh State	India	N 32.24501 E 77.17571	2120	https://www.inaturalist.org/observations/99058174	*himalayanus*
98	South of Manali	Kullu District, Himachal Pradesh State	India	N 32.22358 E 77.17995	2100	https://www.inaturalist.org/observations/238990292	*himalayanus*
99	Bashisht	Kullu District, Himachal Pradesh State	India	N 32.27339 E 77.18990	2250	https://www.inaturalist.org/observations/62041186	*himalayanus*
100	Kulu/Kooloo (Valley)	Kullu District, Himachal Pradesh State	India	N 32.58333 E 77.18333 (31.95°N 77.11°E)	1300	Gloyd and Conant (1990); BNHS 2505; MCZ R3138–MCZ R3140; MCZ R3143; MCZ R3149–3150; MCZ R3227; MCZ R3230; MCZ R4023; MCZ R4800; MCZ R 179596–179597; MCZ R 179599–179606; UMMZ 50086	*himalayanus*
101	Maggar [Nagar] Kulu [Naggar-Kulu]	Kullu District, Himachal Pradesh State	India	N32.13805 E 77.17388 (32°8'17´´N, 77°10'26´´E)	~1700	Chabanaud (1922), Gloyd and Conant (1990); MNHN-RA-1919.25	*himalayanus*
102	Naggar Nala, on the road from Naggar to Manali, ca. 6,200 ft., Kula district	Kullu District, Himachal Pradesh State	India	N 32.16833 E 77.19305 (32°10´06´´N, 77°11´35´´E)	1890	Acharji and Kripalani (1952)	*himalayanus*
103	Katrain (Kulu)	Kullu District, Himachal Pradesh State	India	N 32.13166 E 77.12388 (32°07´54´´N, 77°07´26´´E)	2300	Ahmad (1946)	*himalayanus*
104	Pulga	Kullu District, Himachal Pradesh State	India	N 31.99654 E 77.44078 (31°59´47.56´´N, 77°26´26.82´´E)	2210	Chabanaud (1922); MNHN-RA-1916.110	*himalayanus*
105	above Pangi, Chini [Rekong Peo] 10,000 ft.	Kinnaur District, Himachal Pradesh State	India	N 31.59777 E 78.27805 (31°35´52´´N, 78°16´41´´E)	3048	Stoliczka (1866)	*himalayanus*
106	Baspa (Sangla) River Valley [Rakcham to Mastarang]	Kinnaur District, Himachal Pradesh State	India	N 31.37450 E 78.37415 (31°22´28.23´´N 78°22´26.93´´E)	3050–3300	Negi and Banyal (2016)	*himalayanus*
107	Simla [Shimla]	Shimla District, Himachal Pradesh State	India	N 31.10333 E 77.17222 (31°6´12´´N, 77°10´20´´E)	~2100	Steindachner (1869, NMW17077: 1–2); Anderson (1871); Anonymous (1889 ex ZSIK coll., now ROM 0061 osteol. prep.); Hubrecht (1882); Sclater (1891); BMNH 1931.2.4.4; BNHS 2506; BNHS 2509; RMNH.RENA.4096 A–C; ZSIK 13189	*himalayanus*
108	upper Rampur [Rampur Bushahr]	Shimla District, Himachal Pradesh State	India	N 31.45000 E 77.63305 (31°27´0´´N, 77°37´59´´E)	1020	ZMA.RENA.16233	*himalayanus*
109	Manju	Shimla District, Himachal Pradesh State	India	N 31.16138 E 77.22611 (31°09´41´´N, 77°13´34´´E)	1700	Kuttalam et al. (2022)	*himalayanus*
110	Dhevu [Dheu?], near Sarahan	Shimla District, Himachal Pradesh State	India	N 31.48694 E 77.78194 (31°29´´13´´N, 77°46´55´´E)	2000	Kuttalam et al. (2022)	*himalayanus*
111	Kotgur [Kothgar],	Shimla District, Himachal Pradesh State	India	N 31.51666 E 77.78333 (31.31°N, 77.47°E)	~1900	NMW 15134:1–3; SMF 32783; ZMB 2940; ZMB 65963	*himalayanus*
112	Hatu Mountain near Kotgur [Kothgar], 10,000 ft.	Shimla District, Himachal Pradesh State	India	N 31.24833 E 77.49916 (31°14´54´´N, 77°29´57´´E)	3048	Stoliczka (1870), Fayrer (1874)	*himalayanus*
113	Beas River Valley NW of Simla [Shimla], 6000-8000 ft.	Shimla District, Himachal Pradesh State	India	N 31.72055 E 76.92055 (provisional)	1828-2438 (provisional)	Steindachner (1869, NMW)	*himalayanus*
114	NW of Simla [Shimla]	Shimla District, Himachal Pradesh State	India	N 31.11805 E 77.13944 (31°07´05´´N, 77°08´22´´E)	1982	Anonymous (1869, ZSIK)	*himalayanus*
115	Baghivia Narbandh, Simla Hills	Shimla District, Himachal Pradesh State	India	N 31.10888 E 77.17361 (31°06´32´´N, 77°10´25´´E)	1960	Gloyd and Conant (1990); BNHS 2512	*himalayanus*
116	near Matiana, 9000 ft.	Shimla District, Himachal Pradesh State	India	N 31.21666 E 77.40361 (31°13´00´´N 77°24´13´´E)	2743	Wall (1907)	*himalayanus*
117	East of Tarna	Shimla District, Himachal Pradesh State	India	N 30.81250 E 77.45971	2060	https://www.inaturalist.org/observations/270846898	*himalayanus*
118	Cho [Chor, Chur or Tschur] Mountain 8,000 ft.	Simaur District, Himachal Pradesh State	India	N 30.83722 E 77.45277 (30°50´14´´N, 77°27´10´´E)	2438	Anonymous (1879, ex ZSIK coll., now ROM 0049 osteol. prep.)	*himalayanus*
119	Serahan [Sarahan], 10,000 ft.	Simaur District, Himachal Pradesh State	India	N 31.49333 E 77.80666 (31°29´36´´N, 77°48´24´´E)	3048	Stoliczka (1870), Fayrer (1874)	*himalayanus*
120	Renuka wetland area	Simaur District, Himachal Pradesh State	India	N 30.61444 E 77.45500 (30°36´52´´N 77°27´18´´E)	~900	Metha (2000)	*himalayanus*
121	Chakrata	Dehradun District, Uttarakhand State	India	N 30.69916 E 77.87138 (30°41´57´´N, 77°52´017´´E)	2150	Gleadow (1899)	*himalayanus*
122	Mussoorie [Masuri]	Dehradun District, Uttarakhand State	India	N 30.46416 E 78.06555 (30°27´51´´N, 78°03´56´´E)<br><br>N 30.45807 E 78.10173	1900<br><br>2250	Sclater (1891); Gloyd and Conant (1990); BMNH 1905.10.27.4; ZSIK 13248<br><br>https://www.inaturalist.org/observations/186251208	*himalayanus*
123	Benog	Dehradun District, Uttarakhand State	India	N 30.47480 E 78.01646 (30°28´29.28´´N 78°00´59.26´´E)	2200	this study, WII-ADR199	*himalayanus*
124	Landour	Dehra Dun District, Uttarakhand State	India	N 30.45401 E 78.10380	2130	https://www.inaturalist.org/observations/102400311	*himalayanus*
125	Dehra Dun [Dehradun]	Dehradun District, Uttarakhand State	India	N 30.33333 E 78.05000 (30°20'N, 78°03'E)	670	SMNS 4374; Bahuguna (2010)	*himalayanus*
126	Timli	Dehradun District, Uttarakhand State	India	N 30.25805 E 77.51444 (uncertain) (30°15´29´´N, 77´30´52´´E)	850 (uncertain)	Hallermann et al. (2001); ZMH R04857	*himalayanus*

127	W of Gangnani (10600 ft.)	Uttarakhashi District, Uttarakhand State	India	N 30.92100 E 78.64500	3231	https://www.inaturalist.org/observations/189127966	*himalayanus*
128	Gangotri near Bhagirati River	Uttarakhashi District, Uttarakhand State	India	N 30.99469 E 78.93984	3060	https://www.inaturalist.org/observations/4913628	*himalayanus*
129	W of Kalkatiadhar (Sankari Range)	Uttarakhashi District, Uttarakhand State	India	N 31.12731 E 78.37283	2880	https://www.inaturalist.org/observations/105774666	*himalayanus*
130	between Bhebra camp site and Manjhi (Baharat Range)	Uttarakhashi District, Uttarakhand State	India	N 30.84722 E 78.49777 (30°50´50´´N, 78°29´52´´E)	2400	this study (not collected)	*himalayanus*
131	Taluka	Uttarakhashi District, Uttarakhand State	India	N 31.07812 E 78.24547	2100	this study, WII-ADR2023	*himalayanus*
132	Garhval [Garhwal Region]	Uttarkashi District, Uttarakhant State	India	N 31.11833 E 78.34388 (31°07´06´´N, 78°20´38´´E) provisional coordinates, see the	2743	Günther (1864), lectotype of *Halys himalayanus*: BMNH 1946.1.19.64, see remarks on the lectotype in the species account; paralectotype of *Halys himalayanus* BMNH 1946.1.18.75; Theobald (1868); BNHS 2520	*himalayanus*
133	North of Kotgaon	Uttarakhashi District, Uttarakhand State	India	N 31.07770 E 78.16887	1900	https://www.inaturalist.org/observations/300569643	*himalayanus*
134	Dhanaulti (Kempty Range)	Theri Garhwal, Uttarakhand State	India	N 30.42054 E 78.24724	2240	https://www.inaturalist.org/observations/117869353	*himalayanus*
135	Nag Tiba [Nag Tibba, Serpent´s Peak]	Theri Garhwal District, Uttarakhand State	India	N 30.58583 E 78.09777 (30°35´09´´N, 78°09´05´´E)	~3000	Gloyd and Conant (1990); KU 129591	*himalayanus*
136	NE of Gaundhar, Ukhomath [=Ukhimath or Okhimath]	Rudraprayag District, Uttarakhand State	India	N 30.62241 E 79.20449	2820	https://www.inaturalist.org/observations/188985040	*himalayanus*
137	Guptakashi [Guptkashi], Garhwal Himalayas	Rudraprayag District, Uttarakhand State	India	N 30.52027 E 79.07527 (30°31´13´´N, 79°04´31´´E)	~1600	Gloyd and Conant (1990); BNHS 2885	*himalayanus*
138	Sokharak [Sau Kharak], Kedernath Wildlife Sanctuary	Rudraparyag District, Uttarakhand State	India	N 30.49138 E 79.20527 (30°29´29´´N 79°12´19´´E)	3050	Sharma (2004)	*himalayanus*
139	way towards Tungnath (Hindu shrine)	Rudraparyag District, Uttarakhand State	India	N 30.48833 E 79.20916 (30°29´18´´N 79°12´33´´E)	~3300	Sharma (2004)	*himalayanus*
140	W of Tungnath	Chamoli District, Uttarakhand State	India	30.48576, 79.22689	3460	https://www.inaturalist.org/observations/271927850	*himalayanus*
141	Molta	Chamoli District, Uttarakhand State	India	N 30.97222 E 77.96138 (30°58´20´´N, 77°57´41´´E)	3000	Hallermann et al. (2001) ZMH R04852–56, R04860, R04880	*himalayanus*
142	Sau Kharak	Chamoli District, Uttarakhand State	India	N 30.47890 E 79.21110	2850	this study, WII-ADS81	*himalayanus*
143	Bhyudar	Chamoli District, Uttarakhand State	India	N 30.66614 E 79.58843	2480	https://www.inaturalist.org/observations/3933748	*himalayanus*
144	Dhankuri, NW Kumaon [Dhakuri Pass]	Bageshwar District, Uttarakhand State	India	N 30.06444 E 79.91055 (30°03´52´´N, 79°54´38´´E)	3200	BMNH 1930.5.8.973	*himalayanus*
145	Dungair Dhar [ridge]	Pithorargarh District, Uttarakhand State	India	N 30.19516 E 80.27713 (30°11.710´´N, 80°16.628´´E)	3535	A. Captain pers. comm. (2024)	*himalayanus*
146	Lingurani	Pithorargarh District, Uttarakhand State	India	N 29.79527 E 80.06416 (29°47´43´´N 80°03´51´´E)	1280	A. Captain pers. comm. (2024)	*himalayanus*
147	Munsiari	Pithorargarh District, Uttarakhand State	India	N 30.07152 E 80.23734	2150	https://www.inaturalist.org/observations/180380111	*himalayanus*
148	Khaliya [Khulia] Danda	Pithorargarh District, Uttarakhand State	India	N 30.05947 E 80.19911 (30°03´34.1´´N 80°11´56.8´´E)	3433	A. Captain pers. comm. 2024; Vogel (2006)	*himalayanus*
149	Dhapa Bend	Pithorargarh District, Uttarakhand State	India	N 30.10891 E 80.24627 (30°06´32.1´´N, 80°14´46.6´´E)	1794	A. Captain pers. comm. (2024)	*himalayanus*
150	Dansi Dhar	Pithorargarh District, Uttarakhand State	India	N 30.43222 E 80.46722 (30°10´956´´N, 80°15´782´´E)	2640	A. Captain pers. comm. (2024)	*himalayanus*
151	NW of Paton	Pithorargarh District, Uttarakhand State	India	N 30.36055 E 80.41527 (30°11.638´´N E 80°16.535´´E)	3430	A. Captain pers. comm. (2024)	*himalayanus*
152	NE of Maheshwar Kund	Pithorargarh District, Uttarakhand State	India	N 30.08345 E 80.23096	2340	https://www.inaturalist.org/observations/192432488	*himalayanus*
153	Jalath	Pithorargarh District, Uttarakhand State	India	N 30.09829 E 80.24448	1920	https://www.inaturalist.org/observations/93158255	*himalayanus*
154	Sarmoli	Pithorargarh District, Uttarakhand State	India	N 30.07692 E 80.23314	2200	https://www.inaturalist.org/observations/87428538	*himalayanus*
155	Bheema Udiya	Pithorargarh District, Uttarakhand State	India	N 30.07705 E 80.23328	2200	https://www.inaturalist.org/observations/242928938	*himalayanus*
156	Binsar	Almora District Uttarakhand State	India	N 29.82973 E 79.68001	2043	Aitkinson (1884)	*himalayanus*
157	Kharkiya near Pindar Glacier	Almora District, Uttarakhand State	India	N 29.61273 E 79.49710	1620	https://www.inaturalist.org/observations/6774954	*himalayanus*
158	North of Khaprat	Naini Tal District, Uttarakhand State	India	N 29.43884 E 79.58847	2200	https://www.inaturalist.org/observations/234313158	*himalayanus*
159	Naini Tal	Naini Tal District, Uttarakhand State	India	N 29.39638 E 79.45111	~2100	Sclater (1891), ZSIK 4062-64	*himalayanus*
160	N of Khaprat	Naini Tal District, Uttarakhand State	India	N 29.44060 E 79.58891	2600	https://www.inaturalist.org/observations/55273209	*himalayanus*
161	Darshan viewpoint, Nainital	Nainital District, Uttarakhand State	India	N 29.40300 E 79.45100	2267	Dolia and Das (2023)	*himalayanus*
162	Van Niwas Ashram, Nainital	Nainital District, Uttarakhand State	India	N 29.38800 E 79.44200	2159	Dolia and Das (2023)	*himalayanus*
163	Tiffen Top, Nainital.	Nainital District, Uttarakhand State	India	N 29.38694 E 79.44222 (29°23´13´´N, 79°26´32´´E)	2215	Dolia and Das (2023)	*himalayanus*
164	Near Kabrar	Nainital District, Uttarakhand State	India	N 29.42822 E 79.59420	2360	https://www.inaturalist.org/observations/296249812	*himalayanus*
165	Chipa (Bhowali Range)	Naini Tal District, Uttarakhand State	India	N 29.43479 E 79.60408	2240	https://www.inaturalist.org/observations/184985751	*himalayanus*

166	N of Kharprat (Bowali Range)	Naini Tal District, Uttarakhand State	India	N 29.43675 E 79.59066	2200	https://www.inaturalist.org/observations/183939673	*himalayanus*
167	Mukteshwar	Naini Tal District, Uttarakhand State	India	N 29.460413 E 79.655824 (N 29.460413 E 79.655824)	2230	https://www.inaturalist.org/observations/161060496	*himalayanus*
168	W of Khaprat (Bhowali Range)	Naini Tal District, Uttarakhand Stated	India	N 29.43363 E 79.58193	2060	https://www.inaturalist.org/observations/185549876	*himalayanus*
169	Kermi, 14 km NW of Simikot	Humla District, Karnali Province	Nepal	N 30.04861 E 81.70722 (30°02'55´´N, 81°42'26´'E)	2800	this study; NME R 0544/07	*nepalensis* sp. nov.
170	NE of Simikot	Humla District, Karnali Province	Nepal	N 29.98329 E 81.79948	3220	https://www.inaturalist.org/observations/124481391	*nepalensis* sp. nov.
171	NW of Simikot	Humla District, Karnali Province	Nepal	N 29.98418 E 81.82245	3250	https://www.inaturalist.org/observations/248018481	*nepalensis* sp. nov.
172	Simikhola	Humla District, Karnali Province	Nepal	N 29.96972 E 81.813888 (provisional)	3000 (provisional)	this study; NME R 0543/07	*nepalensis* sp. nov.
173	Cher Tsaur	Jumla District, Karnali Province	Nepal	N 29.27490 E 82.18200	2900	ZSM 330/1988	*nepalensis* sp. nov.
174	Munigoon [Munigaun/Munigaon] near Jumla, 9500 ft.	Jumla District, Karnali Province	Nepal	N 29.21472 E 82.35055 (29°12´53´´N, 82°21´02´´E)	~2900	Smith and Battersby (1953), Gloyd and Conant (1990); BMNH 1953.1.1.69	*nepalensis* sp. nov.
175	Sialgarhi 9,000 to 10,000 ft. [near Chaudhabise Khola]<br><br>fide Shah (1995) =Silgadi [in error]	Jumla District, Karnali Province	Nepal	N 29.32944 E 82.36833 (29°19´46´´N, 82°22´06´´E)	2740–3048	Smith and Battersby (1953); Shah (1995); BMNH 1953.1.1.72–73	*nepalensis* sp. nov.
176	SW of Mangri	Mugu District, Karnali Province	Nepal	N 29.59139 E 82.29692	2200	https://www.inaturalist.org/observations/238210213	*nepalensis* sp. nov.
177	NE of Kimari	Mugu District, Karnali Province	Nepal	N 29.57349 E 82.47530	2530	https://www.inaturalist.org/observations/238210293	*nepalensis* sp. nov.
178	Hurikot, 10,000 ft. [Turikot]	Dolpa District, Karnali Province	Nepal	N 29.12111 E 82.60277 (29°07´16´´N, 82°36´10´´E)	3048	Smith and Battersby (1953); BMNH 1953.1.1.70	*nepalensis* sp. nov.
179	13 km W Tibrikot [Tripurakot], 8,500 ft.	Dolpa District, Karnali Province	Nepal	N 29.02833 E 82.83222 (29°01´42´´N, 82°49´56´´E)	~2600	Smith and Battersby (1953); BMNH 1953.1.1.71	*nepalensis* sp. nov.
180	Ghasa [Ghaza]	Mustang District, Gandaki Province	Nepal	N 28.64250 E 83.64250 (28°38´33´´N, 83°38´33´´E)<br>N 28.60503 E 83.64690 (28°36.302'N, 83°38.814'E)	2130<br><br>2105	Nanhoe and Ouboter (1987); BMNH 1955.1.13.80<br><br>S. Bhattarai (pers. com. 2024).	*nepalensis* sp. nov.
181	Lete	Mustang District, Gandaki Province	Nepal	N 28.61416 E 83.63333 (28°36´51´´N, 83°38´00´´E)	2440	Nanhoe and Ouboter (1987); BMNH 1955.1.13.79	*nepalensis* sp. nov.
182	Kalopani	Mustang District, Gandaki Province	Nepal	N 28.61666 E 83.60000 (28°37'N, 83°36'E)	2500	ZMB 65613, holotype of *Gloydius nepalensis* **sp. nov.**.; ZSM 154/1973/1–2, ZSM 154/1973/4, ZSM 156/1973/1–2, ZSM 372/1981/1	*nepalensis* sp. nov.
183	Titi Lake	Mustang District, Gandaki Province	Nepal	N 28.65243 E 83.61878 (28°39.146'N, 83°37.127'E)	2681	S. Bhattarai (pers. com. 2024)	*nepalensis* sp. nov.
184	Syang	Mustang District, Gandaki Province	Nepal	N 28.76666 E 83.70000 (28°46'N, 83°42'E)<br>N 28.77465 E 83.70720) (28°46.479´N, 83°42.433´E)	2700<br><br>2780	this study; ZMB 65606–65610, ZMB 65611, ZMB 65612.<br>S. Bhattarai (pers. com. 2024)	*nepalensis* sp. nov.
185	Marpha	Mustang District, Gandaki Province	Nepal	N 28.74277 E 83.68166 (28°44´34´´N, 83°40´54´´E)	2670	Sura (1987); M. Lorenz (pers. com. 1997); Schleich and Kästle (2002)	*nepalensis* sp. nov.
186	Tukche [Tukuche, Tukucha]	Mustang District, Gandaki Province	Nepal	N 28.70870 E 83.64480	2400–2700	Kramer (1977), Sura (1987); Nanhoe and Ouboter (1987); BMNH 1955.1.13.79; MHNG 1329.5; ZSM 157/1973	*nepalensis* sp. nov.
187	between Nilgiri Khola and Kali Gandaki [river]	Mustang District, Gandaki Province	Nepal	N 28.56211 E 83.66488 (N28°33'43.6", E83°39'53.6")	3242	this study; NME 070555	*nepalensis* sp. nov.
188	Dhorpatan	Baglung District, Gandaki Province	Nepal	N 28.49694 E 83.06138 (28°29´49´´N, 83°03´41´´E)	2400–3000	Kramer (1977); Nanhoe and Ouboter (1987); Shah (1995); MHNG 1329.6-8; RMNH.RENA.20513	*nepalensis* sp. nov.
189	Gurja Ghat	Myagdi District, Gandaki Province	Nepal	N 28.67916 E 83.21777 (28°36'285"N, 83°13'04"E)	3050	Nanhoe and Ouboter (1987); NHMK 254 (formerly RMNH.RENA.20514).	*nepalensis* sp. nov.
190	Gurjakhani [Gurja Khani]	Myagdi District, Gandaki Province	Nepal	N 28.58777 E 83.23666 (28°35´16´´N, 83°14´12´´E)	2590	Nanhoe and Ouboter (1987); BMNH 1955.1.13.83-93	*nepalensis* sp. nov.
191	Tachi Bagarchhap	Manang District, Gandaki Province	Nepal	N 28.53125 E 84.34355	2180	https://www.inaturalist.org/observations/16591776	*nepalensis* sp. nov.
192	Thonje	Manang District, Gandaki Province	Nepal	N 28.52368 E 84.35472	1850	https://www.inaturalist.org/observations/136471279	*nepalensis* sp. nov.
193	Goa [Gho]	Manang District, Gandaki Province	Nepal	N 28.5676 E 84.4039	2500	https://www.inaturalist.org/observations/246011439	*nepalensis* sp. nov.
194	Tal	Manang District, Gandaki Province	Nepal	N 28.45666 E 84.37555 (28°27´24´´N, 84°22´32´´E)	1640	Nanhoe and Ouboter (1987); Shah (1995); RMNH.RENA.20512	*nepalensis* sp. nov.

References used in gazetteer

Acharji MN, Kripalani MB (1952) On a collection of reptilia and batrachia from the Kangra and Kulu valleys, western Himalayas. Records of the Indian Museum 49: 175–184.

Ahmad N (1946) On a small collection of vertebrates from high altitudes in Kashmir State. Journal of the Asiatic Society of Bengal, Science 11(2) [1945]: 119–121.

Aitkinson ET (1884) The Himalayan Districts of the North-Western Provinces of India. Volume II (forming volume XI of the gazetteer, N.-W. P.). North-Western Province and Oudh Government Press, Allahabad, xvii, 964 pp.

Alcock AW (1898) Report on the natural history results of the Pamir Boundary Commission. Office of the Superintendent of Government Printing, Calcutta, [3], 45, [1] pp., 3 pls.

Anderson J (1871) A list of the reptilian accession to the Indian Museum, Calcutta, from 1865 to 1870, with a description of some new species. Journal of the Asiatic Society of Bengal 40, 11(1): 12–39.

Anderson J (1872) On some Persian, Himalayan, and other reptiles. Proceedings of the Zoological Society of London [1872]: 371–404.

Anonymous (1869) Accessions to the Museum in July 1869. Indian Museum Annual Report and lists of accessions: 1–79.

Anonymous (1872) Accessions to the Museum in May 1871. Indian Museum Annual Report and lists of accessions: 1–127.

Anonymous (1879) Accessions to the Indian Museum during the quarter ending 31st December 1878. Reptilia, Batrachia. Indian Museum. Annual Report and list of accessions: 1–161.

Anonymous (1884) Accessions to the Indian Museum during the quarter ending 31st December 1883. Reptilia. Indian Museum Annual Report and lists of accessions: 74–75.

Anonymous (1889) Accessions to the Indian Museum during the year 1888-89. Reptilia. Indian Museum Annual Report and lists of accessions: 36.

Bahuguna, A. (2010) Reptilia. In: Venkataraman, K. (Ed.), Fauna of Uttarakhand (Part-1) Vertebrates. State Fauna Series 18. Zoological Survey of India, Calcutta, 445–503.

Blanford WT (1878) Scientific results of the second Yarkand Mission; based upon the collections and notes made of the late Ferdinand Stoliczka. Part 6, Reptilia and Amphibia. Office of the Superintendent of Government Printing, Calcutta, 26 pp.

Boyd JEM (1910) Case of snake bite from Himalayan viper. Journal of the Bombay Natural History Society 20(1): 864–865.

Chabanaud P (1922) Mission Guy Barbault dans les provinces centrales de l'Inde et dans les région occidentale de l'Himalaya 1914. Résultats scientifiques. Reptiles et Batraciens. Paris, Blondel la Rougery, 15 pp., 2 pls.

Dattatri S (1985) In search of the Himalayan pit viper, and the legend of Zuhn Shah Sahib. Hamadryad 10 (1&2): 10–12.

Dolia J, Das A (2023) First record of mating and dichromatism in the Himalayan pitviper, *Gloydius himalayanus* (Günther, 1864). Reptiles & Amphibians 30: e18095: 1–4.

Fayrer J (1874) The Thanatophidia of India being a description of the venomous snakes of the Indian Peninsula with an account of the influence of their poison on life and a series of experiments. Second Edition revised and enlarged. London, J. and A. Churchill, viii, (1), 178 pp., 31 pls.

Fenton LL (1910) The snakes of Kashmir. Journal of the Bombay Natural History Society 19(4): 1002–1004.

Gleadow F (1899) Note on the Himalayan viper (*Ancistrodon himalayanus*). Journal of the Bombay Natural History Society 12(3): 577–578.

Gloyd HK, Conant R (1990) Snakes of the *Agkistrodon* complex - a monographic review. Society for the Study of Amphibians and Reptiles, Contributions to Herpetology no. 6, Oxford, Ohio, 614 pp.

Günther ACLG (1864) The reptiles of British India. Ray Society, London, xxvii, 452 pp., 28 pls.

Gumprecht A, Tillack F, Orlov NL Captain A, Ryabov S (2004) Asian Pitvipers. GeitjeBooks, Berlin, 368 pp.

Hadi A, Junaid A (2024) Natural History Notes: *Gloydius himalayanus* (Himalayan Pit-Viper). Diet. Herpetological Review 55: 588.

Hallermann J, Ananjeva NB, Orlov N L (2001) On a remarkable collection of reptiles and amphibians collected by the German Indian Expedition 1955–1958. Russian Journal of Herpetology 8(1): 59–68. https://doi.org/10.30906/1026-2296-2001-8-1-59-68

Hubrecht AAW (1882) Note XI. List of reptiles and amphibians brought by Mr. Francis Day. Notes from the Leyden Museum 4(2): 138–144.

Jamal Q, Idress M, Ullah S, Adnan M, Zaidi F, Zaman Q, Rasheed SB (2018) Diversity and altitudinal distribution of squamata in two distinct ecological zones of Dir, a Himalayan sub-zone of northern Pakistan. Pakistan Journal of Zoology 50(5): 1835–1839. http://dx.doi.org/10.17582/journal.pjz/2018.50.5.1835.1839

Khan MS, Tasnim R (1986) Notes on the Himalayan pit viper, *Agkistrodon himalayanus* (Günther). Litteratura Serpentium [English Ed.] 6: 46–55.

Kramer E (1977) Zur Schlangenfauna Nepals. Revue suisse de zoologie 84(3): 721–761.

Kuttalam S, Santra V, Owens JB, Selvan M, Mukherjee N, Graham S, Togridou A, Bharti OK, Shi J, Shanker K, Malhotra A (2022) Phylogenetic and morphological analysis of *Gloydius himalayanus* (Serpentes, Viperidae, Crotalinae), with the description of a new species. European Journal of Taxonomy 852: 1–30. https://doi.org/10.5852/ejt.2022.852.2003

Lawrence WR (1895) The valley of Kashmir. Henry Frowde, London, (4), 478 pp.

Manhas A (2020) Observations of Himalayan Pitvipers, *Gloydius himalayanus* (Günther 1864), in the Doda District, Jammu and Kashmir, India. IRCF Reptiles & Amphibians 27: 476–478.

Manhas A, Raina R, Wanganeo A (2018) Reptilian diversity and distributions in the Doda district of Jammu and Kashmir, India. IRCF Reptiles & Amphibians 25: 164–169.

Masroor R (2017) Environmental Baseline studies of Kaghan Temperate Coniferous Forest Landscape with reference to amphibians and reptiles. United Nations Development Programme (report), 25 pp.

McMahon AH (1899) Notes on the fauna of the Gilgit district. Journal of the Asiatic Society of Bengal 68(2): 105–109.

Metha HS (2000) Reptilia. In: Alfred JRB (Ed.) Fauna of Renuka Wetland. Wetland Ecosystem Series 2. Zoological Survey of India, Calcutta, 163–168.

Minton SA jr (1966) A contribution of the herpetology of West Pakistan. Bulletin of the American Museum of Natural History 134(2): 27–184, pl. 9–36.

Murthy TSN, Sharma BD (1976) A contribution to the herpetology of Jammu and Kashmir. British Journal of Herpetology 5: 533–538.

Nanhoe LMR, Ouboter PE (1987) The distribution of reptiles and amphibians in the Annapurna-Dhaulagiri region (Nepal). Zoologische Verhandelingen 240: 1–100.

Negi RK, Banyal HS (2016) A preliminary study of herpetofauna of Rakchham-Chhitkul Wildlife Sanctuary in Trans-Himalayan Baspa (Sangla) valley, district Kinnaur, Himachal Pradesh, India. Journal of Research in Humanities and Social Science 4(11): 145–149.

Sahi DN (1979) A contribution to the herpetology of Jammu and Kashmir State. Department of Biosciences, University of Jammu, India (Ph.D. Thesis).

Sahi DN, Duda PL (1985) A checklist and key to the amphibians and reptiles of Jammu and Kashmir State, India. Bulletin of the Chicago Herpetological Society 20: 86–97.

Schleich HH, Kästle W [Eds] (2002) Amphibians and reptiles of Nepal. Biology, systematics, field guide. Gantner, Ruggell, 1201 pp.

Sclater W (1891) List of Snakes in the Indian Museum. Baptist Mission Press, Calcutta, x, 79 pp.

Shabir S (2022) Molecular identification of collected snake species from district Bagh Azad Kashmir. Institute of Zoological Sciences, University of Peshawar, xi, 121 pp.

Shah KB (1995) Enumeration of habitat, geographical and altitudinal distribution of the amphibians and reptiles of Nepal. Arnhem, Euroconsult, (2), vii, 60, Appendix i–iv.

Sharma S (2004) Observation on some snake species of Kedernath WLS, Garhwal Himalaya. Reptile Rap, Coimbatore 6: 3.

Showler DA (1998) Herpetofauna observations in Palas valley, North-West Frontier Province, Pakistan. British Herpetological Society Bulletin 65: 26–32.

Smith MA, Battersby JC (1953) On a collection of amphibians and reptiles from Nepal. Annals and Magazine of Natural History Ser. 12, 6: 702–704.

Steindachner F (1869) Reptilien. In: Wüllerstorf-Urbair B von (Ed.) Reise der österreichischen Fregatte Novara um die Erde in den Jahren 1857, 1858, 1859. Zoologischer Teil. Erster Band (Wirbelthiere) 3. Kaiserlich-Königliche Hof- und Staatsdruckerei, Wien, 1–98, pl. 1–3.

Stoliczka F (1866) Einige Beobachtungen über den Charakter der Flora und Fauna in der Umgebung von Chini, Province Bisahir, im nordwestlichen Himalaya-Gebirge. Verhandlungen der kaiserlich-königlichen zoologisch-botanischen Gesellschaft in Wien 16: 849–878.

Stoliczka F (1870) Observations on some Indian and Malayan amphibia and reptilia. Journal of the Asiatic Society of Bengal 1870 39(2): 134–157, pl. ix, (3): 159–228, pl. x–xii.

Sura P (1987) [Notes on amphibians and reptiles of the Annapurna region (central Nepal)]. Przegląd Zoologiczny 31(4): 503–509 [In Polish with English summary].

Telford SR III (1980) Notes on *Agkistrodon himalayanus* from Pakistan´s Kaghan Valley. Copeia [1980]: 154–155.

Theobald W (1868) Catalogue of Reptiles in the Museum of the Asiatic Society of Bengal. Journal of the Asiatic Society of Bengal, Extra Number, vi, 82 pp., Appendix i–iii.

Vigne GT (1842) Travels in Kashmir, Ladak, Iskardo, the countries adjoining the mountain-course of the Indus, the Himalaya, north of Panjab. Volume II. Henry Colburn, London, x, 462, (1) pp.

Vogel G (2006) Venomous snakes of Asia / Giftschlangen Asiens. Edition Chimaira (Terralog), Frankfurt am Main, 148 pp.

Wall F (1899) Notes on 26 specimens of the pohur, or Himalayan viper (*Ancistrodon himalayanus*). Journal of the Bombay Natural History Society 12(2): 411–414.

Wall F (1907) Ophidia. In: Boulenger GA, Annandale N, Wall F, Tate Regan, C (Eds) Reports on a collection of batrachia, reptiles and fish from Nepal and the Western Himalayas. Records of the Indian Museum 1, 155–157.

Wall F (1911) Reptiles collected in Chitral. Journal of the Bombay Natural History Society 21(1): 132–145.

Supplementary material 2

Figure S1. Skull of the female holotype of *Gloydius hazarensis* sp. nov., UF 70652, representing the main characters measured in this study: A. dorsal, and B. ventral view (lower jaws virtually extracted). Abbreviations: **Boc** – basioccipital, **Bsp** – basisphenoid, **Ecpt** – ectopterygoid, **Eoc** – exoccipital, **Fro** – frontal, **Max** – maxilla, **Nas** – nasal, **Pal** – palatine, **Par** – parietal, **Por** – postorbital, **Prfr** – prefrontal, **Pro** – prootic, **Prmx** – premaxilla, **Psp** – parasphenoid, **Pte** – pterygoid, **Qua** – quadrate, **Smx** – septomaxilla, **Soc** – supraoccipital, **Sut** – supratemporal, **Vom** – vomer. Measurement: **HL** – head length.

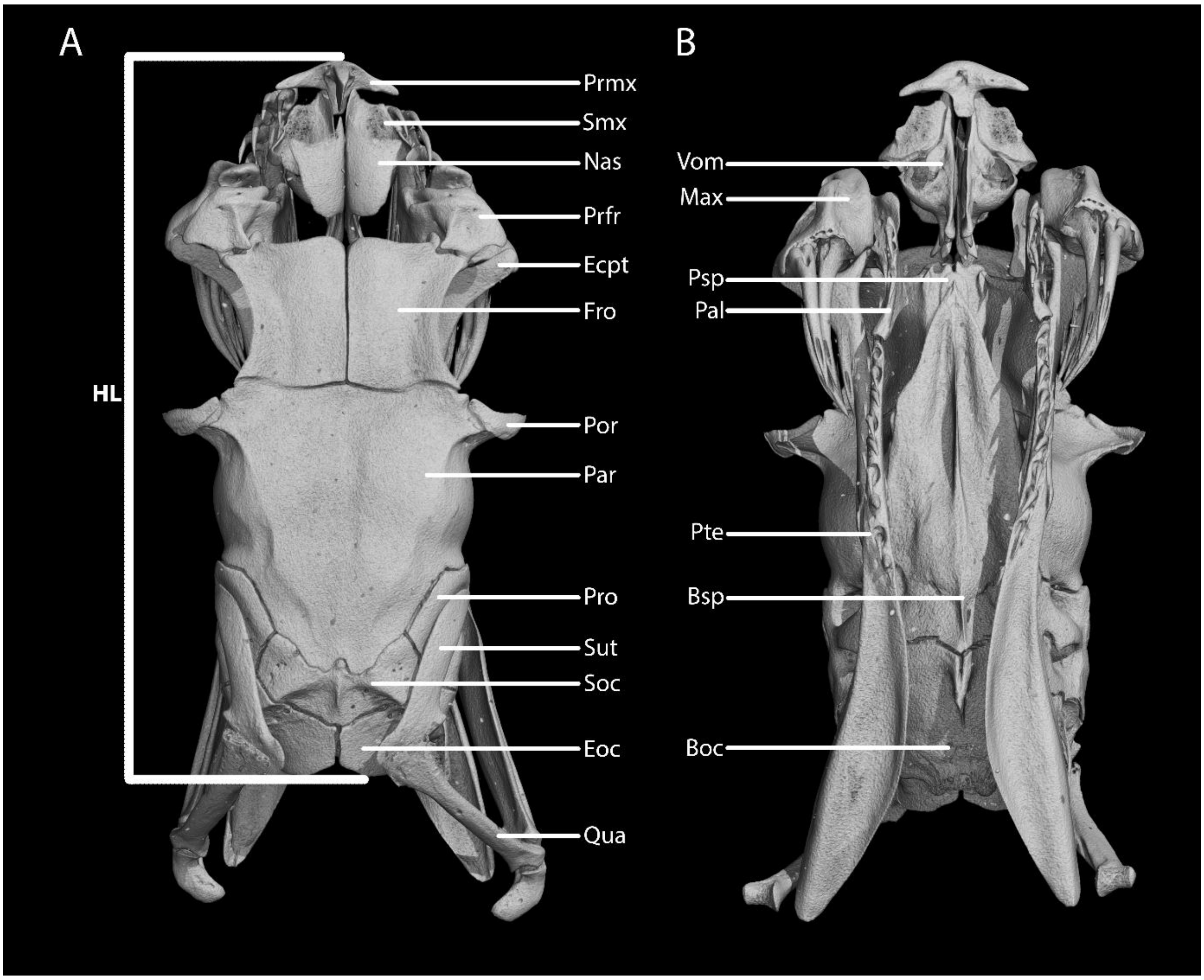

Figure S2. Skull elements using the example of the female holotype of *Gloydius hazarensis* sp. nov., UF 70652, representing the main characters measured in this study: A. pterygoid ventral view, B. lower jaw lateral view, C. lower jaw medial view, D. maxilla with fang lateral view, maxilla with fang ventral view. Abbreviations: **Ang** – angular, **Cpb** – compound bone, **Dent** – dentary, **Spl** – splenial. Measurements: **CpbL** – compound bone length, **DL** – dentary length, **FL** – fang length, **OL** – discharge orifice length, **PteS** – length of pterygoid shield (without teeth), **PteL** – pterygoid length, **SAL** – total length of splenial and angular.

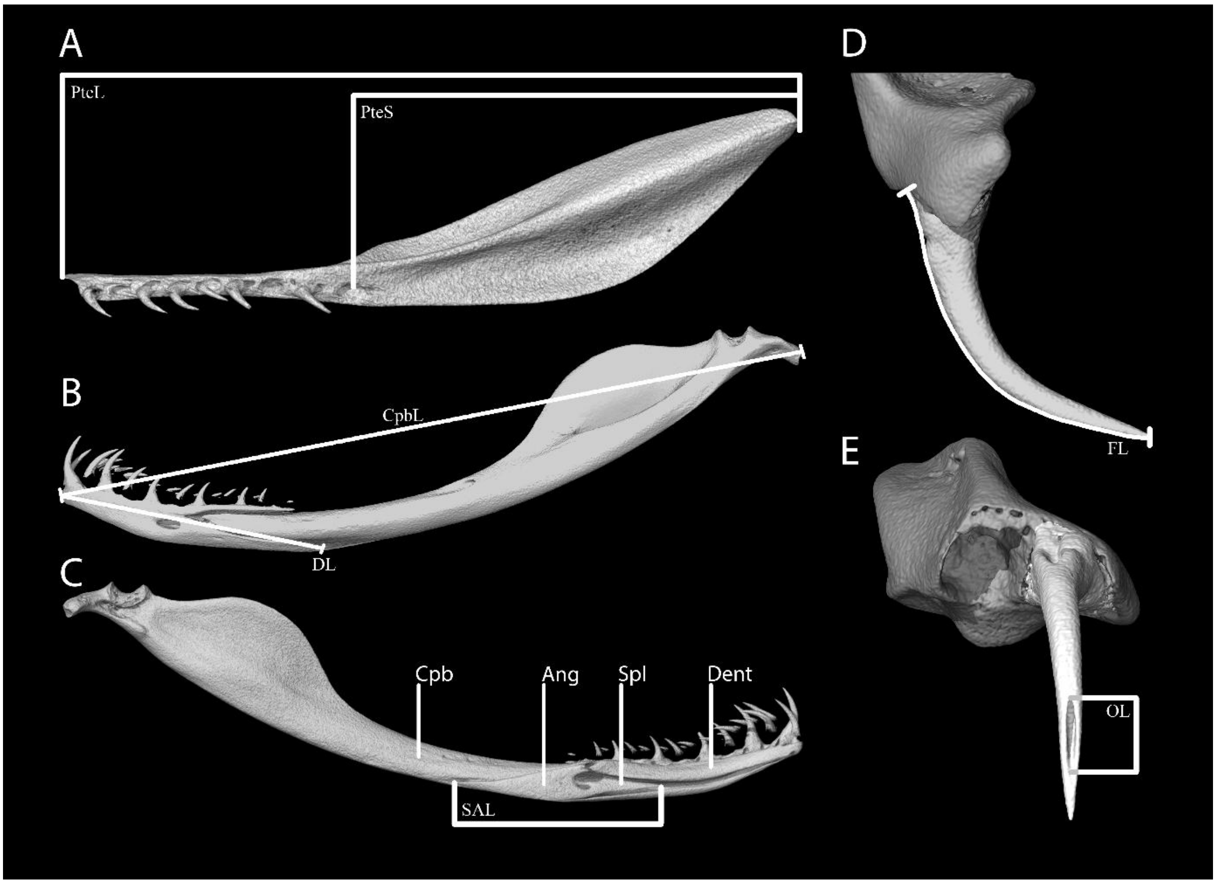

Figure S3. Maximum likelihood phylogenetic tree of the genus *Gloydius* inferred using IQ-TREE based on concatenated mitochondrial and nuclear DNA sequences. Node support values are presented as SH-aLRT/ultrafast bootstrap (UFBoot) percentages. Terminal labels include specimen identifiers (see Supplementary material 1, Table S1).

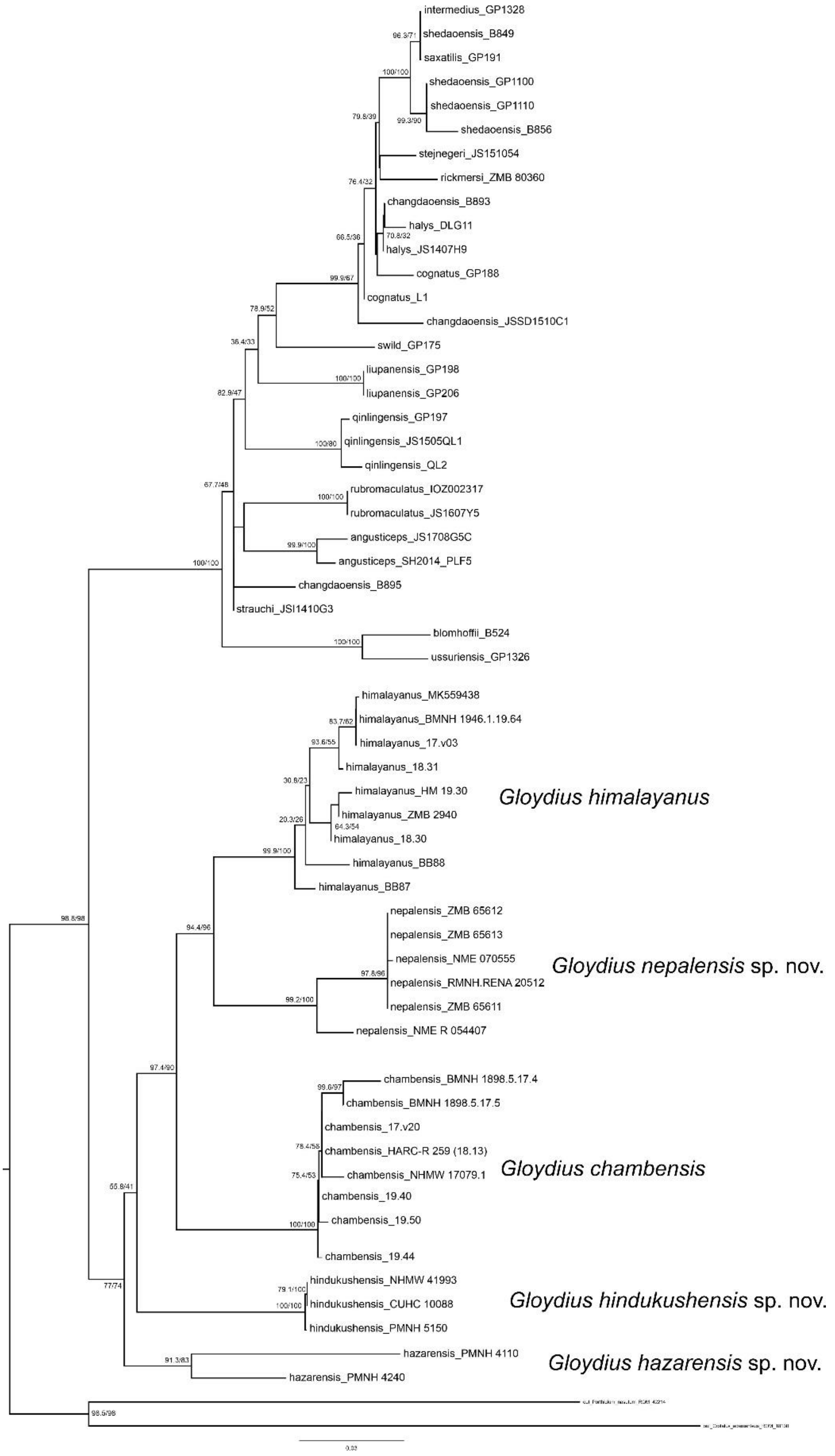

Figure S4. Maximum likelihood phylogenetic tree of the genus *Gloydius* inferred using IQ-TREE based on concatenated mitochondrial DNA sequences. Node support values are presented as SH-aLRT/ultrafast bootstrap (UFBoot) percentages. Terminal labels include specimen identifiers (see Supplementary material 1, Table S1).

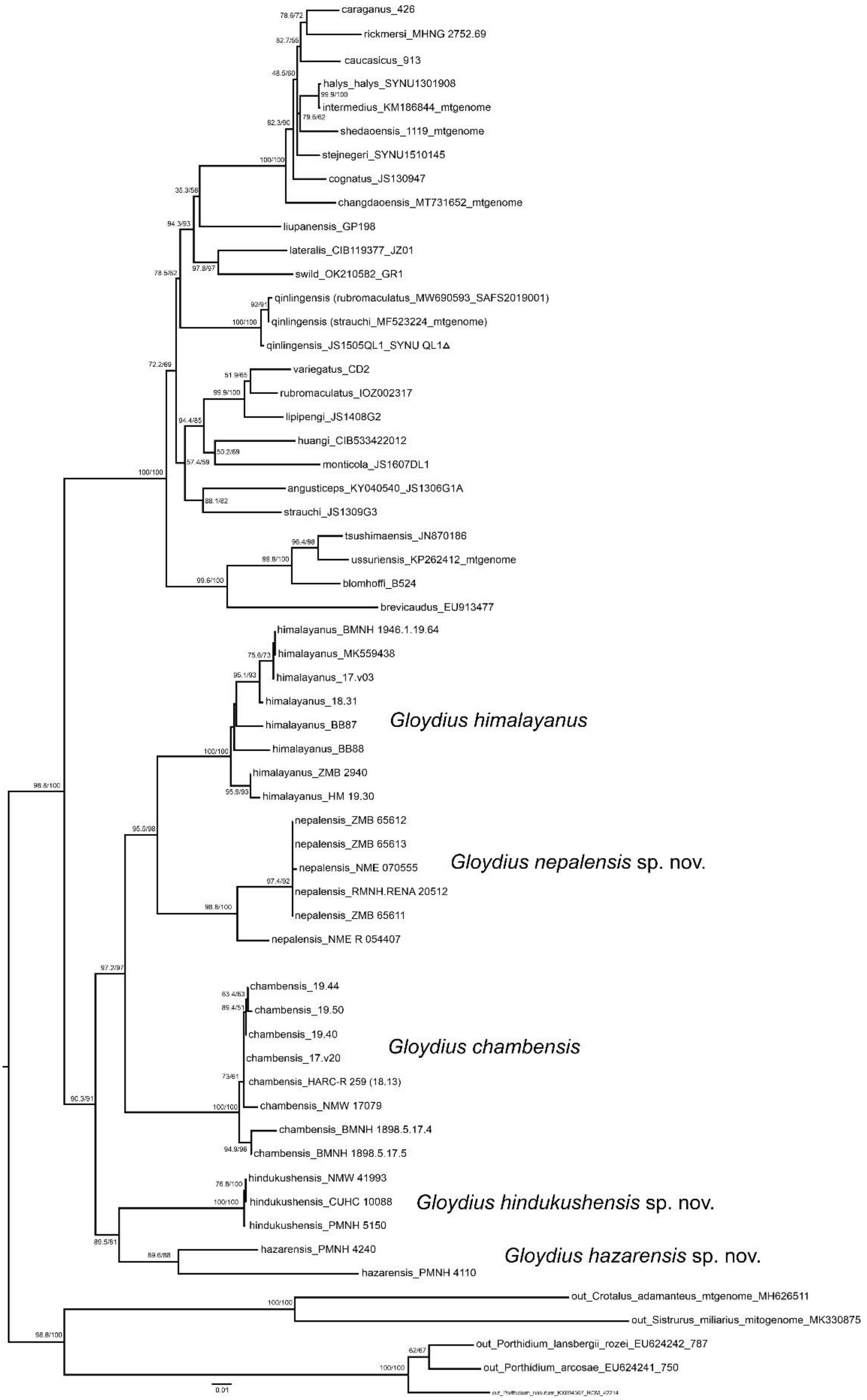

Figure S5. Bayesian phylogenetic tree of the genus *Gloydius* inferred from concatenated mitochondrial and nuclear DNA sequences. Posterior probability values are shown at each node. Terminal labels include specimen identifiers (see Supplementary material 1, Table S1).

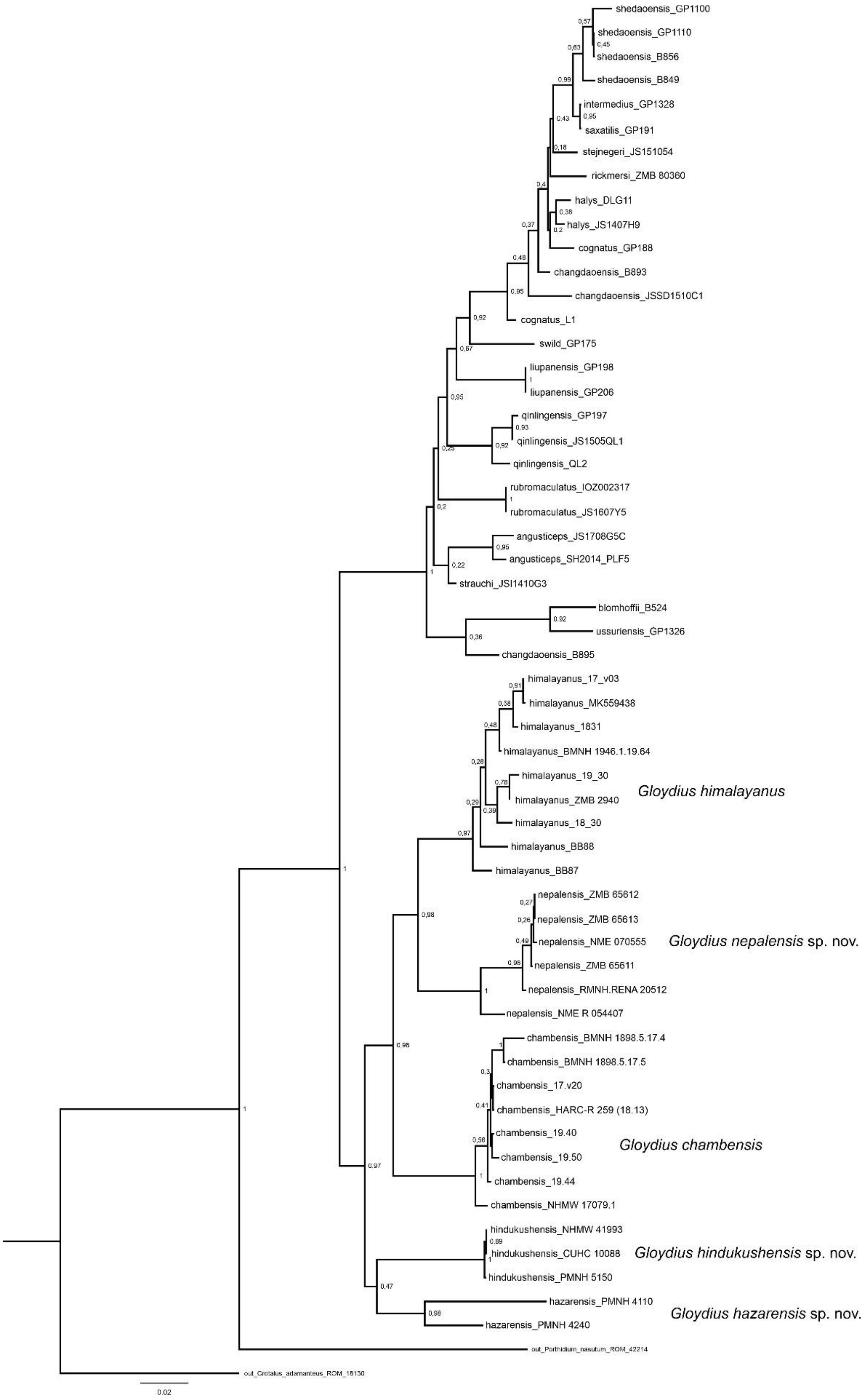

Figure S6. Bayesian phylogenetic tree of the genus *Gloydius* inferred from concatenated mitochondrial DNA sequences. Posterior probability values are shown at each node. Terminal labels include specimen identifiers (see Supplementary material 1, Table S1).

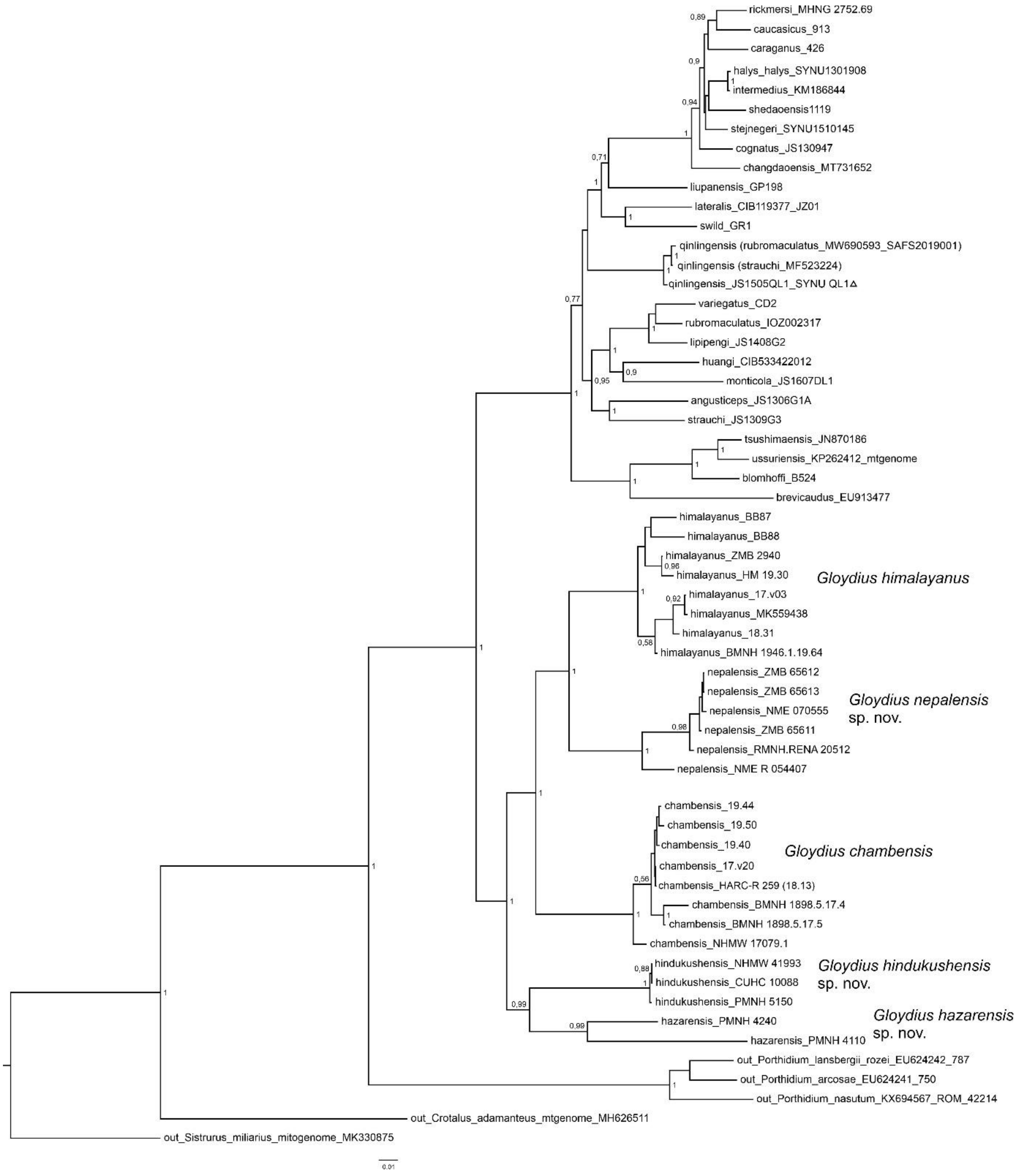

Supplement: Supplementary material 1 — Additional information [file zookeys-1280-083_article-182768__-s001.pdf]
